# Microbial Transformation of 6‑PPDQ in Surface Water, Water–Sediment, and Soil—Results from Simulation Tests with Radiolabeled Substrate

**DOI:** 10.1021/acsenvironau.6c00125

**Published:** 2026-08-08

**Authors:** Thorsten Reemtsma, Bettina Seiwert, Pete Yeomans, Rory Mumford, Sara Bennett

**Affiliations:** † Department of Environmental Analytical Chemistry, Helmholtz Centre for Environmental Research−UFZ, Permoserstrasse 15, 04318 Leipzig, Germany; ‡ Institute for Analytical Chemistry, University of Leipzig, Linnéstrasse 3, 04103 Leipzig, Germany; § Environmental Risk Sciences Division, Smithers, 108 Woodfield Drive, Harrogate HG1 4LS, United Kingdom

**Keywords:** 6PPD-Q, tire and road wear particles, TRWP, leaching, OECD 307, OECD 308, OECD 309

## Abstract

6-PPDQ, an oxidation product of the antioxidant 6-PPD added to tire rubber, reaches the environment with tire abrasion on roads. Firm data on the environmental persistence of 6-PPDQ in different compartments is lacking. The microbial degradation of 6-PPDQ was studied in simulation tests under standardized conditions in the laboratory. Biodegradation half-life of 6-PPDQ (DT50) increased from surface water (12–17 d) over water-sediment systems including nonextractable residue (NER I) under aerobic conditions (46–55 d), soils including NER I under aerobic conditions (27–141 d, median 61 d), water–sediment systems under anaerobic conditions (140–202 d) to soil under anaerobic conditions (214–887 d, >10 000 d in one model). On this basis, 6-PPDQ would be considered nonpersistent according to ECHA’s definition under aerobic conditions in water, in water–sediment systems, and in soil (median value and three of the four tested soils). 34% of the applied radioactivity (AR) of [^14^C]-6-PPDQ was mineralized under aerobic conditions in 60 d in surface water, 27% in the water–sediment system in 100 d and 34% in soil in 120 d. Under anaerobic conditions, mineralization was 4%–5% AR, only, both in water–sediment and in soil. Hydroxylated and carboxylated 6-PPDQ were identified as transformation products, of which 11% AR remained after 60 d. In sediment and soil no single transformation product exceeded 1% AR at the end of the experiment; instead, NERs were formed. Under aerobic conditions, 55% (water–sediment) and 49% (soil) of the AR of degraded 6-PPDQ occurred as NER; it increased to 67% (water–sediment) and 84% (soil) under anaerobic conditions. These first quantitative data on the extent and the rate of biodegradation and mineralization of 6-PPDQ help to understand the occurrence of 6-PPDQ in the environment and support environmental exposure modeling and risk assessment.

## Graphical abstract

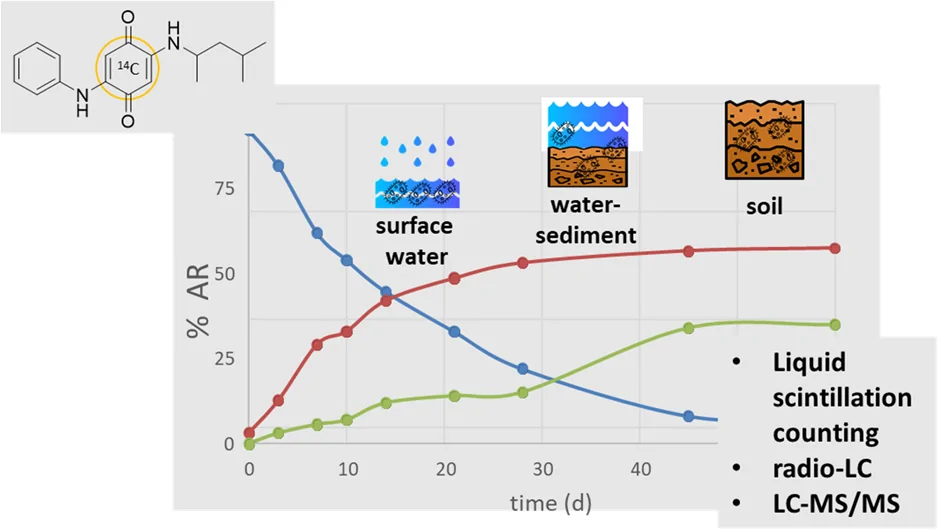

## 1 Introduction

The antioxidant 6-PPD is an important additive in tire rubber to prevent the oxidation of rubber and to increase the lifetime of tires. However, one of its oxidation products, 6-PPDQ, has been shown to exert a high toxicity to certain fish species.^1,2^ This, together with reports on the occurrence of 6-PPDQ not only in road environment but also in receiving waters, sediments, and marine environment,^3–6^ has led to a drastic increase in scientific scrutiny and also to regulatory attention.^7,8^ It fueled many studies on the biological effects of 6-PPDQ.

Knowledge on the environmental persistence of 6-PPDQ is still fragmentary, although persistence is a primary determinant for environmental exposure. Biodegradation by microorganisms is a major process limiting the lifetime of chemicals in the environment; it may lead to the formation of transformation products and, eventually, to mineralization of a chemical. In biodegradation studies, it is important to not only follow the removal of the test compounds but also the formation of transformation products,^9^ although their detection, identification, and quantification are often hampered by the lack of reference compounds. The use of “hot”, radiolabeled test compounds can help in this situation, as it allows to detect and to quantify transformation products by their ^14^C-label with radio-HPLC, even if they remain unidentified. This approach has only recently been used to study the abiotic transformation of [^14^C]-labeled 6-PPDQ and 6-PPD.^10^

For biodegradation experiments under standardized conditions, a hierarchy of OECD tests has been established to meet regulatory data requirements: it starts with easy-to-perform “screening tests” for ready biodegradability and moves on to more meaningful simulation tests. These simulate conditions of specific environmental compartments such as surface water,^11^ water-sediment systems,^12^ and soils.^13^

When working with [^14^C]-labeled test compounds in heterogeneous systems such as sediments and soils, it must be considered that microbial activity can support the formation of nonextractable residues (NERs), either of the parent compound or its transformation products.^14^ NER may be further distinguished as (a) physically sequestered or entrapped residues with the potential to become released, (b) covalently bound to organic matter or to biological tissue, or (c) incorporated into biogenic organic matter.^15^

Studies on the microbial degradation of 6-PPDQ are quite rare. Only one study in a homogeneous aqueous system (solution) is available.^16^ A biodegradation half-life of 3 ± 1 d in aqueous solution was derived in that study, but not under standardized (OECD) test conditions and with a comparatively high biomass density. Several transformation products could be detected and identified but were not quantified due to the lack of reference material. Other studies have followed the removal of 6-PPDQ from solution in leachates of cryo-milled tire tread (CMTT), crumb rubber, or tire-and-road-wear-particles (TRWP).^17–19^ While these studies have testified that 6-PPDQ is biodegradable, they are not suited to derive biodegradation half-lives for the free 6-PPDQ as 6-PPDQ is continuously released from the tire particles into solution, which leads to extended half-lives.^19^ For soils, the biodegradation of 6-PPDQ has been studied under aerobic and anaerobic conditions, with the substrate added as pure 6-PPDQ or in tire particles.^20,21^ Also, for sediment, one study on anaerobic biodegradation is available.^20^

Thus, there is a lack of high-quality persistence data for 6-PPDQ for various environmental compartments, namely, of kinetic biodegradation data (biodegradation half-life). This hampers the environmental risk assessment of 6-PPDQ and, indirectly, also that of its parent compound 6-PPD.

This study reports results on the kinetics and pathway of biodegradation of 6-PPDQ with a [^14^C] label in soil, surface water, and water-sediment systems in simulation tests according to the respective OECD guidelines, under aerobic and anaerobic conditions. The data support environmental risk assessment of 6-PPDQ for different terrestrial and aquatic compartments into which 6-PPDQ may be introduced as a consequence of tire abrasion on roads.

## 2 Materials and Methods

### 2.1 Test Specimen and Samples

[^14^C]-labeled 6-PPDQ (CAS No. 2754428–18–5) with the central phenylendiamine-ring [^14^C]-labeled was obtained from Pharmaron UK Ltd. (Cardiff, UK) and was stored frozen (nominally −20 °C) in the dark. The radiochemical purity was of 98% (own analyses) (see the Supporting Information, Section S1.1).

Natural surface water was sampled from Oak Beck, near Harrogate, U.K. Details on sampling and sample properties are provided in Table S2. The water was collected straight into plastic containers. Prior to characterization and dispensing, the water was passed through a 100 μm sieve and stored in the dark in an environmental chamber routinely maintained at 4 ± 2 °C. A subsample of the filtered water was autoclaved for use in the sterile incubation group.

Samples of two sediment–water systems differing in stream characteristics and texture were used in this study (Tables S3–S5). Prior to use, the sediment and waters were stored together, water-logged (ca. 6–10 cm water layer), in an environmental chamber routinely maintained at 4 ± 2 °C.

Four soils of different textures and organic carbon contents were used in this study to cover a wide range of soils. More details are provided in Tables S6–S8.

### 2.2 Experiments

#### 2.2.1 Surface Water

The experiments on biodegradation of [^14^C]-6-PPDQ in surface water were performed in accordance with OECD 309 ^11^ for 60 d at 20 ± 2 °C, using the test system shown in Figure S1. [^14^C]-6-PPDQ was dispensed with 18 vessels in incubation groups A (1.5 μg/L 6-PPDQ) and B (15 μg/L 6-PPDQ), and two vessels in incubation group C (15 μg/L 6-PPDQ, sterile) and group D (10 μg/L sodium benzoate).

At day 0 (immediately after treatment) and at day 7, 14, 21, 28, 45, and 60 two vessels were taken from group A and B; additional intervals of 3 and 10 days were dispensed and treated with radiolabeled test substance on a separate occasion. Group C was sampled on day 60 and group D on day 0 and 60. From group D samples 63% of AR of sodium benzoate (10 μg/L) was determined from the NaOH trap (most likely as CO_2_) after 4 days of incubation. This shows that the microbial community of the surface water was active. More details are provided in the Supporting Information, section 1.2.1.

#### 2.2.2 Water–Sediment System

Biodegradation of [^14^C]-6-PPD in a water–sediment system was studied under aerobic and anaerobic conditions using two different sediment samples for 100 d, according to OECD 308.^12^ The test setup for the aerobic and the anaerobic experiments is shown in Figures S2 and S3. Sediment samples were dispensed into individual incubation vessels (diameter 4.5 cm) to a depth of 3 cm, and the associated overlying water was added to the vessel to a depth of 9 cm above the sediment.

All sediment vessels were maintained in the dark, at 20 ± 2 °C and on an orbital shaker for the aerobic vessels. At intervals of 0 (immediately after treatment), 7, 14, 29, 61, and 100 d, duplicate vessels from each water–sediment system treated with radiolabeled 6-PPDQ were removed for analysis. More details are provided in the Supporting Information, section S1.3.1.

#### 2.2.3 Soil

The test setups shown in Figures S4 and S5 were used and samples of four different soils (100 g, dry weight equivalent) were dispensed into individual glass vessels (250 mL) and maintained in the dark at 20 ± 2 °C for up to 120 days after addition of [^14^C]-6-PPDQ under aerobic and anaerobic conditions, according to OECD 307.^13^ For the anaerobic test, aerobic incubation was performed first after addition of [^14^C]-6-PPDQ for the DT50 of the test material or for 30 days (Table S9, dependent upon soil, and the samples were then flooded and kept in the system for up to 120 d.

Duplicate aerobic vessels were taken for analysis at intervals of 0 (immediately after treatment), 7, 14, 30, 62, and 120 d. Of the anaerobic experiment, duplicate vessels were taken for analysis immediately after treatment, on the day of flooding (0 DAF), and at 7, 14, 29, 60, and 120 DAF (days after flooding). More experimental details are provided in the Supporting Information, Section S1.4.1.

### 2.3 Sample Processing

#### 2.3.1 Surface Water

After sampling, the content of the incubation vessel was transferred to another vessel and the original vessel washed with acetonitrile and water. All of these solutions were combined. An aliquot was analyzed by LSC, and another aliquot was reduced in volume and subjected to radio-HPLC analysis. More details are given in the Supporting Information, Section S1.2.2.

#### 2.3.2 Sediment–Water System

The overlying water was decanted into acetonitrile prior to quantification by LSC. Samples were concentrated if required prior to radio-HPLC. Sediments were extracted three times by LSE with acetonitrile and the extracts analyzed by LSC. An aliquot was concentrated and analyzed by radio-HPLC.

The extracted sediment samples collected at the end of the experiment (100 d) were air-dried after LSE, and an aliquot was subjected to NER fractionation, as proposed by ECHA.^15^ This procedure consists of harsh microwave-assisted extraction (MAE) with acetonitrile/water to extract the remaining available material, followed by two other procedures applied to parallel aliquots: a silylation procedure, to liberate physically bound test compounds or their transformation products (NER type I), and an acid reflux extraction of biogenic matter (NER type III). Details are provided in the Supporting Information, Section S1.3.2.

#### 2.3.3 Soil

After collection, soil samples were extracted repeatedly by LSE, first with water/acetonitrile and then with pure acetonitrile. These extracts were then combined. Samples taken at the end of aerobic or anaerobic incubation were also subjected to the NER fractionation with MAE followed by silylation and acid reflux applied in parallel, as described above. For details, please refer to the Supporting Information, Section S1.4.2.

### 2.4 Analysis

#### 2.4.1 Liquid Scintillation Counting

Total radioactivity of samples was measured by liquid scintillation counting (LSC) from all water samples and extracts (details given in the Supporting Information, Section S1.5.1). Particulate samples (sediments and soils) after LSE were combusted to quantify, before and after each step of extraction (for NER determination and characterization). The combusted products were absorbed in Hidex Radiocarbon, and the radioactivity was determined by LSC. Radioactivity in the trapping solutions of the gas outlets of the test vessels was quantified by LSC when the vessels to which they were attached were removed for analysis or every 30 to 40 days. Fresh traps were added to those vessels, continuing incubation.

#### 2.4.2 Radio-HPLC

Sample extracts were analyzed by reversed-phase liquid chromatography with radioactivity detection using a water/methanol gradient with 0.1% formic acid. UV absorbance was monitored at 358 nm and radioactivity using a βram, Lablogic detector. Details are provided in the Supporting Information, Section S1.5.2. All results given as percent values refer to the applied radioactivity (AR) of 6-PPDQ of the respective experiment.

#### 2.4.3 LC-MS/MS

Identification of transformation products was conducted by LC-MS/MS analysis using the same chromatographic conditions, with electrospray ionization operated in positive and negative modes. Nonlabeled reference compounds were available for 6-PPDQ, 4-aminophenol, *N*-phenyl-*p*-phenylene amine, aniline, and 4-hydroxydiphenylamine (4-HDPA) (Table S1). More details are provided in the Supporting Information, Section S1.5.3.

### 2.5 Kinetic Data Analysis

From the concentration data of [^14^C]-6-PPDQ in surface water, degradation kinetics were calculated according to single-first-order (SFO) kinetics using the software CAKE version 2.

For the water–sediment system, the rate of transformation of [^14^C]-6-PPDQ was modeled three times: for the concentration in the overlying water, for the sediment as obtained from LSE, and for the total system. For the latter, the following fractions of 6-PPDQ were summed: the overlying water, the sediment extract obtained by LSE, and the percentage of NER that was recovered by MAE and as NER type I. The first-order multicompartment (FOMC) model was selected based on best-fit kinetics (visual fit and χ^2^-values).

For the experiments in soil, degradation kinetics were calculated for two fractions: for the [^14^C]-6-PPDQ extracted by LSE and for the sum of [^14^C]-6-PPDQ extracted by LSE plus the percentage of NER recovered by MAE and as NER I. Single first-order (SFO), first-order multicompartment (FOMC), and double first-order in parallel (DFOP) models were used to calculate DT50 values; the calculations were made using CAKE version 3.6 software.

## 3 Results and Discussion

### 3.1 Aerobic Degradation in Surface Water

#### 3.1.1 Extent and Kinetics of Biodegradation

The aerobic degradation of 6-PPDQ was studied in a natural surface water sample at two concentration levels (1.5 μg/L, 15 μg/L) over 60 d. 6-PPDQ was degraded almost completely during this time, with only 6.6%–7.0% of the AR remaining. A proportion of 30%–38% AR was determined in the CO_2_ traps (Figure 1) and 57%–58% AR as transformation products (see section 3.1.2). The sterile control showed less degradation of 6-PPDQ, with 70% AR remaining as 6-PPDQ and 9% as CO_2_ after 60 d. Most of this decrease is, likely, due to abiotic oxidation, as 6-PPDQ is not prone to hydrolysis.^10^ Recovery of total AR in these incubation experiments ranged from 93% (sterile control) to 102% (1.5 μg/L 6-PPDQ) (details given in Tables S11 and S12) and was, thus, in the range recommended by the OECD guidelines.^11^

**Figure 1:**
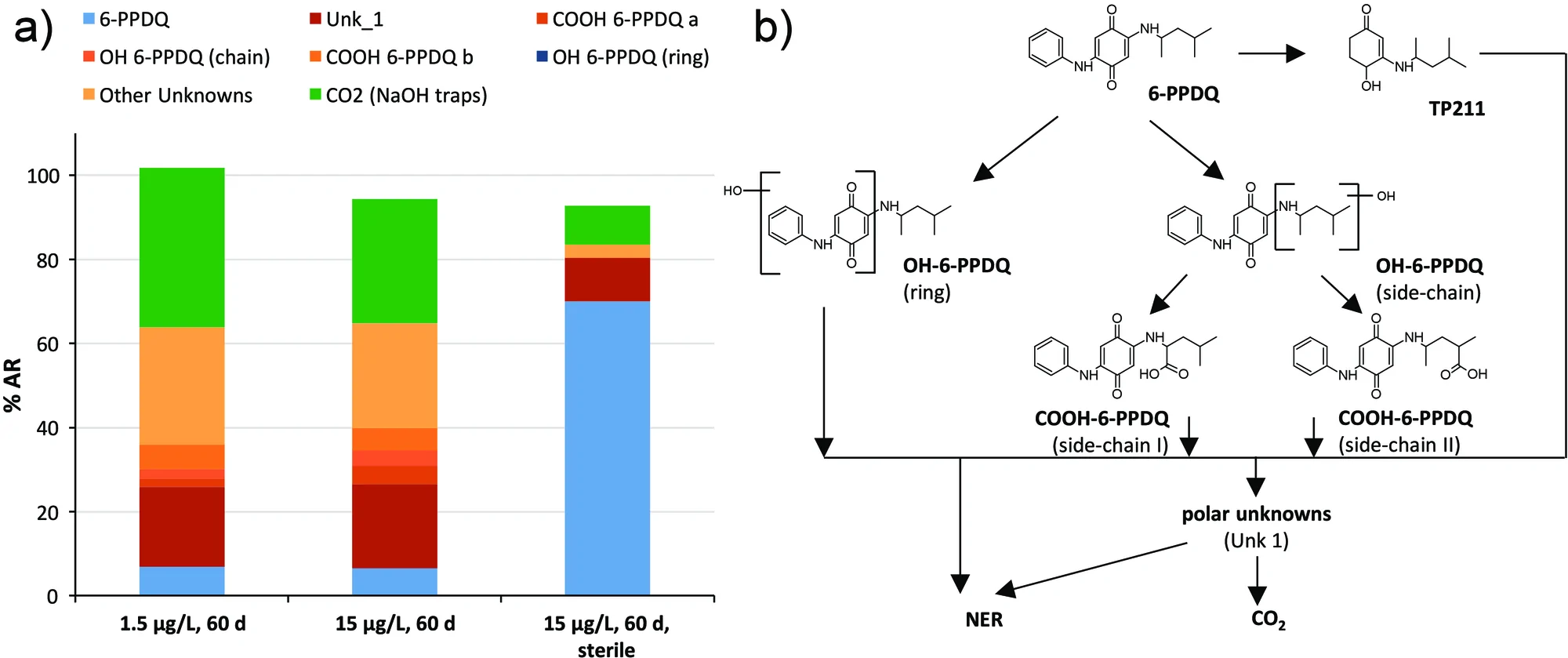
(a) Distribution of applied radioactivity (% AR) of 6-PPDQ after 60 d of aerobic biodegradation in surface water (OECD 309). (b) Proposed biodegradation pathway of 6-PPDQ based on the ^14^C-labeled transformation products identified by LC-MS/MS (for MS details, refer to Table S15).

The 6-PPDQ degradation recorded in the test period of 60 d over nine samplings followed first-order kinetics, resulting in a half-life of 11.8–16.9 d for the two concentration levels (Table 1). From these data, 6-PPDQ can be considered nonpersistent in freshwater as its biodegradation half-life under aerobic conditions was well below 40 d.^22^

**Table 1:** First-Order Half-Life (DT50) and First-Order Rate Constant (*k*) of 6-PPDQ Biodegradation in Freshwater (OECD 309) for Starting Concentrations of 1.5 and 15 μg/L

	DT50 (d)	*k* (d^–1^)	χ^2^	*r* ^2^
6-PPDQ (1.5 μg/L)	11.8	0.05894	8.27	0.9638
6-PPDQ (15 μg/L)	16.9	0.04102	7.54	0.9338

In a previous study aerobic biodegradation of 6-PPDQ dissolved in water by activated sludge at a substrate:biomass ratio of 100 μg/0.3 g of sludge resulted in a significantly shorter half-life of 3 ± 1 d.^16^ In another study, 6-PPDQ was removed from an inoculated leachate by approximately 50% within 1–2 days.^18^

Recently, half-lives of 6-PPDQ for abiotic transformation processes have been determined;^10^ together with this data on microbial degradation in water, one can now assess which transformation process is, likely, the most important one. With a median DT50-value of 466 d in natural waters at 25 °C, hydrolysis is not a major process. On the contrary, transformations induced by sunlight in the presence of oxygen or ozone effectively transform 6-PPDQ (DT50 = 0.1–0.5 d)^10^ but these processes are expected to be less relevant in natural waters. On this basis, microbial degradation is expected to play the major role in 6-PPDQ degradation in natural waters.

The actual degradation rate in a specific natural system may differ from the DT50 = 12–17 d derived from the standardized simulation test (Table 1) and will be influenced by factors such as biomass density, biomass:substrate ratio, temperature, and the composition of the microbial community. Anyhow, long-range transport of 6-PPDQ through rivers into marine systems^5^ in dissolved form is not very likely, given its strong sorption tendency (log *K*_ow_ = 4.54).^23^ Instead, 6-PPDQ may be transported over longer distances in aqueous systems if stabilized by adsorption to suspended matter or in TRWP.^19^

#### 3.1.2 Transformation Products and Pathway

Several transformation products of 6-PPDQ were detected by radio-HPLC, and several products were tentatively identified by LC-MS/MS (Table S15). Radioactivity detection allowed us to quantify them in terms of AR initially added as 6-PPDQ provided that a suitable chromatographic signal was recorded (Figure 1a). It should be noted that detection of transformation products is based on their ^14^C label. Products formed from nonlabeled substructures of 6-PPDQ would not be covered by this analysis.

Hydroxy-6-PPDQ (ring) is an important early intermediate transformation product with a maximum of 13% AR at day 7; it was degraded to below LOD toward the end of the experiment. The isomeric hydroxy-6-PPDQ (chain) remained lower in concentration and never exceeded 5% AR. Two isomeric 6-PPDQ carboxylic acids were detected and may be formed from hydroxy-6-PPDQ (chain); they reached maximum concentrations of 4% and 8% AR at 21 and 45 d (Figure 1a) and then degraded slowly toward 60 d. The polar compound underlying one important early eluting signal in radio-HPLC (Unk-1 with RT 3–4 min) remained unidentified by LC-MS/MS; it may even consist of more than one compound. Unk-1 reached a maximum of 22%–26% AR at day 28 and then decreased slowly to 16%–17% AR at 60 d. Likely, these transformation products were degraded in part into so far “unknown” products; this fraction increased to 28% AR after 60 d (Figure 1a). Total percentage of transformation products of low molecular weight increased up to 57% AR toward the end; 46% AR remained unidentified. Without the ^14^C-label, these products would even have remained undetected.

The identification of biotransformation products of 6-PPDQ is challenged by the complexity of the transformation processes and the large number of transformation products that are formed. Six transformation products of 6-PPDQ have been previously reported in a leachate of tire particles inoculated with activated sludge,^18^ and a total of 26 products were detected and tentatively identified in an aerobic biodegradation experiment with 6-PPDQ in solution by activated sludge under nonstandardized conditions.^16^ Hydroxylated 6-PPDQs (ring and chain) were among those products, with the chain-hydroxylated isomer being very dominant in terms of signal intensity. Another prominent transformation product of that study agreed in mass with 6-PPDQ carboxylic acids (6-PPDQ_328; Figure 1b; Table S15).

Reports on the detection of these transformation products in environmental samples are very scarce, as they have been rarely searched for. Only hydroxylated 6-PPDQ has once been reported in the influent of a wastewater treatment plant.^16^ Inclusion of the hydroxylated and carboxylated 6-PPDQ into monitoring studies would be useful to learn about their occurrence and stability in an aqueous environment.

### 3.2 Water–Sediment System

Several monitoring studies with hundreds of sampling sites have outlined that 6-PPDQ is widely found not only in river sediments but even in coastal sediments.^5,6^ [^14^C]-6-PPDQ biodegradation was studied in the more-complex water–sediment simulation test using sediment and water from two ponds (see section 2.2.2, as well as Tables S3 and S4) under aerobic and anaerobic conditions over a period of 100 days.

#### 3.2.1 Extent and Kinetics of Biodegradation

Half-life calculation in the water–sediment system was performed for the concentrations of 6-PPDQ found in the overlying water, for 6-PPDQ in the whole system (water plus sediment) and for 6-PPDQ and NER I in the whole system (Table 2). [^14^C]-6-PPDQ was introduced into the overlying water of the water–sediment system. Its affinity to organic phases is quite strong (log *K*_ow_ = 4.54).^23^ Therefore, its concentration decrease in the water phase is not solely due to biodegradation but also to transfer into the sediment. Indeed, 40%–50% AR of the 6-PPDQ initially added to the overlying water was found in the sediment after 29 d, with a higher percentage for the sediment with the higher organic carbon content (51%–54% AR for Calwich Abbey, C_org_ 4.1%, Table S4) than for the sediment with the lower organic carbon content and higher sand content (43% AR for Middle Pond, C_org_ 2.9%, Table S4). There was no significant difference in transfer of 6-PPDQ to the sediment for the anaerobic compared to the aerobic conditions.

**Table 2:** Biodegradation Kinetics of 6-PPDQ in the Two Water–Sediment Systems under Aerobic and Anaerobic Conditions Derived from FOMC Modeling (Details in Table S17)

	Water phase		Water and sediment extract (LSE)		Total system (water + sediment (LSE + MAE + NER I)	
	DT50 (d)	DT90 (d)	DT50 (d)	DT90 (d)	DT50 (d)	DT90 (d)
Calwich Abbey aerobic	1.37	14.7	39.5	507	54.6	1600
Middle Pond aerobic	4.43	27.2	30.2	126	46.3	336
Calwich Abbey anaerobic	13.1	112	116	389	140	523
Middle Pond anaerobic	25.1	99.7	131	438	202	686

Half-lives in the overlying water under aerobic conditions were in the range of 1.4–4.4 d. These values are significantly shorter than in pure aqueous solution (Table 1), which may be explained by two factors: (a) more intense biodegradation in the aqueous phase due to a higher biomass density in the water phase of the water/sediment system compared to surface water and (b) removal of 6-PPDQ from the aqueous phase by sorption to the sediment as discussed above. The importance of biodegradation in the overlying water phase is obvious from the significantly longer half-life of 6-PPDQ under anaerobic conditions (Table 2): half-lives in this phase were almost 1 order of magnitude longer, compared to aerobic conditions (Table 2).

Under aerobic conditions, the DT50 values of 6-PPDQ in the water–sediment system were 30–40 d (Table 1). Under anaerobic DT50 values significantly increased to 116–131 d, which is around the persistence threshold of 120 days in the European Union.^22^

The transition of 6-PPDQ from the aqueous into to the sediment phase decreased its biodegradation rate and increased its half-life markedly. Two factors can explain this phenomenon: (a) reduced amenability to microbial biodegradation of a sorbed compared to a freely dissolved substrate and lower oxygen availability in the sediment layer, compared to the overlying water phase, slowing down oxidative transformation. This finding agrees to a previous study on biodegradation of 6-PPDQ from CMTT and TRWP:^19^ in those experiments the pseudo-first-order biodegradation half-life of 6-PPDQ was much longer (80–260 d) then in aqueous solution, found here and reported previously.^16^ This was ascribed to the limited availability of 6-PPDQ in the rubber matrix for microbial degradation.

Under aerobic conditions, the DT90 values for the sediment extract are a factor of 4–12 longer than the DT50 values (Table 2). If a single first-order model would be applicable for the whole period of biodegradation, this factor should be around 3. The higher factors found in this study indicate that biodegradation kinetics was not first-order but slowed down as the substrate concentration decreased. Also this agrees with observations made for biodegradation of 6-PPDQ in a heterogeneous system of CMTT and TRWP in suspension: there, the half-life increased from 80–260 d in a first period of 100 d of biodegradation to 520–1600 d in a second experimental phase, extending to >700 d.^19^ This decreasing degradation rate was ascribed to slow diffusive intraparticle transport of 6-PPDQ toward the particles surface becoming rate-limiting for biodegradation at lower concentrations.

In a recent study on biodegradation of 6-PPDQ under anaerobic conditions, DT50 values of 21–28 d were reported, depending on the presence of different electron acceptors added.^20^ These half-lives are much shorter than the 116–131 d found here for the anaerobic system; rather, they are comparable to the data of the aerobic test system (Table 2). On the basis of the data available, this difference cannot be explained.

It is interesting to note that the redox potential in the sediment of the water–sediment system did not differ much between the aerobic and the anaerobic experiments and was deemed anaerobic in all systems (see the Supporting Information, Section S1.3.1). This may reflect the fact that oxygen in the sedimentary layer is constantly consumed by microbial mineralization of the organic matter of the sediment and that the supply of oxygen from the overlying aqueous phase into the whole sediment layer is slow, even at a dissolved oxygen content of 7 mg/L in the water phase (aerobic system (see the Supporting Information, Section 1.3.1). But why does 6-PPDQ degradation differ in the aerobic and anaerobic water–sediment systems, even though most of the 6-PPDQ is located in the sediment and both are anaerobic? This can be explained assuming that most 6-PPDQ was degraded at the boundary layer between the sediment and the overlying water; here, more oxygen was available in the aerobic system, even though the bulk sediment layer was anaerobic. This would allow for a more rapid 6-PPDQ degradation in the aerobic water–sediment system, even though both bulk sediments were anaerobic.

Formation of NER is a phenomenon well-known for biodegradation processes in heterogeneous systems such as sediments and soils. Conceptually, three different NER fractions have been defined in the context of risk assessment of chemicals. NER I is formed by strong sorption or “entrapment” and may potentially be remobilized if environmental conditions change drastically. NER I determined as [^14^C] by LSC may include the parent 6-PPDQ as well as transformation products. The other two NER fractions, the covalently bound fraction (NER II) and the carbon incorporated into biogenic matter (NER III) are considered irrelevant for exposure assessment.^14,15^ However, a defined operational approach to differentiate between the NER fractions is still under debate.^24^ The same is true with respect to NERs relevance for biological effects.^25^

In this study, NER I was determined from the sediment in a time-resolved manner. Taking this fraction into account in half-life calculation as requested by ECHA,^15^ DT50 values in the water–sediment system increased to 46–55 d under aerobic conditions (Table 2). Under anaerobic conditions, DT50 values increased more strongly to 140–202 d and exceeded the threshold value of 120 days.

#### 3.2.2 Transformation Products

The changes in 6-PPDQ concentration and its distribution in the water–sediment system under aerobic conditions were comparatively similar for the two sediment samples: 6-PPDQ removal reached 71%–84% over 100 days and the extent of mineralization reached 22%–32% AR. With this, 30%–38% of the 6-PPDQ degraded was mineralized, based on the AR (Figure 3). Most of the 6-PPDQ removed was transformed into NER (42–43% of AR), however, corresponding to 51%–58% of the degraded 6-PPDQ. The large extent of formation of NER from 6-PPDQ upon biodegradation in sediments may be explained from the fact that quinones and hydroquinones are prone to radical reactions and Michael addition; both processes facilitate formation of chemical bonds with various organic substrates. Much more natural organic matter is available as reaction partner for such coupling reactions in the sediment layer of the water–sediment test than in the surface water system (Tables S2 and S4).

Transformation products of low molecular weight play a minor role only in the water–sediment system (Figure 2). They comprise a maximum of 7.7% AR under aerobic conditions, compared to 60% AR in the test in surface water (Figure 1a). The higher microbial activity in the water–sediment system may have transformed those compounds more effectively, in part into NERs, so that less of these transformation products remained.

**Figure 2:**
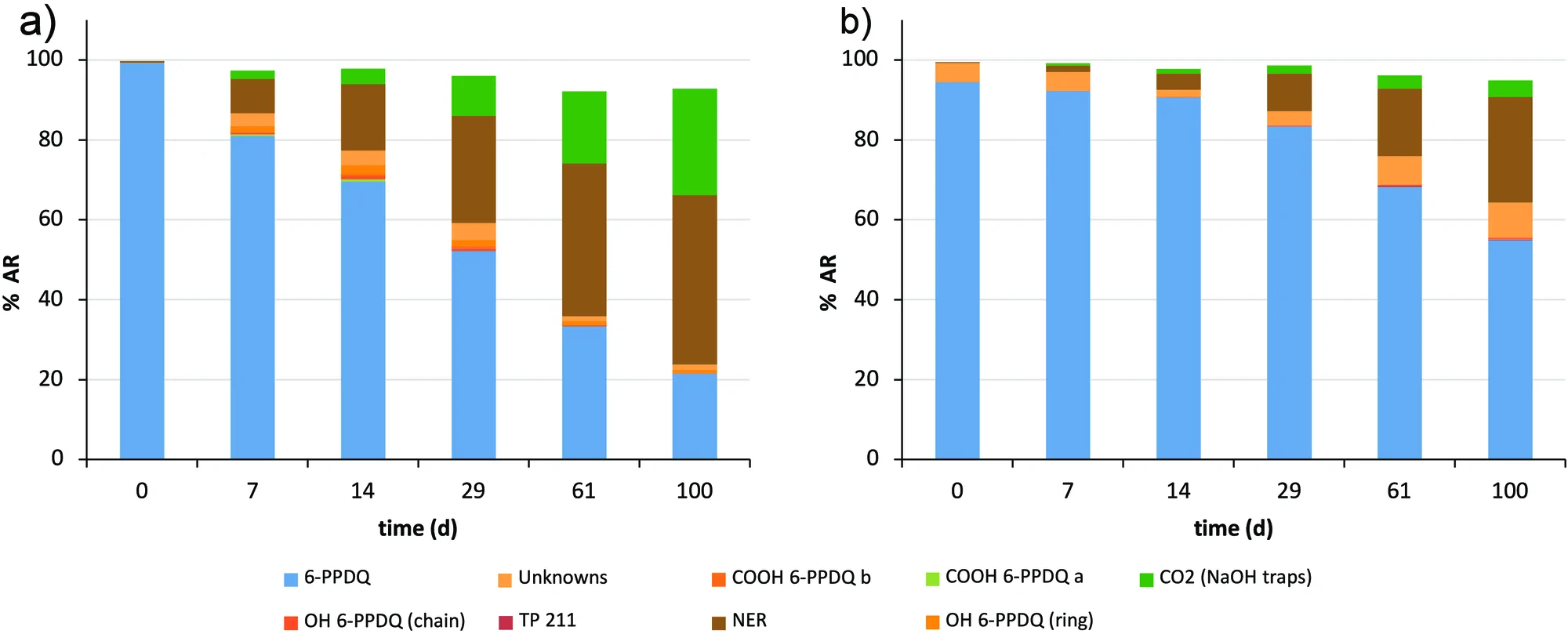
Mean distribution of AR of [^14^C]-6-PPDQ over 120 d; a) aerobic water-sediment system; b) anaerobic water-sediment system. Data from two different water/sediment systems with 2 samples, each (single data in Figure S13 and S14).

Also, under anaerobic conditions, the two water–sediment systems showed very consistent results. In accordance with the longer half-life of 6-PPDQ under these conditions (Table 2), 50%–60% AR of it remained after 100 days. Oxidative transformation is slowed down due to the limited availability of oxygen. Correspondingly, also less NER is formed in absolute amount (24%–28% AR), but this comprises a larger percentage of the 6-PPDQ removed (52%–66%) than under aerobic conditions. The extent of mineralization of 6-PPDQ to CO_2_ is very low (3%–6% AR) and corresponds to only 6%–12% of the 6-PPDQ removed. Under aerobic conditions, 30%–38% of the 6-PPDQ removed was mineralized.

The very limited mineralization under anaerobic conditions is also visible from the larger contribution of “unknown TPs” to the AR; under aerobic conditions, these compounds were, likely, more effectively degraded further, eventually into CO_2_ (Figure 2).

In addition to 6-PPDQ, numerous small peaks were observed by radio-LC, most of which could not be identified by LC-MS analysis. However, hydroxylation of both the quinone ring and the 1-methyl pentane chain was detected as well as oxidation of the 1-methyl pentane chain to carboxylic acid at two locations (Figure 1a; Table S15). This agrees with Shen et al.,^20^ who also reported the two hydroxylated 6-PPDQ isomers as well as TP211 (Figure 1b).

### 3.3 Soil

The biodegradation of [^14^C]-6PPDQ was finally studied in four soils maintained under aerobic and anaerobic conditions at 20 ± 2 °C, in the dark, over a period of 120 days. Namely, roadside soil is an important sink for TRWP and for 6-PPDQ.^26^

#### 3.3.1 Extent of Degradation and Kinetics

The biodegradation of 6-PPDQ was monitored by six samplings over 120 days under aerobic and seven samplings for the anaerobic experiments, combined with analysis by LSC, radio-HPLC, and LC-MS/MS of soil extracts. In the case of aerobic conditions, also CO_2_ formation was recorded. At the end of the experiments, the soils were also analyzed for NERs. Three kinetic models were applied to the data (Table 3, Table S21).

Under aerobic conditions, DT50 values of 6-PPDQ ranged from 27 to 141 d, with a median of 61 d, based on the SFO kinetic model for the soil extracts (Table 3). The DT50 values of 6-PPDQ in soil under aerobic conditions are in a similar range as those in aerobic sediment (30–40 d, Table 2). Labsoil B2 had the lowest pH (4.8) and the highest sand content (65%) of all four soils (Table S7). Also its biomass density was at the lower end of the four soils (Table S8). Taken together, these factors may explain why biodegradation of 6-PPDQ was slowest in this soil under aerobic conditions.

**Table 3:** Biodegradation Half-Life of [^14^C]-6-PPDQ Derived from the Single-First-Order Model (CAKE Software) in Soil under Aerobic and Anaerobic Conditions, According to OECD 307^a^

	Aerobic Soil Extracts		Anaerobic Soil Extracts	
	DT50 (d)	DT90 (d)	DT50 (d)	DT90 (d)
Brierlow	78	259	394	1310
South Witham	27.3	90.7	267	886
Labsoil B2	141	467	117	389
Speyer 5M	44.7	148	448	1049
				
median	61	203	330	967

	Aerobic Soil Extract + MAE + NER I		Anaerobic Soil Extract + MAE + NER I	
	DT50 (d)	DT90 (d)	DT50 (d)	DT90 (d)
Brierlow	131	436	615	2040
South Witham	34.7	115	447	1480
Labsoil B2	181	602	214	710
Speyer 5M	54.1	180	887	2950
				
median	92.6	308	531	1760

a Full data for this and two other kinetic models (DFOP: “double first-order in parallel”; FOMC: “first order multicompartment”) provided in Table S21. Data shown for the soil extracts (obtained by LSE) and for soil extracts plus extracts of microwave assisted extraction (MAE) and non-extracted residue fraction 1 (NER I). (see the Methods section for details).

Longer half-lives and larger differences are found under anaerobic conditions. Then, DT50 values in soil increased to 117 to 448 d, with a median of 330 d for the soil extracts with the SFO model (Table 3). Applying other kinetic models to the experimental data, DT50 values increased to >10 000 d for two of the soils (Table S21). Generally, the three kinetic models did not deliver exactly the same DT50 values but the relative standard deviation between the models usually remained ≤20%, as long as none of the DT50 values exceeded the experimental period by more than a factor of 2. However, the uncertainty in DT50 estimation increased as the need to extrapolate far beyond the experimental period of 120 d increased. One example is “Speyer 5M” under anaerobic conditions with a DT range from 448 d to >10 000 d for the three models applied (Table S21). Extending the duration of the degradation experiment to beyond 120 d would not have improved the DT50 estimation, as during extended experimental periods the microbial community composition could change too strongly and in a nonreproducible way.

The measured soil properties do not provide an explanation for the strongly differing DT50 values between the four soils. For example, biomass density at the end of the anaerobic experiment was low for “Speyer M5” (326 μg C per g of soil), but much lower for “Labsoil B2” (55 μg C per g of soil) (Table S8), although the latter exhibited a faster degradation of 6-PPDQ (Table 3). The second slowest “Brierlow” soil had a much higher biomass density (665 μg C per g of soil) after incubation under anaerobic conditions. It is reasonable to assume that differences in the microbial community are more decisive than the sheer amount of biomass in explaining the kinetics of biodegradation of 6-PPDQ under anaerobic conditions. The likely strong effect of the microbial community composition is also outlined by the kinetic data for “Labsoil B2”: it degrades 6-PPDQ the slowest of all four soils under aerobic conditions (DT50 141 d), while it became faster under anaerobic conditions (DT50 117 d). Soil flooding can strongly alter the composition and activity of the microbial community and its ability to degrade a substrate.

The DT50 values of 6-PPDQ in soil are below the 120 d threshold^22^ for three of the four tested soils under aerobic conditions, while one value exceeded that limit. Under anaerobic conditions, all but one DT50 values were above the threshold value (Table 3).

Although biodegradation half-lives are considered a compound property and used as such in chemicals regulation, these kinetic data outline the strong influence of environmental factors such as the soil properties, the redox conditions, and the composition of the microbial community on the biodegradation of chemicals in soil: these factors lead to a variation of the DT50 values of 6-PPDQ from 27 to 448 d for the eight setups tested here (Table 3) and to >10 000 d with other kinetic models (Table S21). It is for this diversity that it is recommended to perform biodegradation studies using several soils with different properties and under aerobic as well as anaerobic conditions. For surface soils, where most TRWPs carrying 6-PPDQ will be deposited, aerobic conditions will be the predominant ones for most of the time.

Biodegradation of 6-PPDQ in soil has been studied previously, although under different experimental conditions and using nonlabeled substrates. In one of the studies, a DT50 value of 14 d was derived for two soils under aerobic conditions.^20^ This was clearly shorter than the shortest DT50 determined in this study for 6-PPDQ without NER (27.3, Table 3). In another study, 6-PPDQ biodegradation was studied after adding CMTT to soil; although no DT50 values were reported, the data show that more than 50% of the 6-PPDQ was removed within 10 d under aerobic conditions.^21^ Both of these studies report faster biodegradation of 6-PPDQ than found in the simulation tests here. Differences in experimental design and conditions may be reasons for the faster degradation observed in the literature studies. For example, one of the studies used roadside soil which may have been preadapted to 6-PPD degradation.^20^ Whatever the reason for this difference may be, it can be concluded that the simulation test, which is often performed to generate data for the persistence assessment in regulation, is not overly optimistic.

In flooded soil, no degradation of 6-PPDQ from CMTT was reported over 60 d,^21^ which corresponds with this study insofar as DT50 values in soils were clearly longer under anaerobic (flooded) conditions, compared to aerobic ones (Table 3, Table S21).

Thanks to the [^14^C]-label, also NERs could be determined in the soil system, and NER I was included into the kinetic modeling.^15^ With NER I, the DT50 values ranged from 35–181 d (median 92 d) under aerobic conditions. Now, three of the soils were below or around the 120 d threshold, while one clearly exceeded it. Under anaerobic conditions the DT50 values for 6-PPDQ plus NER I incread to 214–887 d (median 330 d; Table 3). Using other kinetic models, DT50 values up to >10 000 were calculated for two of the soils (Table S21).

#### 3.3.2 Transformation Products

The temporal trend of aerobic 6-PPDQ degradation over 120 d (median of the four soils) is shown in Figure 3a. After 120 d, a median of 27% AR remained as 6-PPDQ, but the trend suggests that biotransformation of 6-PPDQ has not come to a halt but was still ongoing. The extent of mineralization over 120 d reached a median of 34% AR for the four soils, corresponding to around 50% of the 6-PPDQ that was degraded. A slightly lower percentage was transformed into NER (median 33% AR). The sum of transformation products detected by radio-HPLC was quite low and added up to a median of 2% AR.

**Figure 3:**
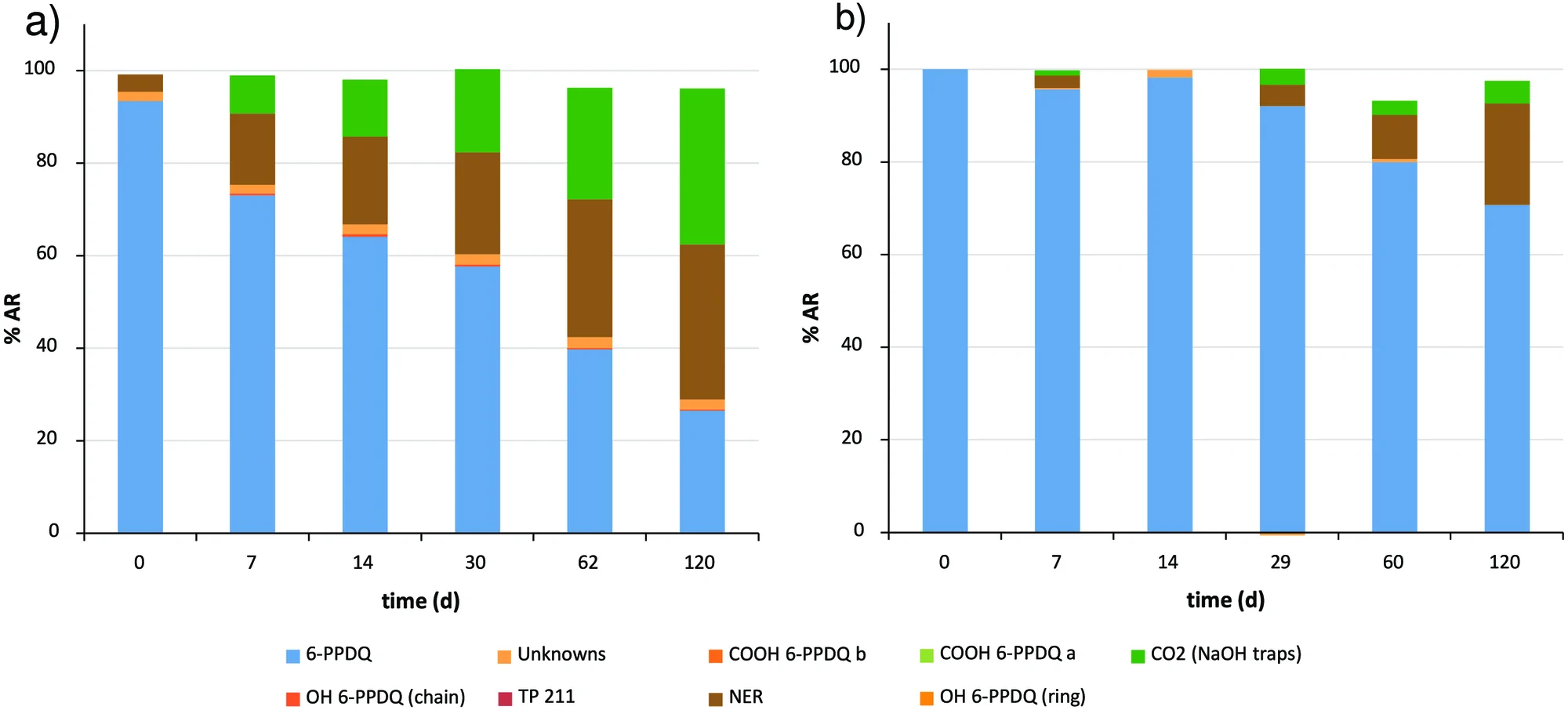
Median distribution of AR of [^14^C]-6-PPDQ over 120 d: (a) aerobic soil and (b) anaerobic soil. Data shown is from four soils and two parallel samples, each (single data in Figure S16 and S17).

In the flooded soil under anaerobic conditions, only 29% of 6-PPDQ was degraded within 120 d (Figure 3b). NERs comprise 22% AR (corresponding to 84% of the degraded 6-PPDQ), while mineralization accounted for 5% AR or 19% of the degraded 6-PPDQ only. This trend of increasing relative NER formation from 6-PPDQ with decreasing oxygen availability may be explained by the concept that an initial (oxidative) activation of 6-PPDQ allows for both subsequent mineralization as well as NER formation. When mineralization slows due to the limited availability of oxygen, NER formation can still take place, as it does not depend on the presence of molecular oxygen. The contribution of degradation products formed in the anaerobic phase and detected by radio-HPLC was negligible.

One study reported seven transformation products from 6-PPDQ biodegradation in soil, of which three agreed with the products reported here for the aqueous system (Figure 1b, Table S15).^20^ Three of their products (Q-TP345, Q-TP312, and Q-TP 151) appear to carry additional carbon or nitrogen atoms and may indicate coupling of biodegradation intermediates with other reactants in soil. None of the products reported had a retention time as short as that found here for the polar unknown transformation products.

On the basis of the signal intensities of the detected TPs, the authors noted that those accounted for only a few percent of the amount of 6-PPDQ degraded.^20^ This biodegradation study with [^14^C]-labeled 6-PPDQ not only substantiates but also explains this finding, as it shows that around 50% AR of the 6-PPDQ degraded under aerobic conditions were mineralized and close to 50% were transformed into NER in soil (Figure 3, Figure S19).

## 4 Conclusions

The simulation tests with [^14^C]-labeled 6-PPDQ show that this compound is biodegradable in water, water–sediment systems, and in soil. Under aerobic conditions, DT50 values were clearly below the regulatory threshold value for persistence, in water (40 d) and in water–sediment systems (120 d). In soil under aerobic conditions, the DT50 values of 6-PPDQ ranged from 27 to 141 d (median 61 d); for one of the four soils, the DT50 value exceeded the 120 d regulatory threshold. Considering NER I in soil DT50 values increased to 35–181 d, with one soil in the range of the 120 d-limit (131 d), while the fourth one showed a DT50 of 181 d.

Anaerobic conditions slow down the biodegradation of 6-PPDQ markedly in both water-sediment systems and soils. Under these conditions the DT50 values of 6-PPDQ including NER I increased in sediments to 140–202 d and in soils to 214–887 d (median 531 d). With another kinetic model the maximum DT50 value exceeded 10 000 d.

Under aerobic conditions, the extent of mineralization of 6-PPDQ was highest in water (34% AR) and the four soils (median 34% AR) and was only slightly lower in the water–sediment system (27% AR). In surface water, about 60% AR was detected as low-molecular-weight transformation products; ring-hydroxylated 6-PPDQ and 6-PPDQ carboxylic acid were the major transformation products identified, but the majority of products (50% AR) remained unidentified. In the water–sediment system and in soil, these transformation products were far less relevant. Instead, NER became quantitatively important, comprising 42% and 33% AR. With decreasing mineralization under anaerobic conditions, the relative importance of NER formation increased further: 67% of the 6-PPDQ degraded in the water–sediment system ended as NER and even 79% in soil.

While 6-PPDQ can be considered nonpersistent under aerobic conditions in water, water–sediment systems, and soil, the different simulation tests show the strong effect of environmental conditions (redox conditions, soil properties, microbial community) on both the rate of biodegradation of 6-PPDQ as well as the products formed. In the environment, the rate of biodegradation may be further modulated by the bioavailability of 6-PPDQ.

## Supplementary Material



## Notes

Additional details on the experimental procedures and analytical methods for experiments in water, water/sediment and soil systems; detailed results for experiments in water, water/sediment and soil systems, including recovery of applied radioactivity, kinetic results, chromatograms and mass spectrometric data (PDF)
