# Supplementary material for: Microbial Transformation of 6‑PPDQ in Surface Water, Water–Sediment, and Soil—Results from Simulation Tests with Radiolabeled Substrate
Source: ACS Environ Au. 2026 Aug 8;6(5):926–34. doi: 10.1021/acsenvironau.6c00125 (PMC13602331; doi:10.1021/acsenvironau.6c00125)
Supplement: Supplementary file 1 [file vg6c00125_si_001.pdf]

Supporting Information to

Microbial transformation of 6-PPDQ in surface water, water-sediment and soil – Results from simulation tests with radiolabeled substrate

Thorsten Reemtsma [a, b], Bettina Seiwert [a], Pete Yeomans [c], Rory Mumford [c], Sara Bennett [c]

a) Helmholtz Centre for Environmental Research – UFZ, Department of Environmental Analytical Chemistry, Permoserstrasse 15, 04318 Leipzig, Germany
b) University of Leipzig, Institute for Analytical Chemistry, Linnéstrasse 3, 04103 Leipzig, Germany
c) Smithers, Environmental Risk Sciences Division, 108 Woodfield Drive, Harrogate, HG1 4LS, United Kingdom

Table of Content

1. Details of the Experimental Procedures and Analytical Methods 5
 1.1 Test material and samples 5
 1.2 Degradation in surface water 8
  1.2.1 Experimental details 8
  1.2.2 Details of sample processing after experiment 10
 1.3 Degradation in water-sediment system 10
  1.3.1 Experimental details 10
  1.3.2 Details of sample processing after experiments 12
 1.4 Degradation in soil 13
  1.4.1 Experimental details 13
  1.4.2 Details of sample processing after experiments 16
 1.5 Analytical Procedures 16
  1.5.1 Radioactivity analysis 16
  1.5.2 Radio-LC 17
  1.5.3 LC-MS/MS 17
2. Details of Results 19
 2.1 Degradation in surface water 19
  2.1.1 Recovery of the applied radioactivity (AR) 19
  2.1.2 Kinetic Results 21
  2.1.3 Chromatograms 23
  2.1.4 Mass spectrometric data of the transformation products 25
 2.2 Water-sediment systems 36
  2.2.1 Recovery of the applied radioactivity (AR) 36
  2.2.2 Kinetic data 37
  2.2.3 Chromatograms 39
 2.3 Degradation in soil 41
  2.3.1 Recovery of applied radioactivity (AR) 41
  2.3.2 Kinetic results 43
  2.3.3 Chromatograms 48
 2.4 Comparison of the products of 6-PPDQ transformation 50
3. References: 51

List of Figures

Figure S 1 Scheme of the experimental setup for test in surface water 9
Figure S 2 Test setup for the degradation experiments in water-sediment systems under aerobic conditions 11
Figure S 3 Test setup for the degradation experiments in water-sediment systems under anaerobic conditions 12
Figure S 4 Experimental setup for the test with soil under aerobic conditions 15
Figure S 5 Experimental setup for the test in soil under anaerobic conditions.................................... 15
Figure S 6 Distribution of applied radioactivity of [14C]-6-PPDQ (1.5 µg/L) during biodegradation in water 22
Figure S 7 Distribution of applied radioactivity of [14C]-6-PPDQ (15 µg/L) during biodegradation in water 23
Figure S 8 Radio-chromatograms obtained from samples of 6-PPDQ biodegradation in water 23
Figure S 9: LC-MS chromatograms and spectra of suspected OH-6-PPDQ, Ring 27
Figure S 10: LC-MS chromatograms and spectra of suspected OH-6-PPDQ, side-chain 30
Figure S 11: LC-MS chromatograms and spectra of suspected COOH-6-PPDQ, side-chain 32
Figure S 12: LC-MS chromatograms and spectra of TP 211.. 34
Figure S 13 Distribution of applied radioactivity of [14C]-6-PPDQ during biodegradation in two water-sediment systems under aerobic conditions 38
Figure S 14 Distribution of applied radioactivity of [14C]-6-PPDQ during biodegradation in two water-sediment systems under anaerobic conditions 38
Figure S 15 Radio-chromatograms obtained from samples of 6-PPDQ biodegradation in water-sediment system.. 39
Figure S 16 Distribution of applied radioactivity of [14C]-6-PPDQ during biodegradation in four different soils under aerobic conditions 46
Figure S 17 Distribution of applied radioactivity of [14C]-6-PPDQ during biodegradation in four different soils under anaerobic conditions. AR at day of flooding set to 100%; time = time after flooding... 47
Figure S 18 Radio-chromatograms obtained from samples of 6-PPDQ biodegradation in soil............ 48
Figure S 19 Percentage distribution of the products of 6-PPDQ (as % AR of 6-PPDQ degraded). 50

List of Tables

Table S 1: Details for Reference Standards 5
Table S 2: Origin and properties of the surface water sample 6
Table S 3 Sampling location and date of the water-sediment samples 6
Table S 4 Properties of the sediment.. 6
Table S 5 Properties of the overlying water.. 7
Table S 6 Sampling locations and dates of sampling of the soil samples 7
Table S 7 Characteristics of the soils used for testing.. 7
Table S 8 Microbial biomass in the soils (µg C per g of soil) 7
Table S 9 Time under aerobic conditions (before flooding) for the degradation experiments in soil under anaerobic conditions.. 14
Table S 10: Parameters for the mass spectrometric analysis 18
Table S 11 Distribution and recovery of radioactivity of [14C]-6-PPDQ (1.5 µg/L) in surface water..... 19
Table S 12 Distribution and recovery of radioactivity of [14C]-6-PPDQ (15 µg/L) in surface water...... 20
Table S 13 Distribution and recovery of radioactivity of [14C]-6-PPDQ (15 µg/L, sterile) in surface water 21
Table S 14 Results of the Single First Order (SFO) kinetic modeling of biodegradation of [14C]-6-PPDQ in surface water 21
Table S 15 Mass spectrometric data of 6-PPDQ and its transformation products detected by LC-MS/MS, their occurrence in different experiments and reports in literature. 25
Table S 16 Recovery of applied radioactivity (% AR) in the biodegradation of [14C]-6-PPDQ in water-sediment systems 36
Table S 17 Kinetic data of [^{14}C]-6-PPDQ biodegradation in the two water sediment systems under aerobic and anaerobic conditions 37
Table S 18 The recovery and distribution of applied radioactivity (AR) in the different soils 41
Table S 19: Percent recovery of applied radioactivity including NER I-III from additional extractions of soil treated with [14C]-6PPD-quinone and maintained under aerobic conditions at the end of the experiment. 42
Table S 20 Percent recovery of applied radioactivity including NER I-III from additional extractions of soil treated with [14C]-6PPD-quinone and maintained under anaerobic conditions at the end of the experiment. 42
Table S 21 Kinetic data for [14C]-6-PPDQ in soils under aerobic and anaerobic conditions applying different kinetic models... 43

1. Details of the Experimental Procedures and Analytical Methods

1.1 Test material and samples

[14C]-6PPDQ was supplied by Pharmaron UK Limited and used at a radiochemical purity of >95% and a specific activity of 7 – 8 MBq/mg. Reagents and solvents were analytical grade (or a suitable alternative) and were obtained from commercial suppliers.

[14C]-6PPD-quinone

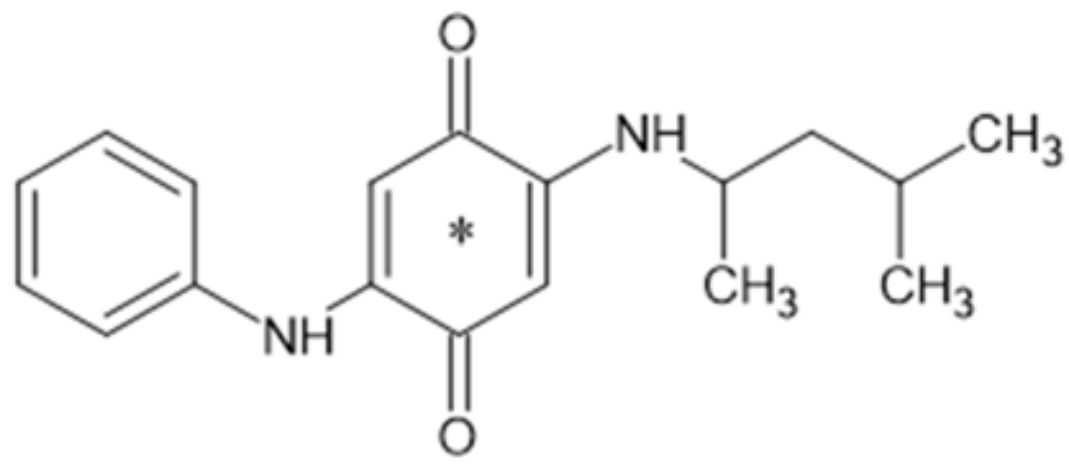

* indicates position of radiolabel

Table S 1: Details for Reference Standards

ID	Structure	Monoisotopic Mass (Da)	Theoretical Molecular Ion (*m/z*)
6-PPDQ		298.1681	299.1754 $[M+H]^+$
4-Aminophenol		109.0528	110.0600 $[M+H]^+$
Aniline		93.0578	94.0651 $[M+H]^+$
4-Hydroxy diphenylamine		185.0841	186.0913 $[M+H]^+$

Surface water

Table S 2: Origin and properties of the surface water sample

Sampling location and data	Oak Beck, Penny Pot Lane, Harrogate, UK. 11 August 2023		
TOC (mg/L)	15.3	TSS (mg/L)	19
BOD5 (mg/L)	2.65	Alkalinity (CaCO3 mg/L)	139
NH4-N	0.04	NO2-N (mg/L)	0.02
PO4-P	0.09	NO3-N (mg/L)	1.43

Water-sediment

Table S 3 Sampling location and date of the water-sediment samples

	Calwich Abbey	Middle Pond
Location	Calwich Abbey, Calwich, Staffordshire, UK	Middle pond, Lumsdale, Derbyshire, England
GPS coordinates	52°59'6.4" N, 1°48'38.3" W	53°8'39.2" N, 1°32'0.8" W
Site characteristics	Lake fed by stream from the River Dove and fringed with woodland and ley grassland	Shallow mill pond, located in wooded valley
Sampling date	24 July 2023	25 July 2023

Table S 4 Properties of the sediment

Sediment	pH ($CaCl_2$)	OC (%)	Sand[1] (%)	Silt[1] (%)	Clay[1] (%)	Classification[1]	Biomass (µg C/g Sediment)
Calwich Abbey	7.6	4.1	15	80	5	Silt	2748
Middle Pond	7.3	2.9	79	14	7	Loamy sand	231

1 USDA Particle Size Distribution and Classification
OC = organic carbon

Table S 5 Properties of the overlying water

Water	pH (at sampling)	TOC (ppm)	Suspended solids (mg/L)
Calwich Abbey	8.31	4.900	23
Middle Pond	7.41	4.876	9

TOC = total organic carbon

Soil

Table S 6 Sampling locations and dates of sampling of the soil samples

	Brierlow	South Witham	Labsoil B2	Speyer 5M
Location	Brierlow, Derbyshire, UK	South Witham. Lincolnshire, UK	Ingleby, Derbyshire, UK	Mechtersheim, Rheinland-Pfalz, Germany
coordinates	53°13’9.4” N, 1°51’32.4” W	52°45’3.7” N, 0°38’7.4” W	52°50’19.8” N, 1°29’ 13.9” W	
supplier	LRA Labsoil	LRA Labsoil	LRA Labsoil	LUFA Speyer, batch F5M 31/23
Sampling date	31 May 2023*	03 July 2023*	15 August 2023	03 August 2023
Land use	Grassland field	Vegetation cover	Permanent pasture field	meadow
USDA soil taxonomy	Inceptisol	Inceptisol	Entisol	Entisol

*) Both stored in a soil nursery and sampled from the nursery on 16 August 2023.

Table S 7 Characteristics of the soils used for testing

	Brierlow	South Witham	Labsoil B2	Speyer 5M
pH (CaCl2)	5.5	7.7	4.8	7.5
Organic carbon (%)	3.6	3.1	1.1	1.0
Sand[a] (%)	28	40	65	13
Silt[a] (%)	52	19	27	32
Clay[a] (%)	20	41	8	56
Classification[a]	Silt loam	Clay	Sandy loam	Sandy loam

[a]) USDA Particle Size Distribution and Classification

Table S 8 Microbial biomass in the soils (µg C per g of soil)

	Brierlow	South Witham	Labsoil B2	Speyer 5M
Prior to study	539	888	218	238

	aerobic			
At treatment	443	713	167	187
End of study	542	711	204	145
	anaerobic			
At treatment	415	668	172	209
After incubation	665	690	55	326

1.2 Degradation in surface water

1.2.1 Experimental details

Application Solution

The low-test concentration application solutions (0.0017 mg/mL; AS1, AS4) were prepared by transferring an aliquot (3 µL) of SS1 into a vial and diluting with acetonitrile (3.042 mL), followed by sonication to aid dissolution.

To prepare the high-test concentration application solutions (0.034 mg/mL; AS2, AS5), an aliquot (42 µL) of SS1 was transferred into a vial, the solvent evaporated and resuspended with acetonitrile (4.68 mL), followed by sonication to aid dissolution.

For the determination of the exact application rate and to check homogeneity, triplicate aliquots (90 µL) of AS1, AS2, AS4 and AS5, respectively, were removed pre- and post-application (6 samples in total) and diluted into 10 mL of acetonitrile. Triplicate 250 µL aliquots were transferred directly to LSC vials containing ca 5 mL of scintillant for quantification by LSC.

The sodium benzoate treatment solution (0.0013 mg/mL; AS3) was prepared by reducing benzoic acid [ring 14C] sodium salt (126 µL) supplied in ethanol (0.246 mg/mL, 4.41 MBq/mL) to dryness and re-dissolving in Milli-Q water (26 mL). The application rate and homogeneity of the treatment solution were determined by transferring triplicate aliquots (100 µL) of the treatment solution in 10 mL of water, pre- and post-application (6 samples in total), and taking triplicate 250 µL aliquots directly to LSC vials containing ca 5 mL of scintillant for quantification by LSC.

Test Substance Application

Natural surface water samples were treated with the appropriate volume of application solution (91 µL of AS1, or 99 µL of AS4, for low concentration, 92 µL of AS2 or AS5 for high concentration and sterile vessels) using a positive displacement pipette. The samples were swirled before being placed back onto the incubation rig. The measured concentration of [14C]-6-PPDQ in the low test concentration group was determined as 1.51 to 1.52 µg/L and 15.1 to 15.2 µg/L in the high test concentration group. The nominal concentrations for the study were chosen based on the low water solubility of [14C] 6-PPDQ.

Two reference samples of ca 100 mL natural surface water (incubation group D) were treated with 800 µL of sodium benzoate treatment solution (0.0125 mg/mL) and 92 µL of acetonitrile using a positive displacement pipette. After treatment, the reference samples were swirled and placed back onto the incubation rig. The measured concentration of sodium benzoate per vessel was determined as 10.0 µg/L.

Incubation Conditions

Eighteen vessels of natural water were prepared per concentration, ten for set sampling times plus two spares. Two vessels were prepared for the sodium benzoate controls and two vessels were

prepared as sterilised units, which were treated at the high concentration. Additionally, two control vessels were prepared which were used to determine incubation parameters. Four additional vessels of natural water were prepared on a second occasion, for use as intermediate applications.

The vessels were maintained under experimental conditions for up 2 days at 20 ± 2°C in the dark prior to test substance application. During the pre-incubation phase the safety trap after the vessel was filled with water to check for air-flow through the vessel. The water was emptied and trapping reagents were added to the other traps prior to test substance application.

After treatment, moistened air was drawn through the vessels and, after test substance application through a series of traps. The first trap was an empty vessel used as a security trap, the second and third traps (named Trap 1 and Trap 2 in schematic diagram overleaf) contained sodium hydroxide solution (2M) to collect liberated carbon dioxide.

Time Zero samples were analysed immediately following application. All remaining samples were maintained in the dark at 20 ± 2°C.

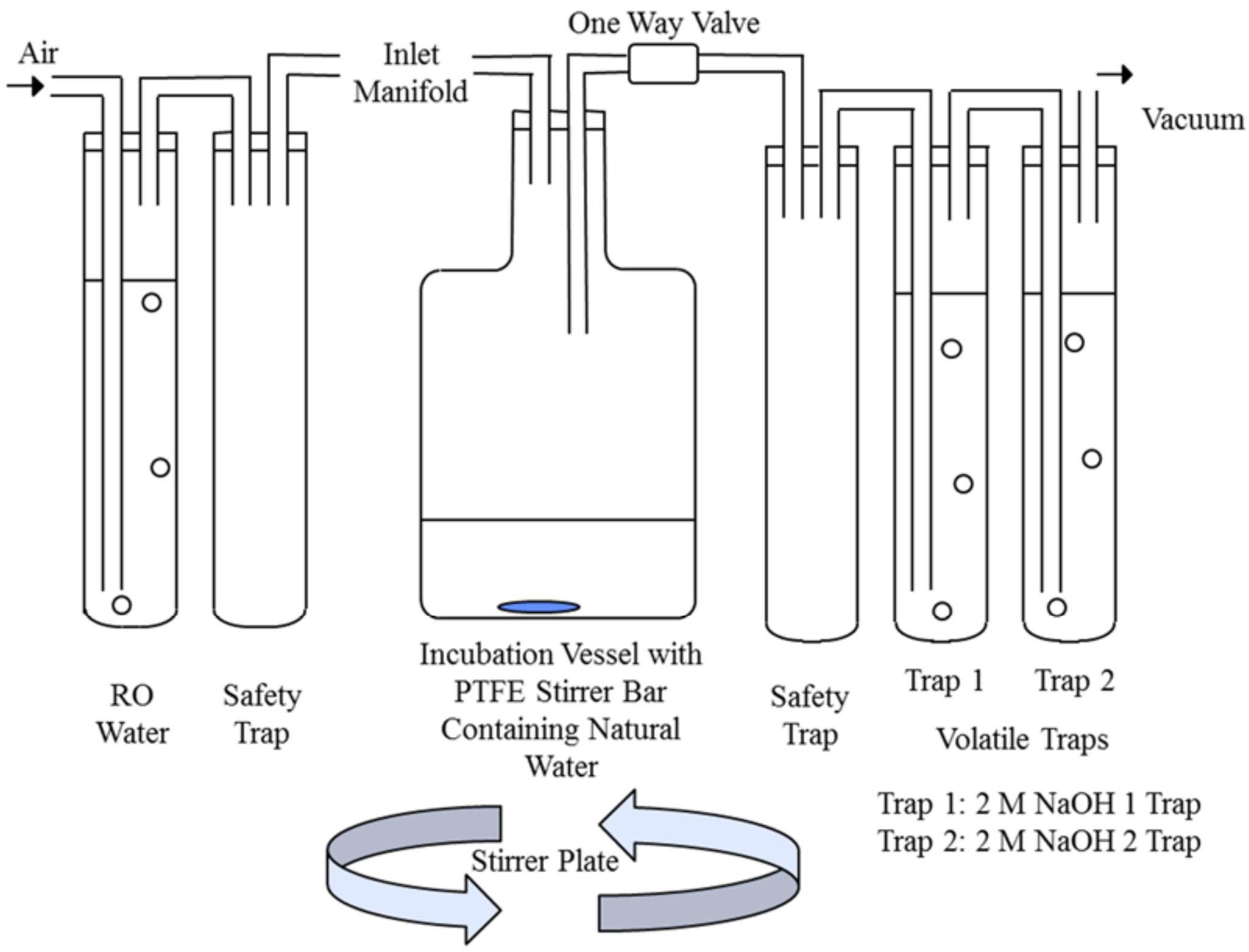

Figure S 1 Scheme of the experimental setup for test in surface water

Sampling Intervals

At intervals of 0 (immediately after treatment) 7, 14, 21, 28, 45 and 60 days after treatment (DAT) for dose group A and B, duplicate vessels treated with radiolabeled test substance were analysed at each time point. Additional intervals of 3 and 10 days were dispensed and treated with radiolabeled test substance on a separate occasion.

Oxygen levels were high throughout the study in the water (> 9 mg/L), except with the 60 day being 8.9 mg/L, and therefore the aerobic nature was confirmed. Water pH remained relatively constant (7.9 – 8.3) throughout the study.

1.2.2 Details of sample processing after experiment

At sampling, the contents of the incubation vessel (minus the associated magnetic stirring bar) were transferred to a pre-weighed vessel (Extract 1), The incubation vessel and associated magnetic stirrer were washed with acetonitrile (5 mL) twice, acetonitrile:water (1:1,v/v) and water (5 mL) and shaken before combining with Extract 1. All Extract 1 samples were quantified by LSC. A 50 mL weighed subsample of Extract 1 was concentrated under reduced pressure and centrifugation (or stream of nitrogen gas) and centrifugation until the final volume was ca 0.5 mL. the vessel was washed with ca 1 mL of acetonitrile, followed by acetonitrile: water (1:1), and water and combined into a pre-weighed glass vessel (Extract 2). All extracted and concentrated samples were quantified by LSC and HPLC analysis.

Volatile Traps: Radioactivity in the trapping solutions was quantified by LSC when the vessels to which they were attached were removed for analysis. The traps attached to the sodium benzoate vessels were analysed at every 4 days, up to 14 days, then every 14 days after. Fresh traps were added to those vessels continuing incubation.

Storage: All samples were analysed using reverse phase HPLC with fraction collection as soon as possible after concentration. Samples were stored frozen and in the dark between analyses.

1.3 Degradation in water-sediment system

1.3.1 Experimental details

The following groups of experiments were performed:

- A (DG A): Calwich Abbey, aerobic
- B (DB B): Middle Pond, aerobic
- C (DG C): Calwich Abbey, anaerobic
- D (DG D): Middle Pond, anaerobic

Test Substance Application: Due to the nature of the test substance, no target application rate could be selected from normal usage. Instead, the application rate of 200 kBq (ca 29 µg) per vessel was used as a target as it was shown in preliminary testing to allow both recovery of the applied radioactivity and analysis of the overlying water and sediment extracts.

The exact concentration of [14C]-6PPD-quinone in the application solution was determined by taking triplicate aliquots (85 or 84 µL), pre- and post-application, and diluting to 10 mL in volumetric flasks. Aliquots (100 µL) of the dilutions were taken directly into scintillant (ca 5 mL) and counted by LSC.

Aliquots of Application Solution 1 (84 µL) were dispensed dropwise over the water surface using a positive displacement pipette and the vessels gently swirled. Each vessel was treated with 29.3 µg of [14C]-6PPD-quinone.

Incubation Conditions: 0 DAT samples were analysed immediately following application. All remaining samples were maintained in the dark at 20 ± 2°C.

For aerobic groups, moistened air was drawn through the vessels and, after test substance application, through a series of traps. The first trap was an empty vessel used as a security trap. The second and third traps contained 2M sodium hydroxide solution to collect liberated carbon dioxide.

For anaerobic groups, moistened nitrogen was forced through the vessels and, after test substance application, through a series of traps. The first trap was an empty vessel used as a security trap. The second and third traps contained 2M sodium hydroxide solution to collect liberated carbon dioxide.

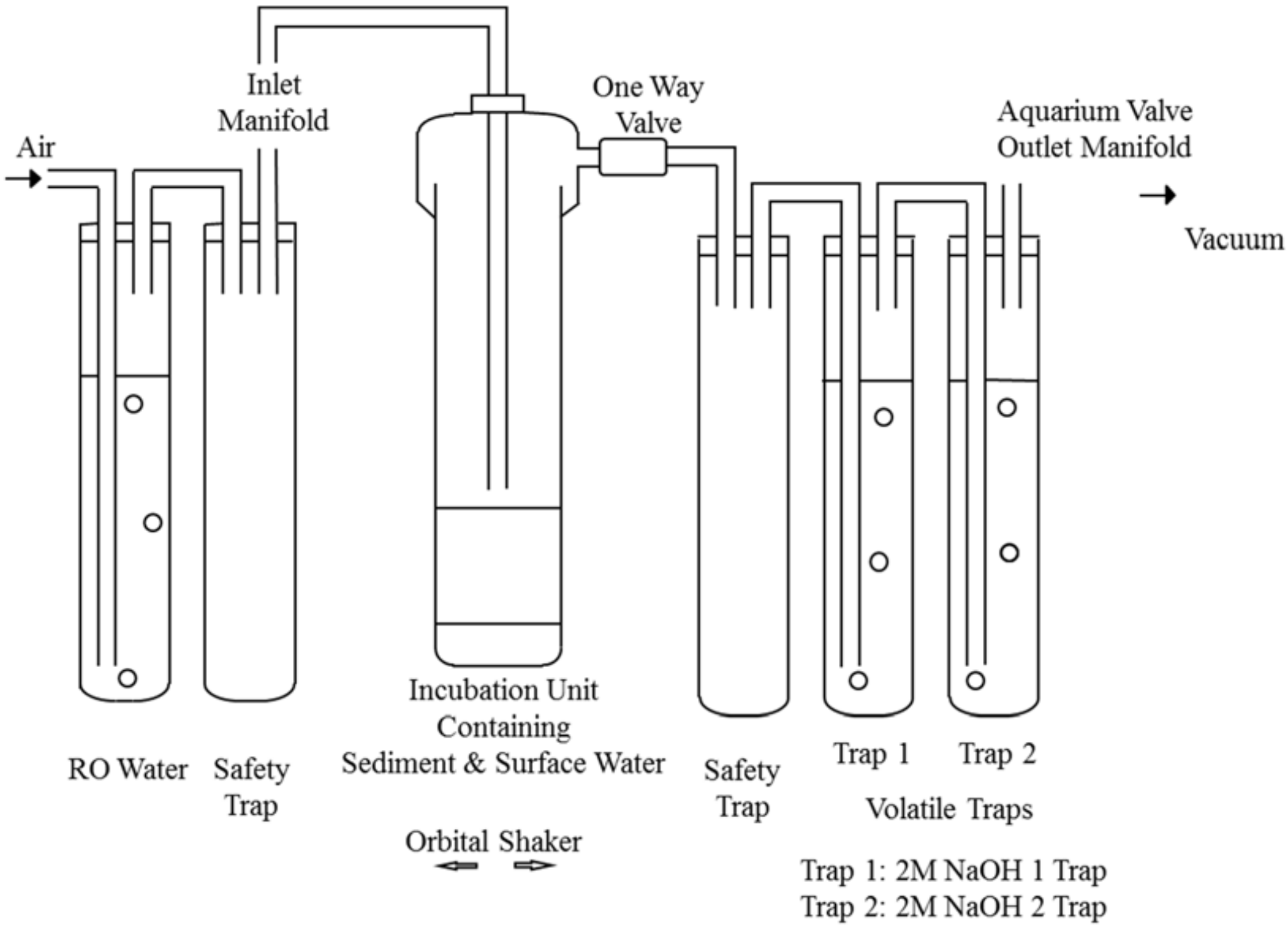

Figure S 2 Test setup for the degradation experiments in water-sediment systems under aerobic conditions

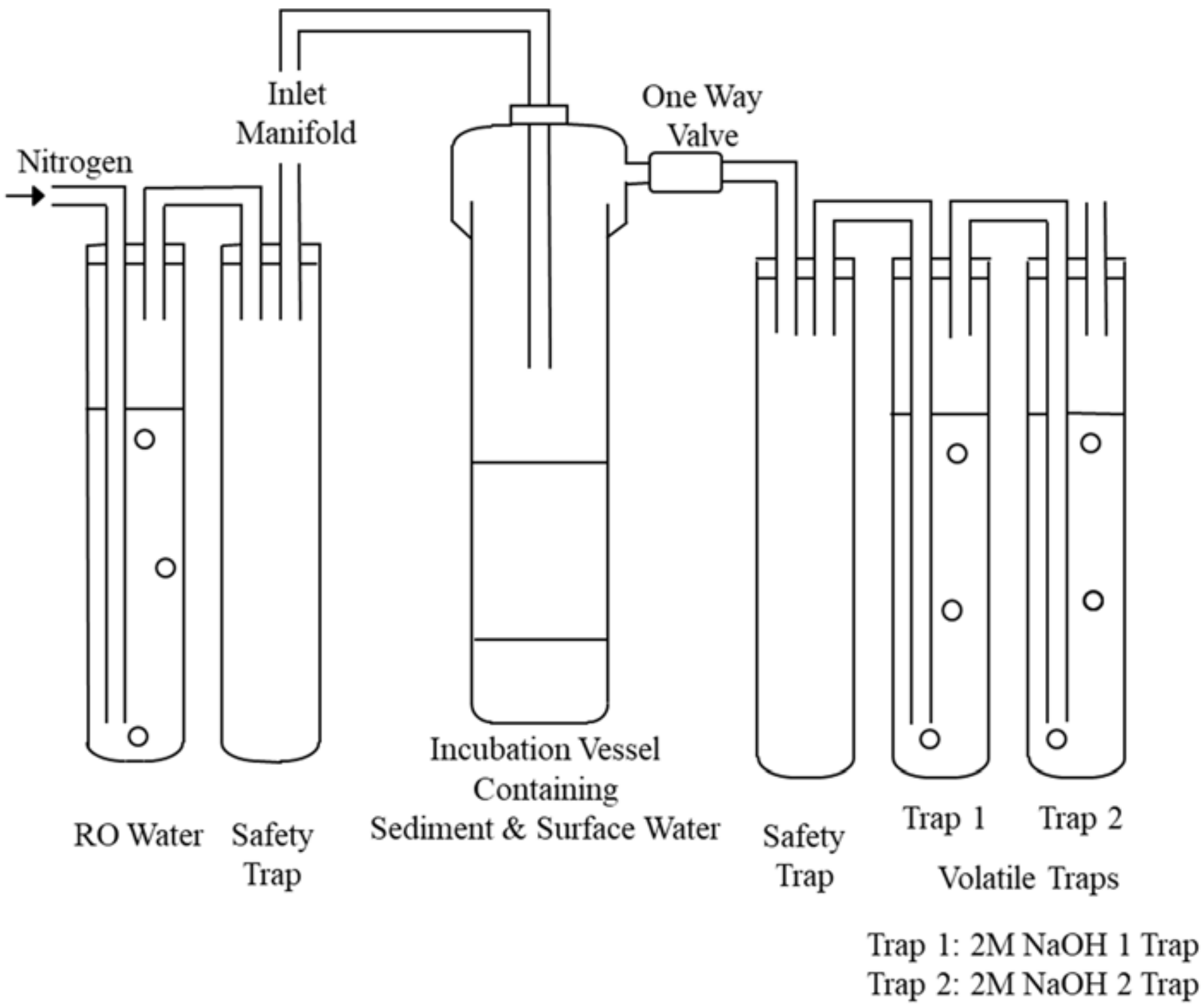

Figure S 3 Test setup for the degradation experiments in water-sediment systems under anaerobic conditions

Water-sediment samples were maintained under experimental conditions for 27 days (aerobic) or 26 days (anaerobic) at 20 ± 2°C in the dark, prior to test substance application. During the preincubation phase, the safety trap after the unit was filled with water to check for air-flow through the unit. The water was emptied and trapping reagents were added to the other traps prior to test substance application.

Water in the aerobic samples was maintained at oxygen concentrations > 7 mg/L throughout the incubation. Water in the anaerobic samples was maintained at oxygen concentrations < 2 mg/L throughout the incubation.

Aerobic sediment redox potentials were generally in the range 186 to -93 mV (Calwich Abbey) and 262 to -38 mV (Middle Pond) and were deemed to be anaerobic throughout the incubation period. Sediments for the anaerobic treatment groups were 258 to -93 mV (Calwich Abbey) and 270 to 187 mV (Middle Pond) on the hydrogen scale. Assessed as a function of pH, they were deemed to be anaerobic throughout the incubation period.

Sampling Intervals: At intervals of 0 (immediately after treatment), 7, 14, 29, 61 and 100 DAT, duplicate samples from each water-sediment system treated with radiolabeled test substance were removed for analysis.

1.3.2 Details of sample processing after experiments

Overlying Water: The overlying water was decanted into a vessel containing acetonitrile (150 mL). This was mixed and quantified by taking duplicate aliquots (ca 1 mL) for LSC. A subsample (ca 150 mL) was concentrated by either centrifugal evaporation to ca 5 mL or rotary evaporation to ca 2 mL and reconstituted using acetonitrile. The proportion of acetonitrile required in the final extract increased over the duration of the study to ca 4:1 (v:v).

Liquid Solid Extraction (LSE): The sediment was transferred to pot with the aid of acetonitrile (100 mL). The mixture was shaken for 20 minutes, centrifuged for 10 minutes (3500 g) and the supernatant decanted off. The extraction was repeated twice and the supernatants combined. The acetonitrile for extraction was also used to wash the incubation vessel. The extract was quantified by taking duplicate aliquots (ca 500 µL) into scintillant for LSC.

Analysis of Trapping Solutions: Radioactivity in the trapping solutions was quantified by LSC when the vessels to which they were attached were removed for analysis or every 30 to 40 days.

NER characterization

NER characterization adhered to the respective ECHA guideline (2023) and was continued to step 2. Microwave Assisted Extraction (MAE): Subsamples (ca 10 g) of the dried soil residues of selected 100 DAT samples from each treatment group were subjected to microwave assisted extraction. Acetonitrile : Milli-Q water (1:1 v:v, ca 20 mL) was added to the residue and maintained at 120°C for 15 minutes. The samples were allowed to cool, transferred to a pot and centrifuged for 10 minutes at 3000 g. The supernatants were removed, and the residues shaken with acetonitrile (ca 20 mL) and the centrifuging repeated. The two supernatants were combined and quantified by LSC. The residues were dried and combusted to quantify.

NER Characterisation – Silylation: Subsamples of the residues of harsh extracted soils were stirred (3 hours) with chloroform (ca 60 mL), trimethylchlorosilane (ca 30 mL) and sodium hydroxide (ca 3 g). Additional trimethylchlorosilane (ca 20 mL) and sodium hydroxide (ca 3 g) were added and the mixture was stirred overnight.

The mixture was transferred to a plastic pot, the stirrer bar and any remaining sodium hydroxide pellets were removed and, after centrifuging (ca 3000 g for ca 10 minutes), the chloroform layer was removed. The pot contents were washed three times with acetone (ca 20 mL) and three times with chloroform (ca 20 mL), centrifuging each time as above and combining with the chloroform layer prior to LSC. The residue was allowed to dry before grinding and combusting triplicate ca 0.2 g aliquots.

NER Characterisation - Amino Acid Extraction: Subsamples of the residues of harsh extracted soils were reflux extracted overnight with 6M HCl. After cooling, the mixture was transferred to a plastic pot, and, after centrifuging (ca 3000 g for ca 10 minutes), the acid layer was removed. The residue was washed twice with Milli-Q water (20 mL) centrifuging each time as above and combining with the acid layer prior to LSC.

1.4 Degradation in soil

1.4.1 Experimental details

The following groups of experiments were performed:

- A: Brierlow, aerobic
- B: South Witham, aerobic
- C: Labsoil B2, aerobic
- D: Speyer 5M, aerobic
- E: Brierlow, anaerobic
- F: South Witham, anaerobic
- G: Labsoil B2, anaerobic
- H: Speyer 5M, anaerobic

Incubation Conditions: 0 DAT samples were analysed immediately following application. All remaining samples were maintained in the dark at 20 ± 2°C.

For aerobic groups, moistened air was drawn through the vessels and, after test substance application, through a series of traps. The first trap was an empty vessel used as a security trap. The second and third traps contained 2M sodium hydroxide solution to collect liberated carbon dioxide.

For anaerobic groups after flooding, moistened nitrogen was forced through the vessels and through a series of traps. The first trap was an empty vessel used as a security trap. The second and third traps contained 2M sodium hydroxide solution to collect liberated carbon dioxide.

Soil samples were maintained under experimental conditions for 5 days (aerobic) or 5 to 7 days (anaerobic) at 20 ± 2°C in the dark, prior to test substance application. During the pre-incubation phase, the safety trap after the unit was filled with water to check for air-flow through the unit. The water was emptied and trapping reagents were added to the other traps prior to test substance application.

Sampling Intervals

Duplicate aerobic samples were collected for analysis at intervals of 0 (immediately after treatment), 7, 14, 30, 62 and 120 DAT (days after treatment).

Duplicate anaerobic samples were collected for analysis immediately after treatment, on the day of flooding and at 7, 14, 29, 60 and 120 DAF (days after flooding).

Table S 9 Time under aerobic conditions (before flooding) for the degradation experiments in soil under anaerobic conditions

Soil	Time Between Treatment and Flooding for Anaerobic Samples (days)
Brierlow	30
South Witham	16
Labsoil B2	30
Speyer 5M	21

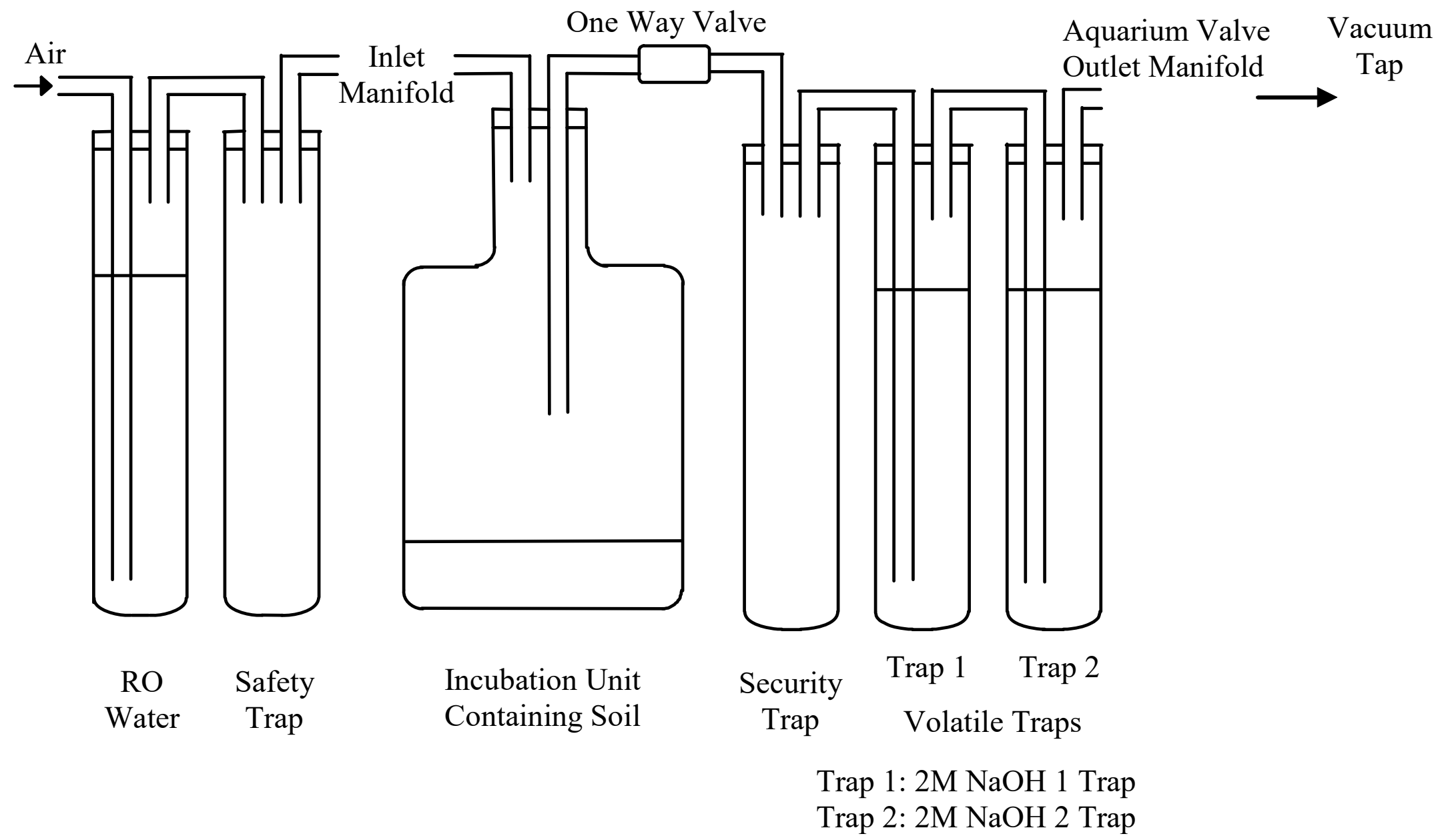

Figure S 4 Experimental setup for the test with soil under aerobic conditions

For aerobic groups, moistened air was drawn through the vessels and, after test substance application, through a series of traps. The first trap was an empty vessel used as a security trap. The second and third traps contained 2M sodium hydroxide solution to collect liberated carbon dioxide.

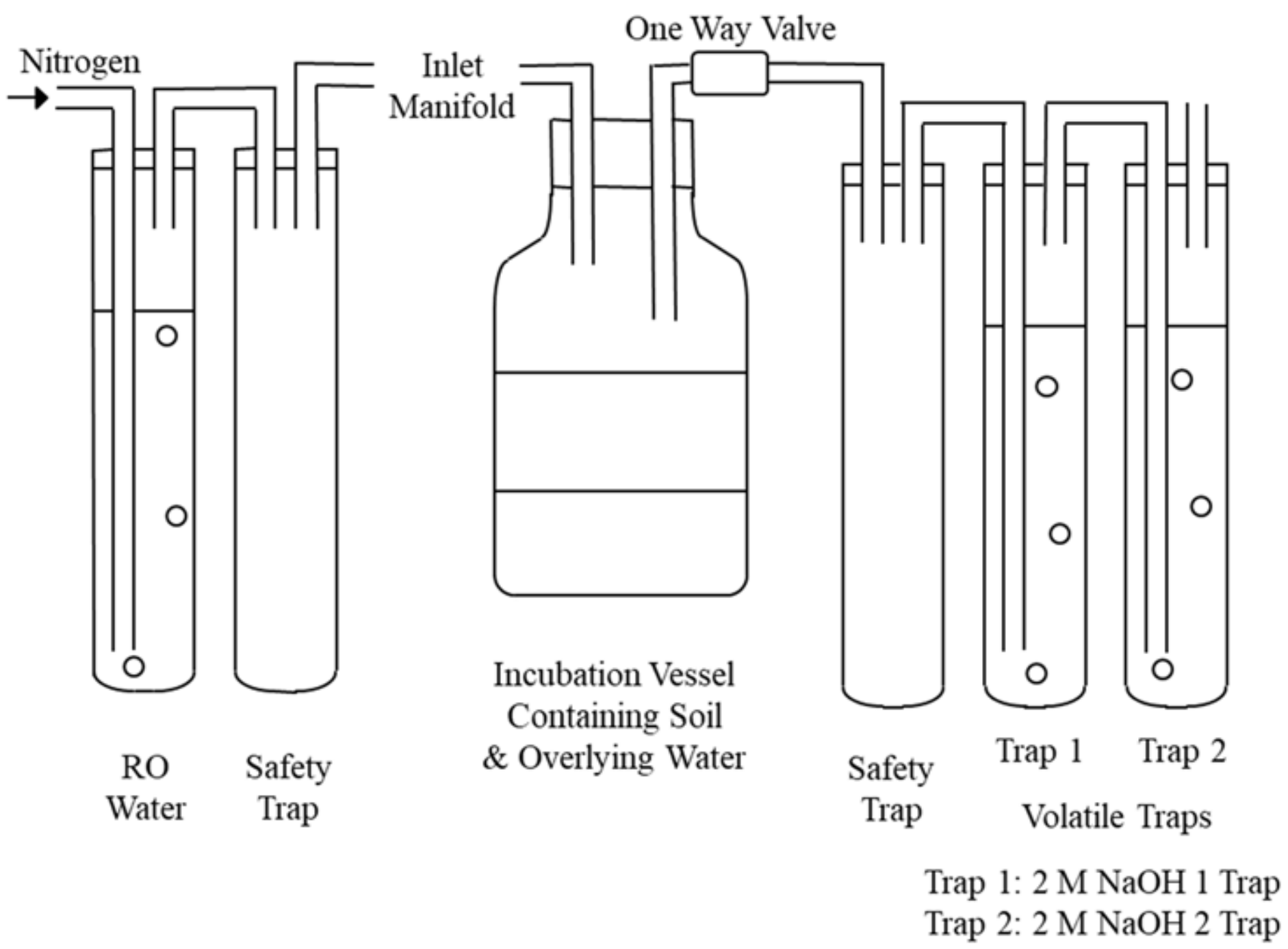

Figure S 5 Experimental setup for the test in soil under anaerobic conditions

For anaerobic groups after flooding, moistened nitrogen was forced through the vessels and through a series of traps as described for the aerobic groups.

Duplicate aerobic samples were collected for analysis at intervals of 0 (immediately after treatment), 7, 14, 30, 62 and 120 DAT (days after treatment). Duplicate anaerobic samples were collected for

analysis immediately after treatment, on the day of flooding and at 7, 14, 29, 60 and 120 DAF (days after flooding).

1.4.2 Details of sample processing after experiments

After collection soils were extracted twice with acetonitrile: Milli-Q water (1:1 v/v) or acetonitrile followed by acetonitrile: Milli-Q water (1:1 v/v) for flooded vessels. All samples were then extracted three times with acetonitrile.

Selected 120 DAT/DAF samples from each treatment group were subjected to microwave assisted extraction (MAE) followed by characterisation of the non-extracted residues (NER).

Extracts were quantified by LSC, combined, concentrated and analysed by HPLC. Unextracted radioactivity was determined following combustion of the extracted, air-dried soil.

1.5 Analytical Procedures

1.5.1 Radioactivity analysis

Radioactivity in all sample types was measured by liquid scintillation counting (LSC) using a Packard Tricarb model 1900TR or 2900TR liquid scintillation counter.

The radioactivity in application solutions was determined by taking triplicate aliquots by volume into volumetric flasks and filling to the mark. Triplicate aliquots from these dilutions (100 µL or 250 µL) were mixed with scintillant (ca 5 mL) prior to LSC quantification. Radioactivity in sample extracts and traps was determined by measuring the total sample weight and taking duplicate weighed aliquots of these samples directly into scintillant (ca 5 mL) prior to LSC quantification.

Following extraction, air-dried soil and sediment samples were ground and sub-sampled (ca 0.2 g) in triplicate prior to combustion in oxygen using a Hidex 600 OX oxidiser. The combusted products were absorbed in Hidex Radiocarbon and the radioactivity absorbed was determined by LSC. Carbon-14 standards were combusted at the beginning and at regular intervals throughout the day to check the carry-over between samples and to determine the efficiency of the combustion process. Combustion and trapping efficiencies were 99 ± 4% and therefore no correction factors were applied to the reported data.

Radioactivity was measured using a Packard Tricarb model 1900TR or 2900TR liquid scintillation counter with facilities for computing quench corrected disintegrations per minute (dpm).

Efficiency correlation curves were routinely checked by the use of quenched standards which were commercially available (Perkin Elmer Life and Analytical Sciences) or prepared at Smithers and quenched over the range observed in test samples. The limit of detection (LOD) was taken as 1.5 times the background radioactivity determined by counting samples with no radioactivity in the same batch as the samples.

Determination of Proportion of Residues Deemed Equivalent to 6PPD-quinone in sediments and soils:

For the selected samples for harsh extractions, the sum of the concentrated microwave extract and the pellet derived from it was used to calculate a percentage of the unextracted residue which could be considered equivalent to 6-PPDQ.

For the selected samples for NER extractions, the silylation extract (NER type I) was used to calculate a percentage of the unextracted residue which could be considered equivalent to 6-PPDQ for kinetic analysis.

These equivalences were summed to calculate a percentage of unextracted residue (after initial extractions) that may be considered equivalent to 6-PPDQ and used to modify the 6-PPDQ recovery for calculation of degradation rates.

1.5.2 Radio-LC

Chromatographic analysis was performed using reverse phase HPLC with a Restek Ultra Biphenyl column (250 x 4.6 µm, 5um) with radioactivity and UV detection

The following HPLC method was used:

Column:	Restek Ultra Biphenyl 250 x 4.6 mm, 5 µm		
Guard Cartridge	Phenomenex phenyl 4 x 3 mm		
Gradient:	A binary gradient was used consisting of:		
	Solvent A:	0.1% Formic acid in water	
	Solvent B:	0.1% Formic Acid in Methanol	
	<u>Time(min)</u>	<u>% A</u>	<u>% B</u>
	0	80	20
	5	80	20
	50	0	100
	55	0	100
	56	80	20
	60	80	20
Flow rate:	1 mL/min		
Temperature:	40°C		

Radiochromatograms were quantified using Laura software (LabLogic Systems Limited).

1.5.3 LC-MS/MS

LC-MS/MS analysis was conducted using an Orbitrap Eclipse Tribrid mass spectrometer (Thermo Fisher Scientific Inc) with Xcalibur software. The instrument source and optics were tuned for the best transmission of 6-PPDQ and reference standards.

The following equipment was used:

Mass Spectrometer:	Thermo Scientific Orbitrap Eclipse
HPLC:	Thermo Scientific Vanquish Horizon consisting of,
	Vanquish Binary Pump H (U)HPLC Pump
	Vanquish Split Sample HT Autosampler
	Vanquish Column Compartment H
	Vanquish Diode Array Detector FG-1
Radiodetector:	LabLogic β-RAM Model 5C radio detector
Data Capture:	Thermo-Scientific Xcalibur 4.5 and 4.6 software (LC-MS/MS)
	LabLogic Laura 6.2.3.29 (Radiodetector)
Data Evaluation:	Thermo-Scientific Xcalibur 4.5 and 4.6 software (LC-MS/MS)
	LabLogic Laura 6.2.3.29 (Radiodetector)

HPLC Method

Pre-column Type:	Phenomenex Security Guard, Phenyl, 4 x 3 mm
Column Type:	Restek Ultra Biphenyl, 250 x 4.6 mm, 5 µm
Mobile Phases:	A = 0.1% Formic Acid in Water
	B = 0.1% Formic Acid in Methanol

Gradient: Time (min) %A %B

0.0	95	5
5.0	95	5
15.0	50	50
50.0	0	100
55.0	0	100
56.0	95	5
60.0	95	5

PDA Scan Range: 190-800 nm
Flow Rate: 1.0 mL/min (Split Ratio 3:1 RAD:MS)
Autosampler Temp: 4 °C
Column Oven Temp: 40 °C

Source Settings

Table S 10: Parameters for the mass spectrometric analysis

Ionisation Mode:	Heated Electrospray Ionisation (H-ESI)
Ionisation Polarity:	Positive/Negative
Spray Voltage (+/-):	3.9 / 3.4 kV
Desolvation Temperature (Vaporizer):	300 ºC
Sheath gas flow:	35 arbitrary units
Sweep gas flow:	0 arbitrary units
Aux gas flow:	5 arbitrary units
Ion Transfer Tube Temperature:	275 ºC
RF Lens:	30 %
Collision Gas:	Nitrogen
Desolvation Gas:	Nitrogen
Damping Gas:	Helium

Scan Parameters

Internal Calibration	On (Fluoroanthene)	
Acquisition Mode:	Full Scan-MS	dd-MS^2
Detector:	Orbitrap	Orbitrap
Isolation Mode:	Quadrupole	Quadrupole
Resolution Setting:	60,000	60,000
Scan Range (*m/z*):	80-800	Data dependant
MS^2 Isolation Width (*m/z*):	-	0.4 with offset 0.0
Collision Type:	-	HCD (NCE)
Collision Energy:	-	30%

The calibration of the Thermo Orbitrap Eclipse mass spectrometer was verified using Thermo Flexmix calibration solution in positive and negative ionisation modes prior to analysis.

2. Details of Results

2.1 Degradation in surface water

2.1.1 Recovery of the applied radioactivity (AR)

Table S 11 Distribution and recovery of radioactivity of [14C]-6-PPDQ (1.5 µg/L) in surface water

Sampling Interval (DAT)	**0**			**3**			**7**			**10**		
Unit Code	A1	A2	Mean	A19	A20	Mean	A3	A4	Mean	A21	A22	Mean
Surface Water (Ext1)	96.9	100.3	98.6	91.4	93.8	92.6	91.5	89.6	90.6	102.6	93.2	97.9
Sodium Hydroxide Traps (CO_2)	NA	NA	NA	8.1	2.2	5.2	6.9	7.4	7.2	9.1	4.9	7.0
Mass Balance	96.9	100.3	98.6	99.5	96.0	97.8	98.4	97.0	97.7	111.7	98.1	104.9

Sampling Interval (DAT)	**14**			**21**			**28**			**45**		
Unit Code	A5	A6	Mean	A7	A8	Mean	A9	A10	Mean	A11	A12	Mean
Surface Water (Ext1)	87.9	69.2	78.6	87.8	84.8	86.3	82.1	88.1	85.1	77.4	59.8	68.6
Sodium Hydroxide Traps (CO_2)	11.5	30.7	21.1	14.0	13.6	13.8	18.8	15.1	17.0	19.6	44.1	31.9
Total (Mass Balance)	99.4	99.9	99.7	101.8	98.4	100.1	100.9	103.2	102.1	97.0	103.9	100.5

Sampling Interval (DAT)	**60**		
Unit Code	A13	A14	Mean
Surface Water (Ext1)	77.9	49.8	63.9
Sodium Hydroxide Traps (CO_2)	27.4	48.4	37.9
Total (Mass Balance)	105.3	98.2	101.8

NA - Not applicable, ND - Not detected (< 0.1% AR)

Table S 12 Distribution and recovery of radioactivity of [14C]-6-PPDQ (15 µg/L) in surface water

Sampling Interval (DAT)	**0**			**3**			**7**			**10**		
Unit Code	B1	B2	Mean	B19	B20	Mean	B3	B4	Mean	B21	B22	Mean
Surface Water (Ext1)	98.6	98.1	98.4	96.3	96.6	96.5	90.8	93.3	92.1	93.7	93.6	93.7
Sodium Hydroxide Traps (CO_2)	NA	NA	NA	0.5	1.4	1.0	4.3	4.0	4.2	3.5	3.6	3.6
Total (Mass Balance)	98.6	98.1	98.4	96.8	98.0	97.4	95.1	97.3	96.2	97.2	97.2	97.2

Sampling Interval (DAT)	**14**			**21**			**28**			**45**		
Unit Code	B5	B6	Mean	B7	B8	Mean	B9	B10	Mean	B11	B12	Mean
Surface Water (Ext1)	79.7	88.3	84.0	83.4	83.4	83.4	83.7	83.2	83.5	67.6	54.6	61.1
Sodium Hydroxide Traps (CO_2)	19.0	7.1	13.1	15.0	13.7	14.4	12.8	13.7	13.3	28.9	43.4	36.2
Total (Mass Balance)	98.7	95.4	97.1	98.4	97.1	97.8	96.5	96.9	96.7	96.5	98.0	97.3

Sampling Interval (DAT)	**60**		
Unit Code	B13	B14	Mean
Surface Water (Ext1)	68.4	66.3	67.4
Sodium Hydroxide Traps (CO_2)	29.5	29.7	29.6
Total (Mass Balance)	97.9	96.0	97.0

NA - Not applicable, ND - Not detected (< 0.1% AR)

Table S 13 Distribution and recovery of radioactivity of [14C]-6-PPDQ (15 µg/L, sterile) in surface water

Sampling Interval (DAT)		**60**	
Unit Code	C1	C2	Mean
Surface Water (Ext1)	85.7	87.2	86.5
Sodium Hydroxide Traps (CO_2)	10.3	8.1	9.2
Mass Balance	96.0	95.3	95.7

ND - Not detected (< 0.1% AR)

2.1.2 Kinetic Results

Table S 14 Results of the Single First Order (SFO) kinetic modeling of biodegradation of [14C]-6-PPDQ in surface water

System	Test concentration (µg/L)	$DegT_{50}$ (days)	$DegT_{90}$ (days)	k ($days^{-1}$)	Chi^2	r^2	Prob > t
Low test concentration	1.5	11.8	39.1	0.05894	8.27	0.9638	3×10^{-10}
High test concentration	15	16.9	56.1	0.04102	7.54	0.9338	1.5×10^{-10}

Note: SFO: single first order kinetics. $DT_{50:}$ Calculated degradation half-life of [^{14}C]-6PPD-Q. χ^2: chi-square statistical value. r^2: coefficient of determination.

Figure S 6 Distribution of applied radioactivity of [14C]-6-PPDQ (1.5 µg/L) during biodegradation in water

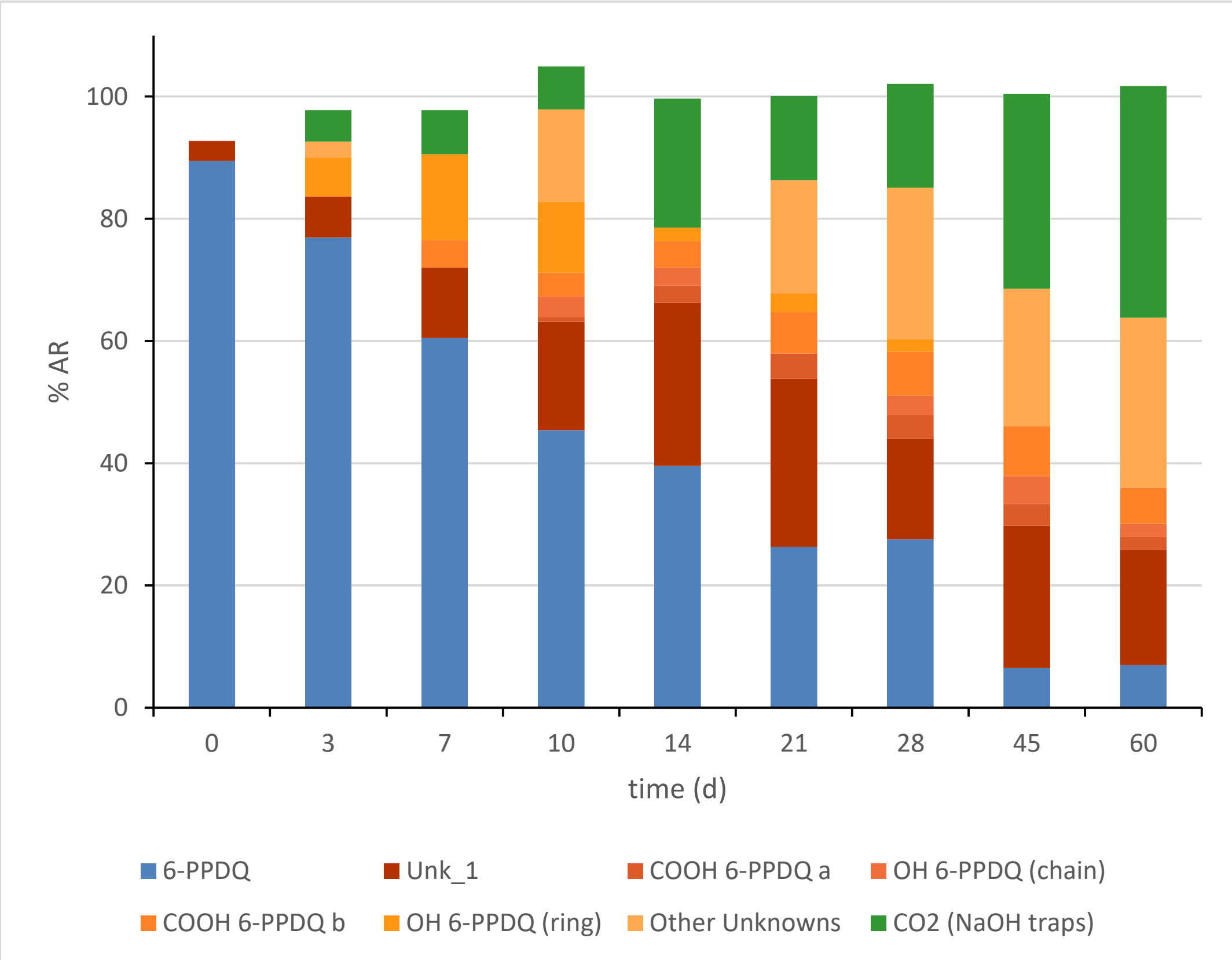

Figure S 7 Distribution of applied radioactivity of [14C]-6-PPDQ (15 µg/L) during biodegradation in water

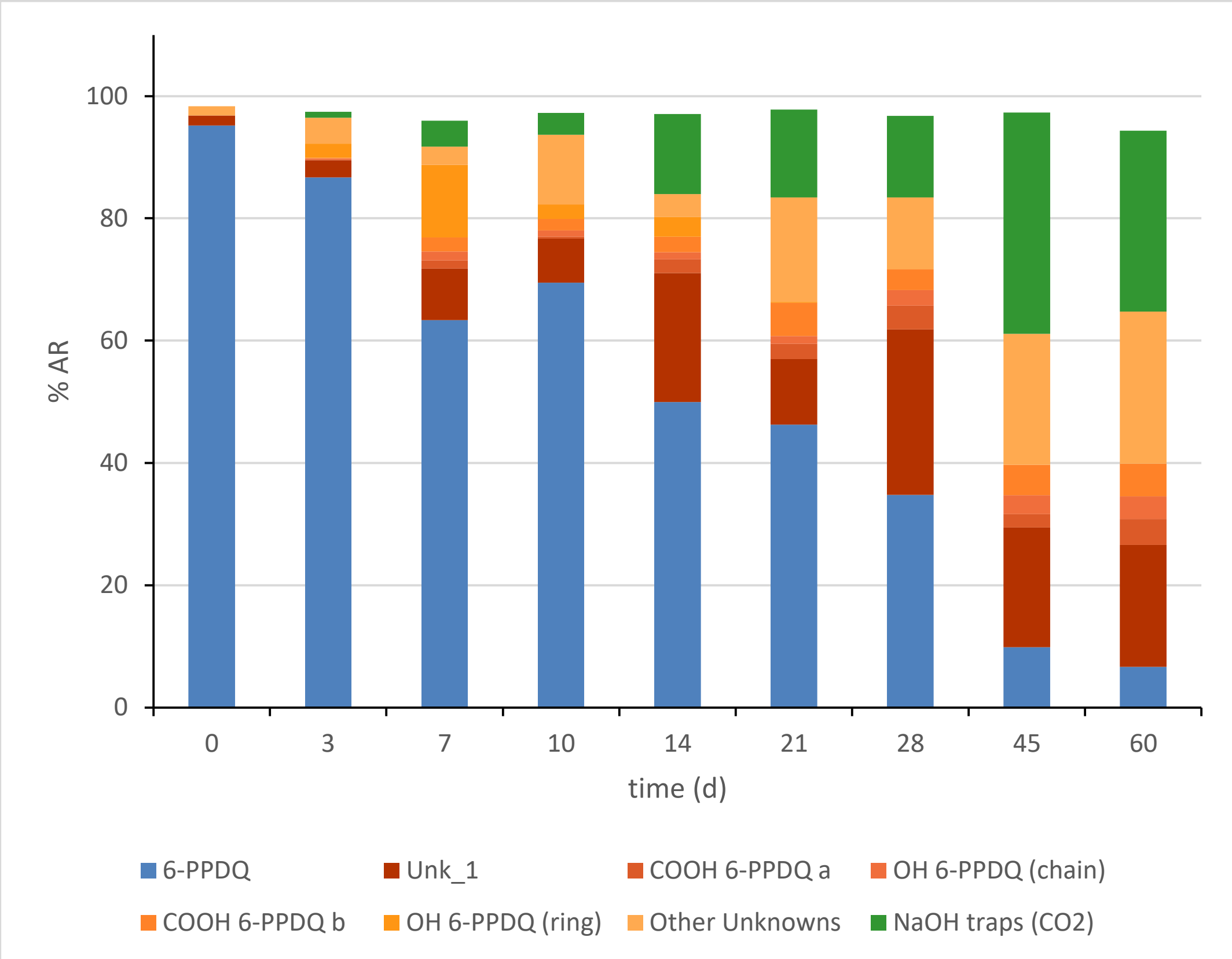

2.1.3 Chromatograms

Figure S 8 Radio-chromatograms obtained from samples of 6-PPDQ biodegradation in water

0 DAT Water (B1, Extract 1) – 98.6% of applied radioactivity (9CHR010 Run 1)

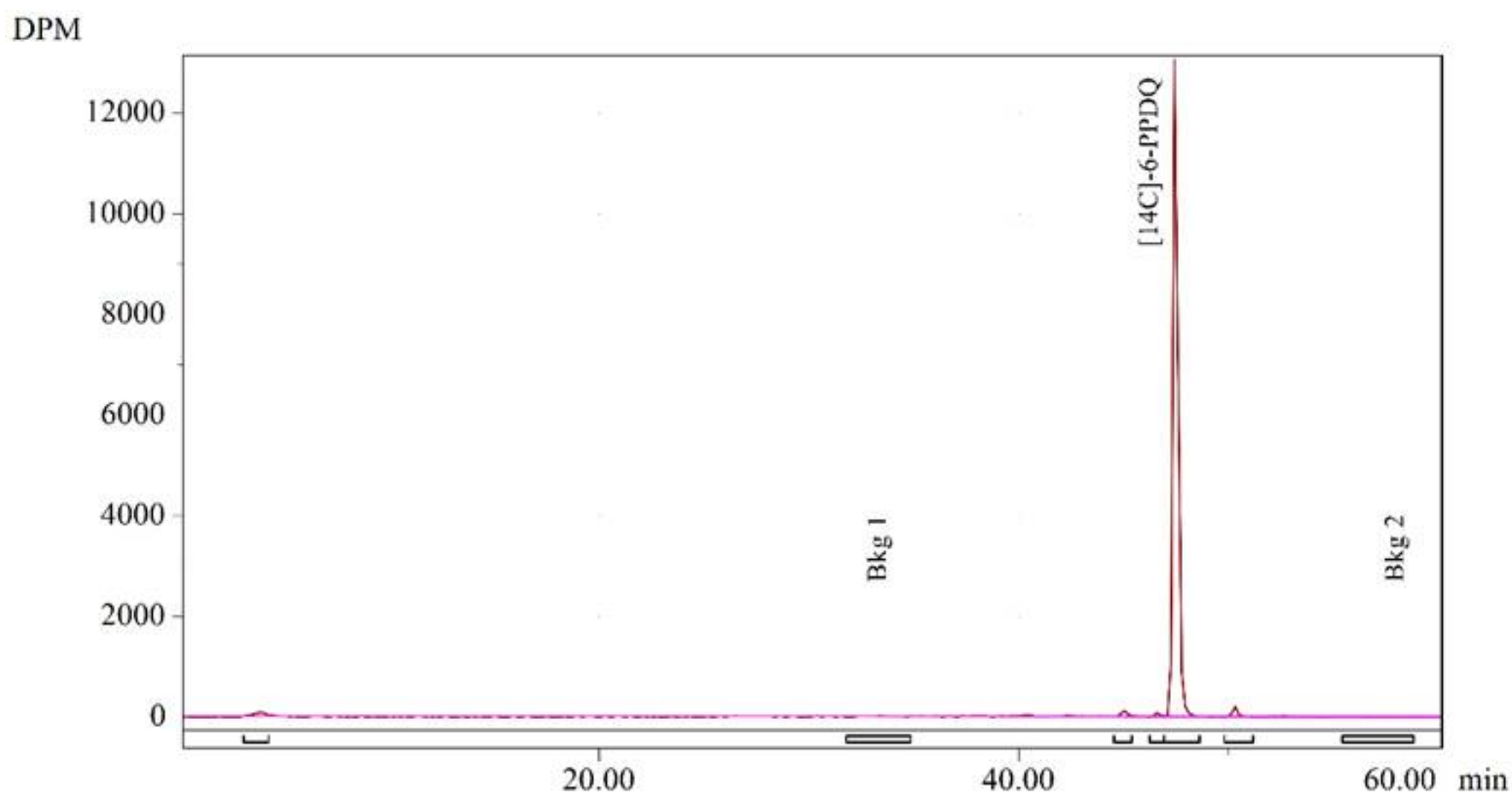

14 DAT Water (B5, Extract 1) – 79.7% of applied radioactivity (9CHR021 Run 1)

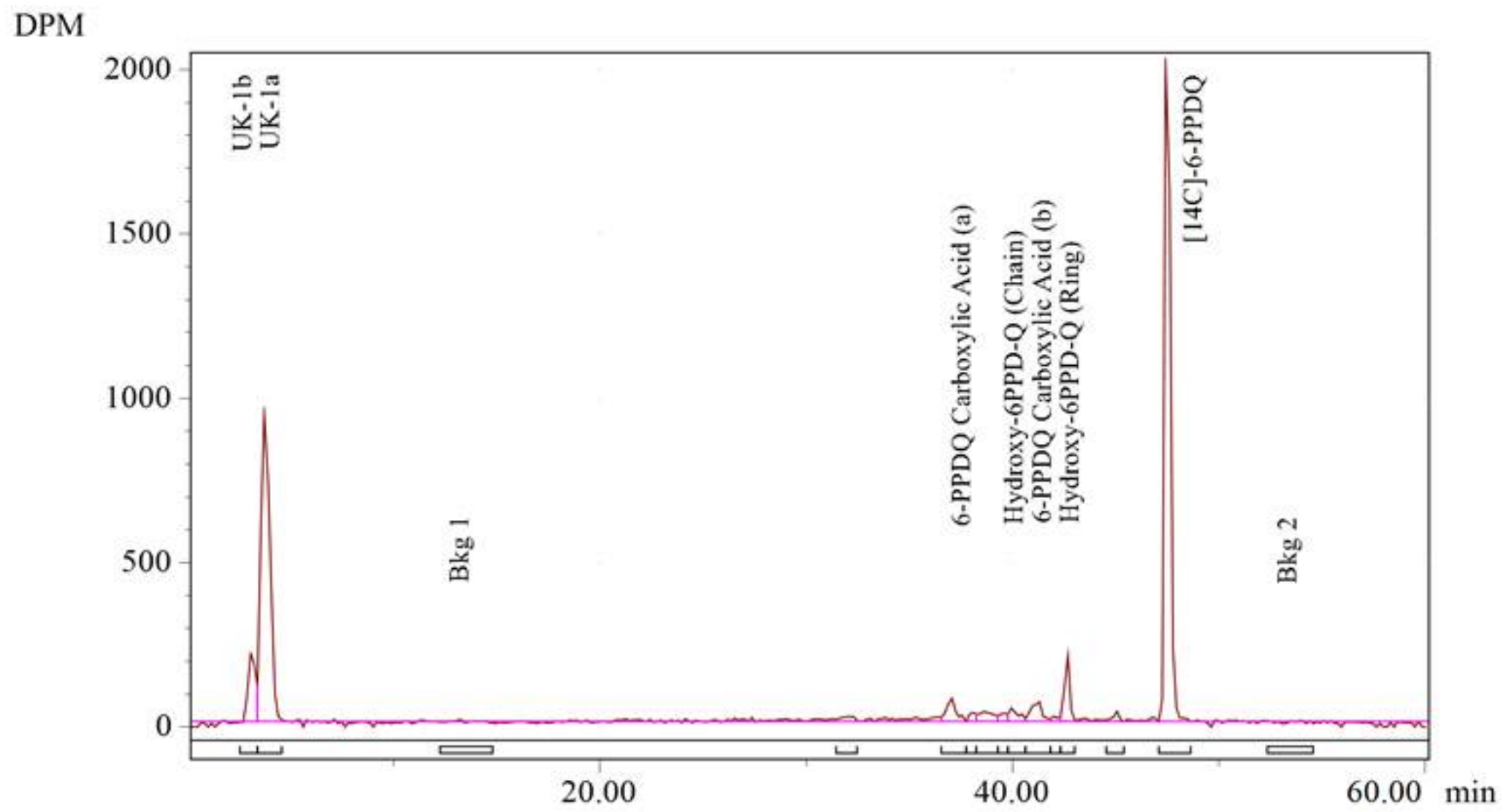

60 Day Water (B14, Extract 2) – 66.3% of applied radioactivity (9CHR055 Run 1)

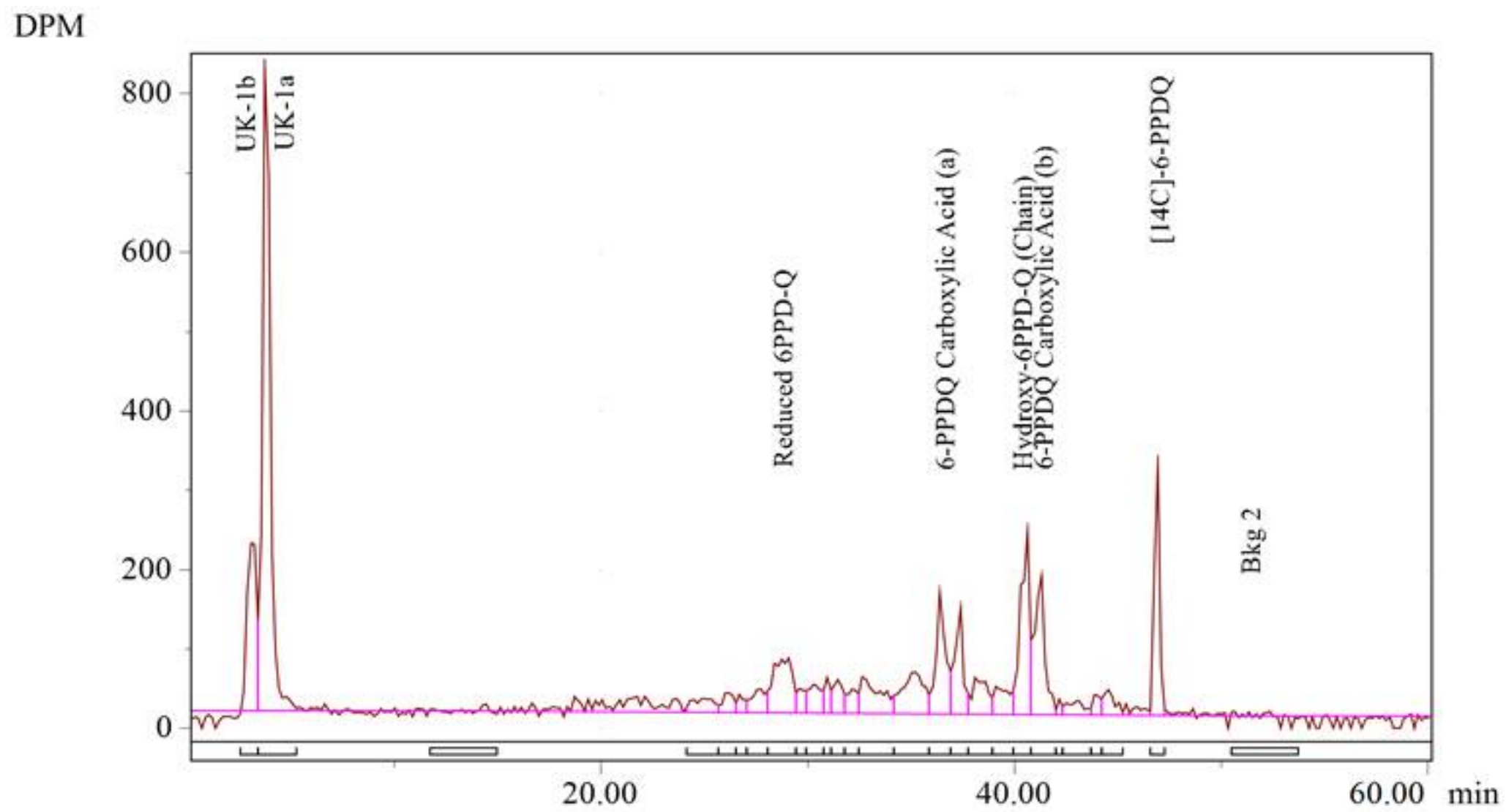

2.1.4 Mass spectrometric data of the transformation products

Table S 15 Mass spectrometric data of 6-PPDQ and its transformation products detected by LC-MS/MS, their occurrence in different experiments and reports in literature.

	Ionis. mode	retent. time	exact mass of molec. ion	assigned sum formular	detected fragment ion	formula of fraagment ion	fragment ion (smiles code)	Confid. level	detection in aerobic experiments			detection in anaerobic experiments		previous reports
		(min)							surface water	water-sediment	soil	water-sediment	soil	
6-PPDQ	+	47.26	299.1754	C18H23N2O2	256.1206	C15H16N2O2	O=C1C=C(Nc2ccccc2)C(=O)C=C1NC(C)CC(C)C	1						
		43.71 (soil)			215.0816	C12H11N2O2								
					187.0866	C11H11N2O								
OH-6-PPDQ (Ring)	+	42.39	315.1705	C18H23N2O3	257.0925	C15H17N2O2	OC1=C(Nc2ccccc2)C(=O)C=C(NC(C)CC(C)C)C1=O	3	x	x		x		Foscari et al. (2024)
		37.7 (soil)			231.0766	C12H11N2O3								Han et al. (2025)
					203.0817	C11H11N2O2								Shen et al. (2025)
					186.0552	C11H8NO2								
					100.1119	C6H14N								
					85.1011	C6H13								
OH-6-PPDQ (Side-Chain I)	+	40.7	315.1705	C18H23N2O3	297.16	C18H21N2O2	O=C1C=C(Nc2ccccc2)C(=O)C=C1NC(C)CC(C)CO	3	x	x	x	x		Foscari et al. (2024)
		35.7 (soil)			257.1258	C15H17N2O2	O=C1C=C(Nc2ccccc2)C(=O)C=C1NC(CC(C)C)CO							Han et al. (2025)
					241.0972	C14H13N2O2								Shen et al. (2025)
					227.0816	C13H11N2O								

					215.0816	C12H11N2O2								
					187.0866	C11H11N2O								
COOH-6-PPDQ (Side-Chain I)	+	40.6	329.1499	C18H21N2O4	311.1394	C18H19N2O3	O=C1C=C(Nc2ccccc2)C(=O)C=C1NC(C)CC(C)C(=O)O	3	x	x				Foscari et al. (2024)
		36.11 (soil)			283.1444	C17H19N2O2								Han et al. (2025)
					241.0973	C14H13N2O2								
					200.0706	C12H10NO2								
					187.0866	C11H11N2O								
					112.0756	C6H10NO								
COOH-6-PPDQ (Side-Chain II)	+	41.33	329.15	C18H19N2O3	311.1394	C18H19N2O3	O=C1C=C(Nc2ccccc2)C(=O)C=C1NC(CC(C)C)C(=O)O	3	x	x	x			
		36.94 (soil)		C17H19N2O2	283.1444	C17H19N2O2								
					241.0973	C14H13N2O2								
					200.0706	C12H10NO2								
					187.0866	C11H11N2O								
					112.0756	C6H10NO								
TP211	+	29.06	212.1645	C12H22NO2	128.0705	C6H10NO2	O=C1C=C(NC(C)CC(C)C)C(O)CC1	3	x	x		x		Shen et al. (2025)
		24.98 (soil)			110.0599	C6H8NO								
					83.0489	C5H7O								
					67.054	C5H7								

Figure S 9: LC-MS chromatograms and spectra of suspected OH-6-PPDQ, Ring

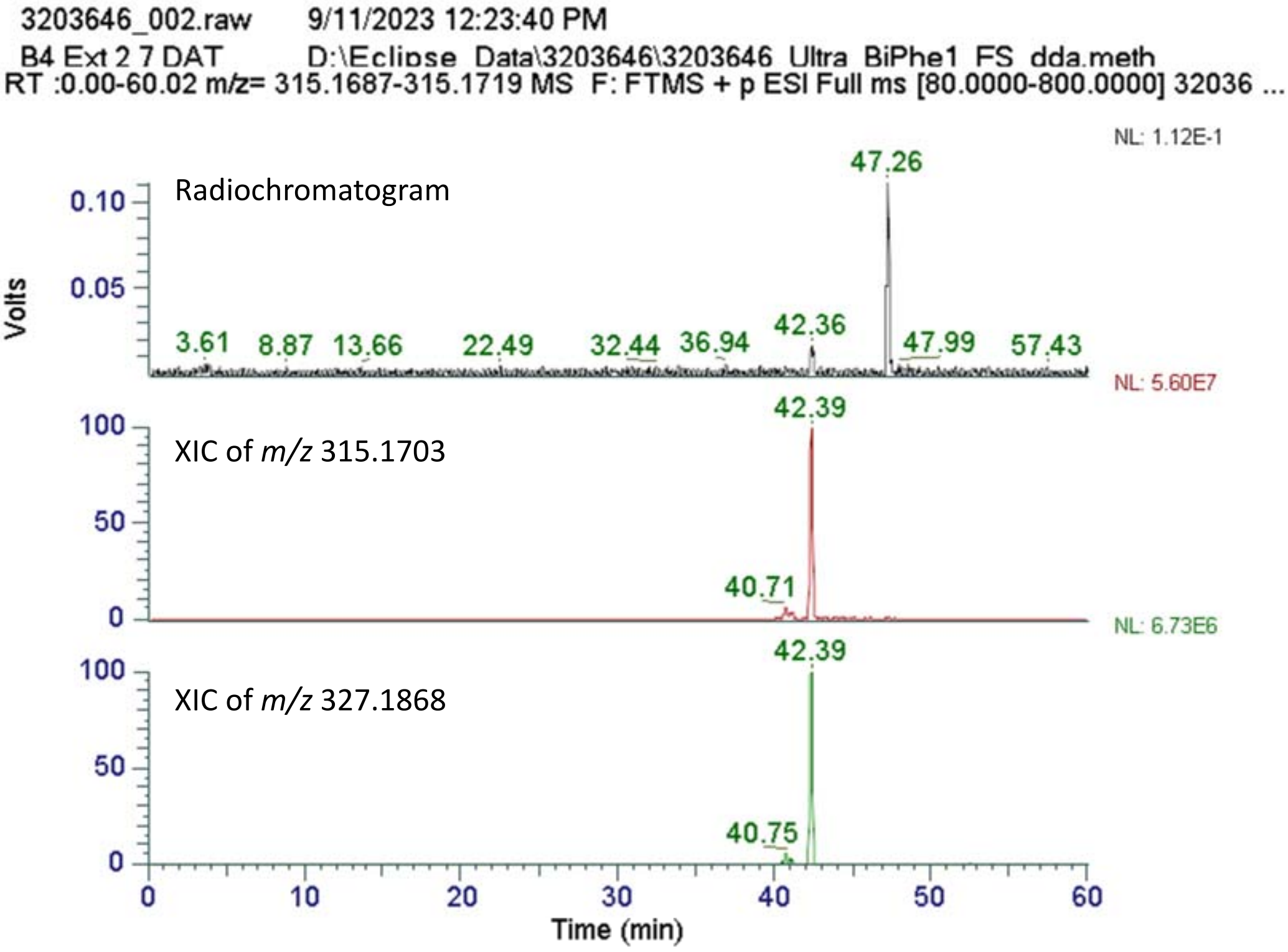

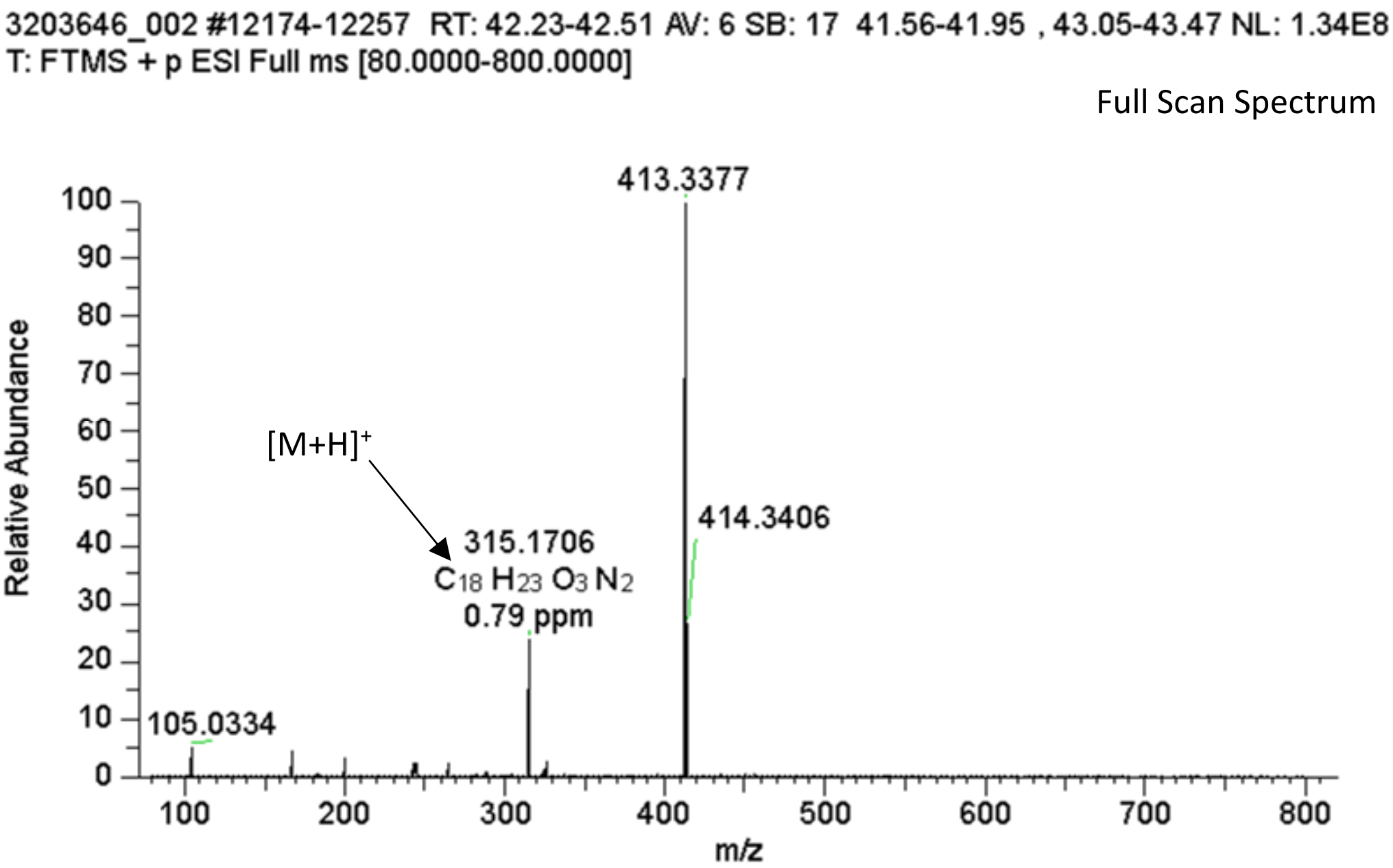

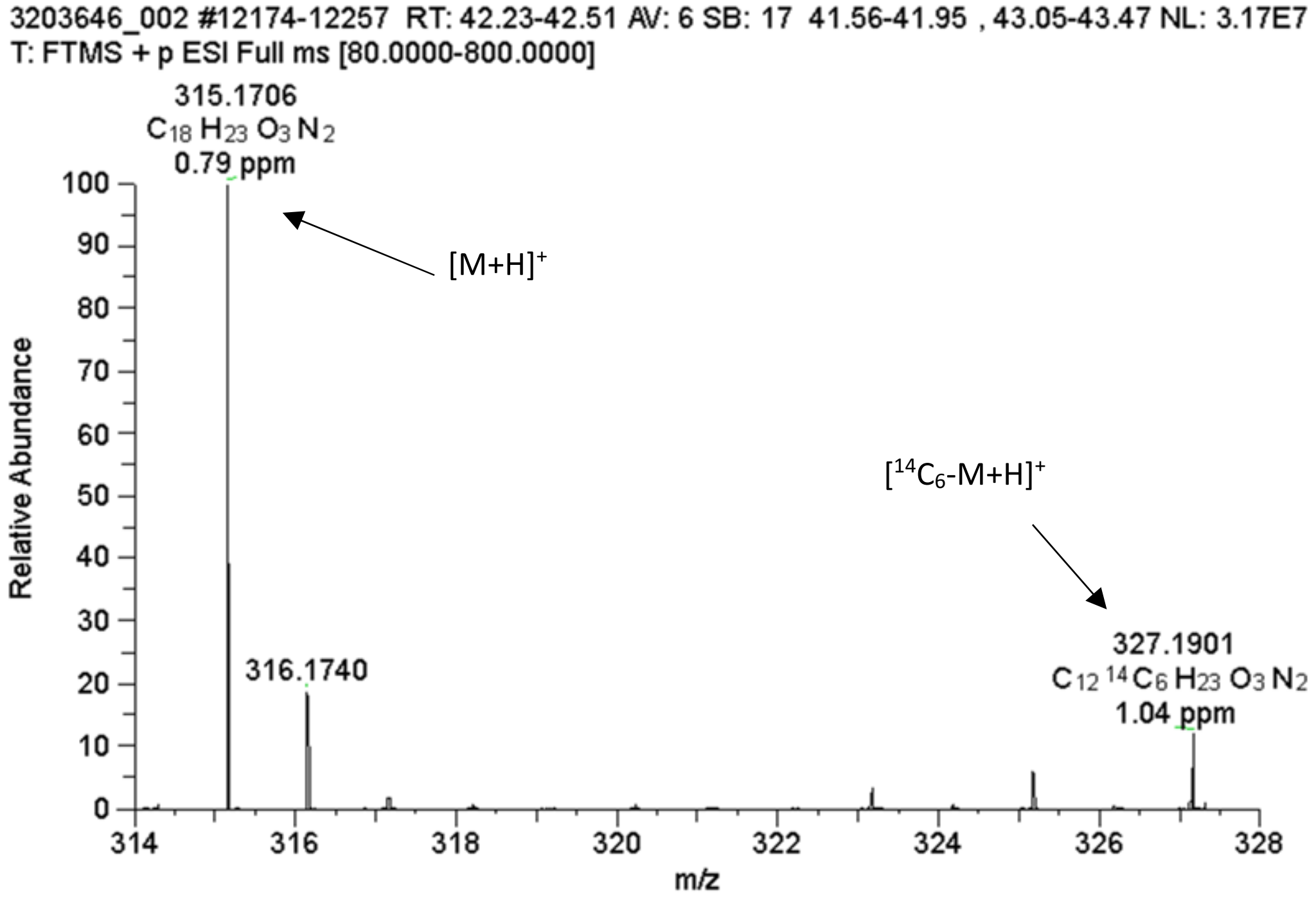

3203646_002 #12174-12257 RT: 42.23-42.51 AV: 6 SB: 17 41.56-41.95 , 43.05-43.47 NL: 3.17E7
T: FTMS + p ESI Full ms [80.0000-800.0000]
315.1706
C18 H23 O3 N2
0.79 ppm
[M+H]+
[14C6-M+H]+
327.1901
C12 14C6 H23 O3 N2
1.04 ppm
316.1740
Relative Abundance
m/z

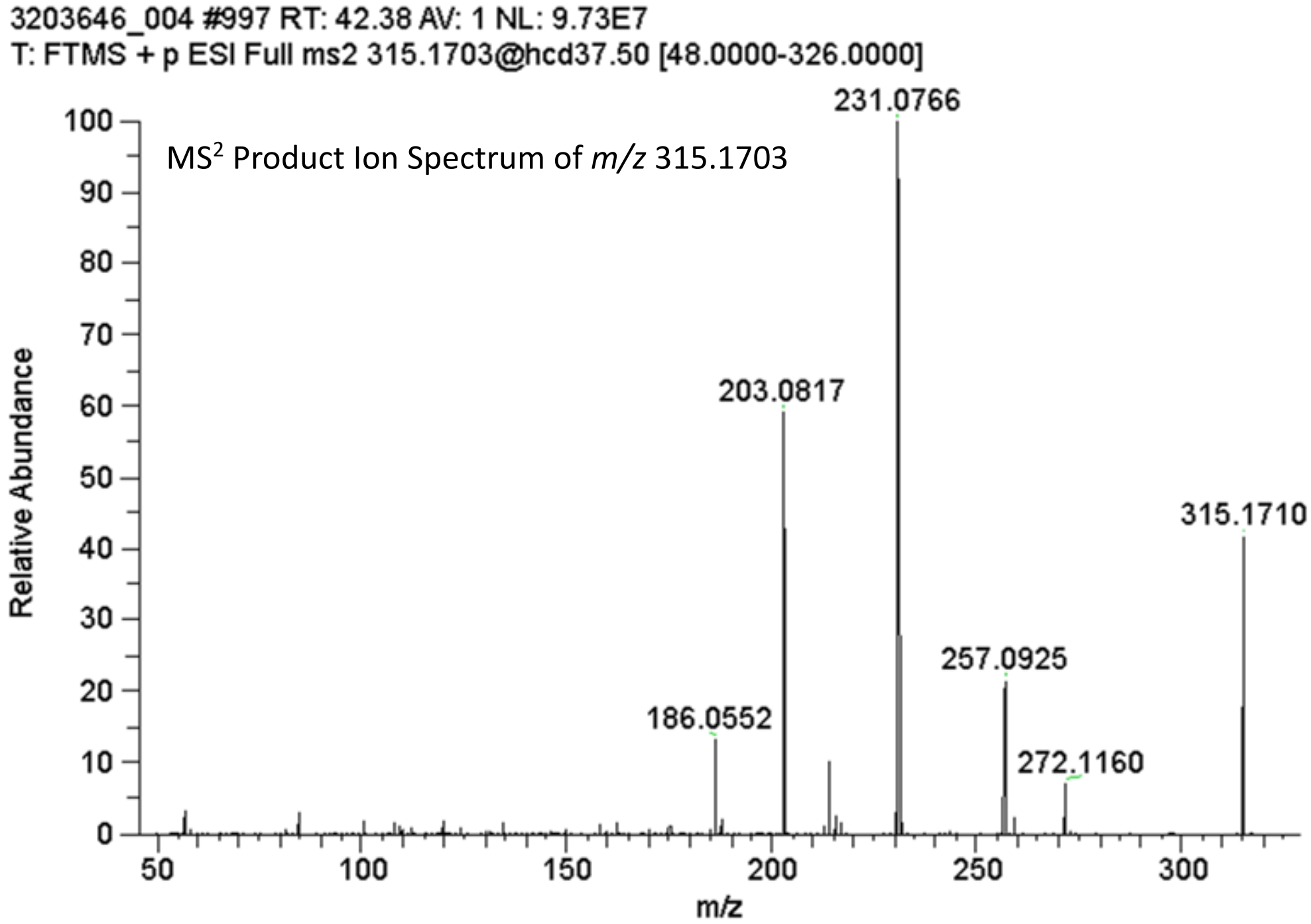
3203646_004 #997 RT: 42.38 AV: 1 NL: 9.73E7
T: FTMS + p ESI Full ms2 315.1703@hcd37.50 [48.0000-326.0000]
MS2 Product Ion Spectrum of m/z 315.1703
231.0766
203.0817
315.1710
257.0925
186.0552
272.1160
Relative Abundance
m/z

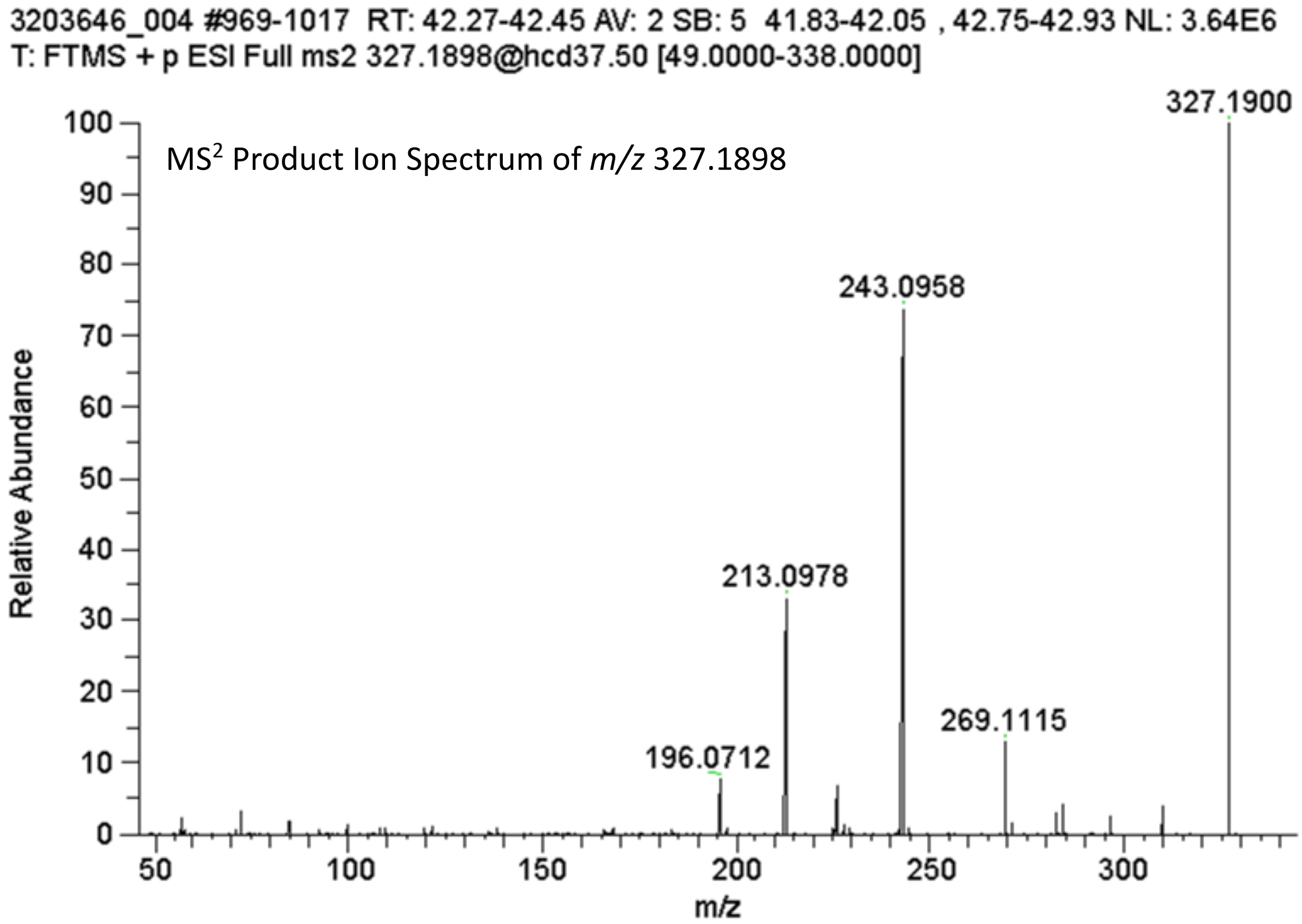
3203646_004 #969-1017 RT: 42.27-42.45 AV: 2 SB: 5 41.83-42.05 , 42.75-42.93 NL: 3.64E6
T: FTMS + p ESI Full ms2 327.1898@hcd37.50 [49.0000-338.0000]
MS2 Product Ion Spectrum of m/z 327.1898
327.1900
243.0958
213.0978
269.1115
196.0712
Relative Abundance
m/z

Figure S 10: LC-MS chromatograms and spectra of suspected OH-6-PPDQ, side-chain

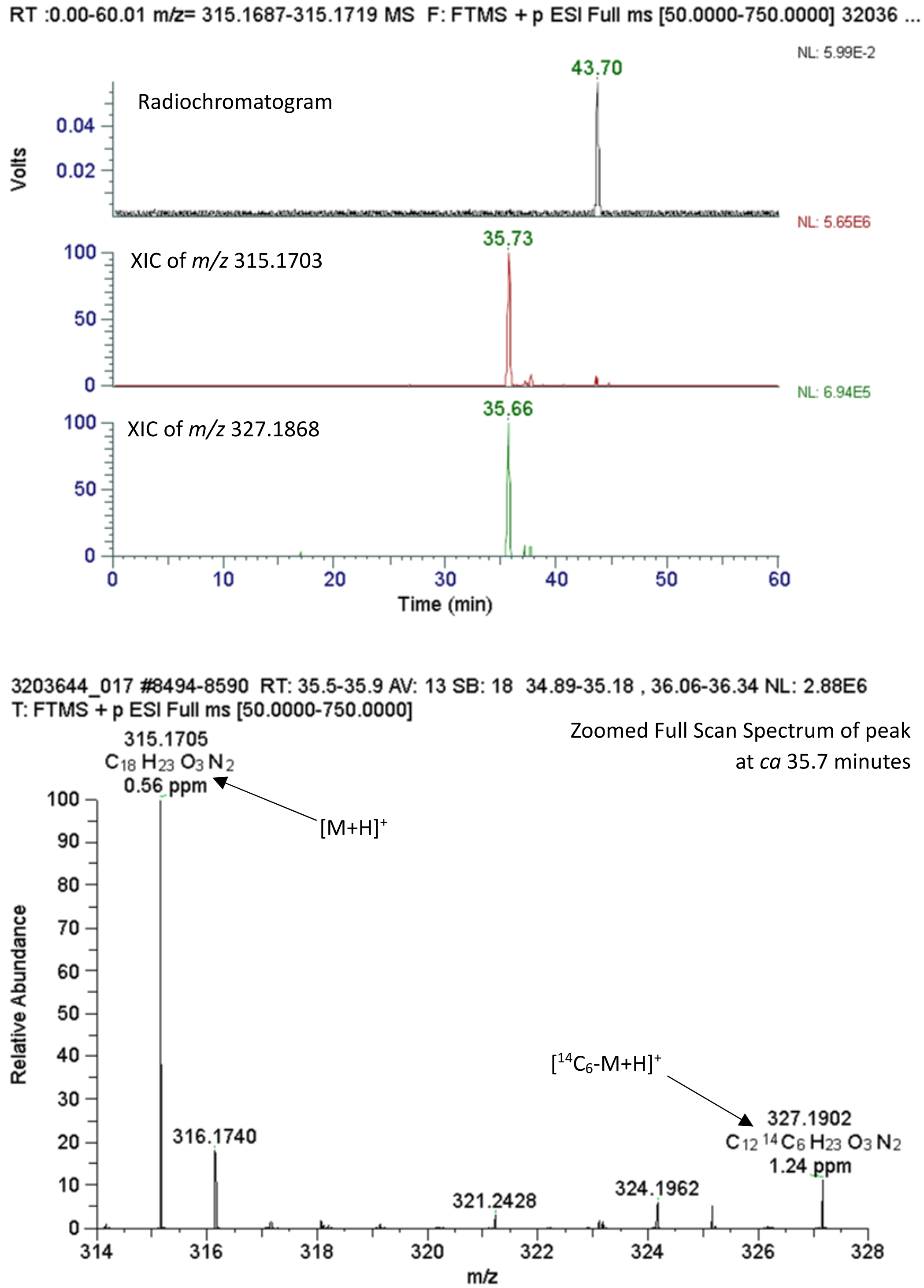

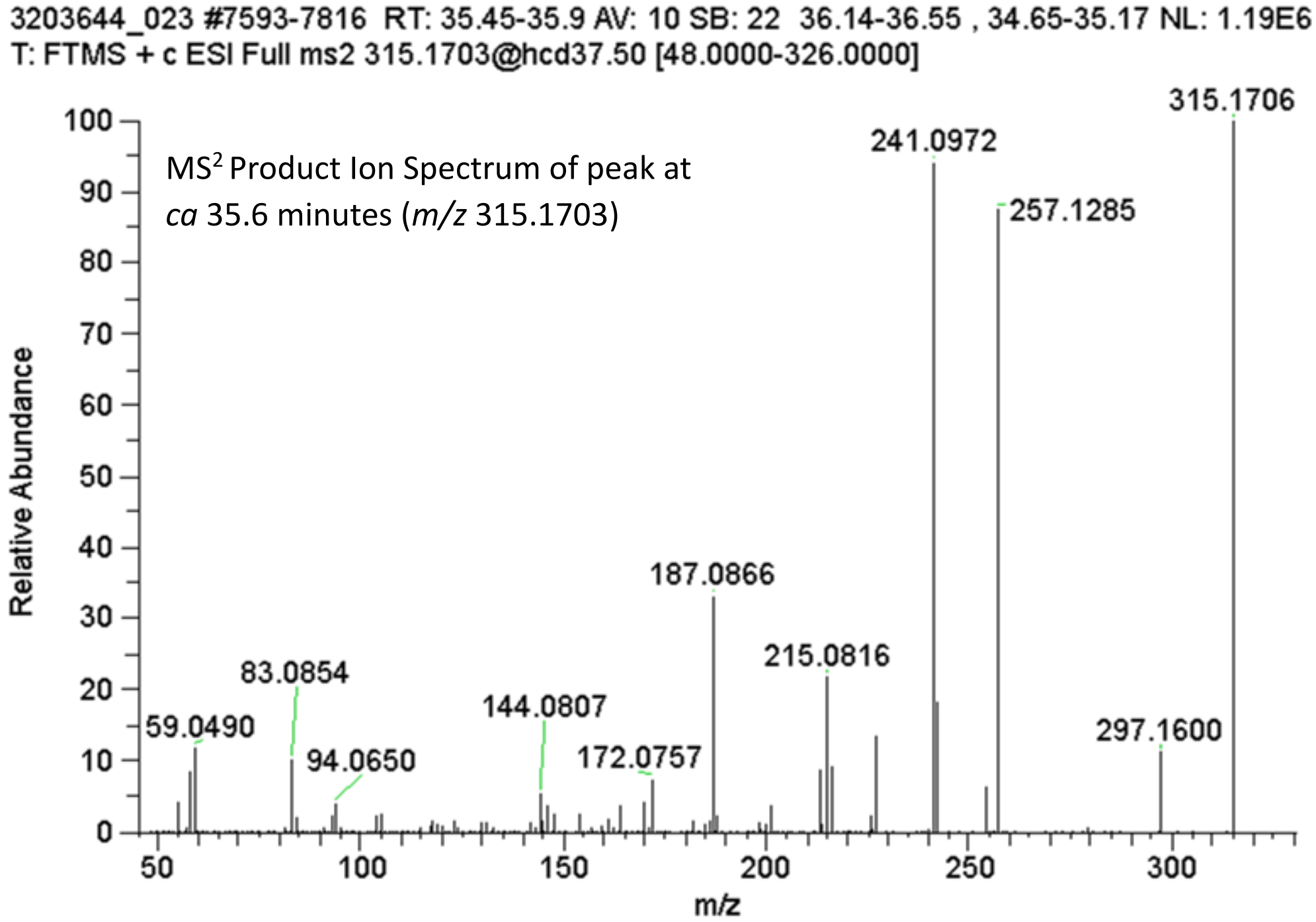
3203644_023 #7593-7816 RT: 35.45-35.9 AV: 10 SB: 22 36.14-36.55 , 34.65-35.17 NL: 1.19E6
T: FTMS + c ESI Full ms2 315.1703@hcd37.50 [48.0000-326.0000]
MS2 Product Ion Spectrum of peak at
ca 35.6 minutes (m/z 315.1703)
315.1706
241.0972
257.1285
187.0866
215.0816
83.0854
144.0807
59.0490
297.1600
94.0650
172.0757
Relative Abundance
m/z

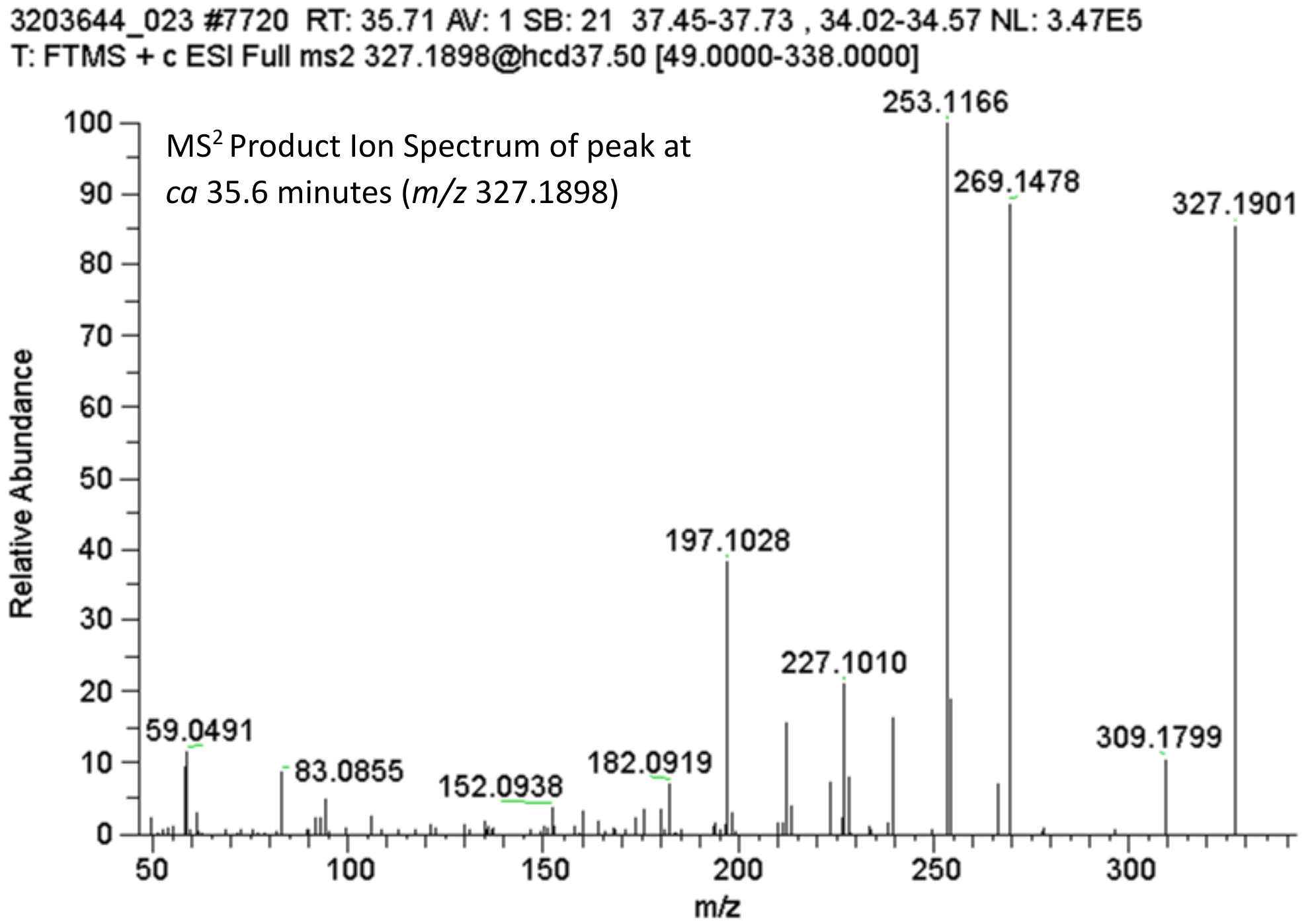
3203644_023 #7720 RT: 35.71 AV: 1 SB: 21 37.45-37.73 , 34.02-34.57 NL: 3.47E5
T: FTMS + c ESI Full ms2 327.1898@hcd37.50 [49.0000-338.0000]
MS2 Product Ion Spectrum of peak at
ca 35.6 minutes (m/z 327.1898)
253.1166
269.1478
327.1901
197.1028
227.1010
59.0491
309.1799
83.0855
152.0938
182.0919
Relative Abundance
m/z

Figure S 11: LC-MS chromatograms and spectra of suspected COOH-6-PPDQ, side-chain

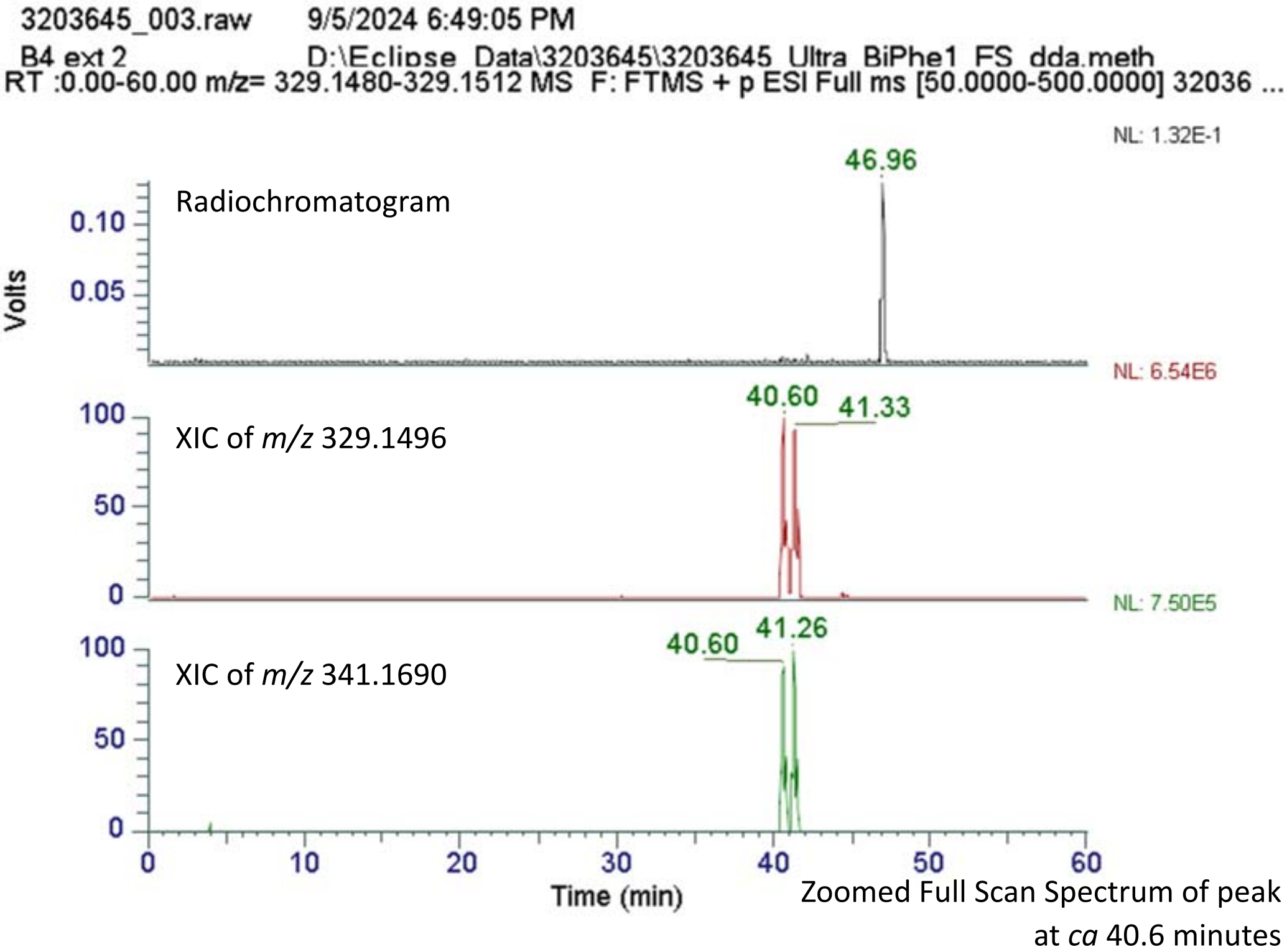

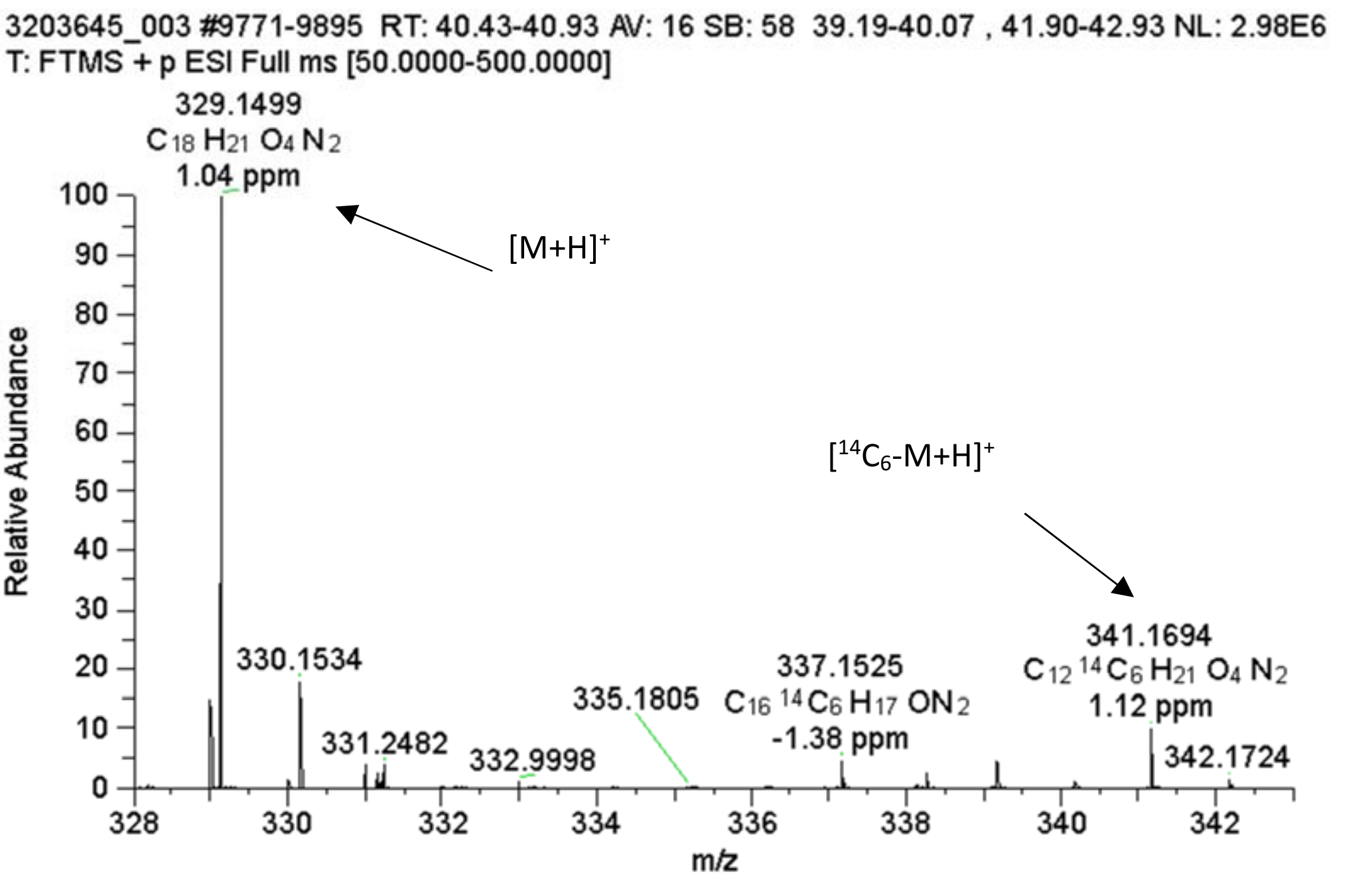

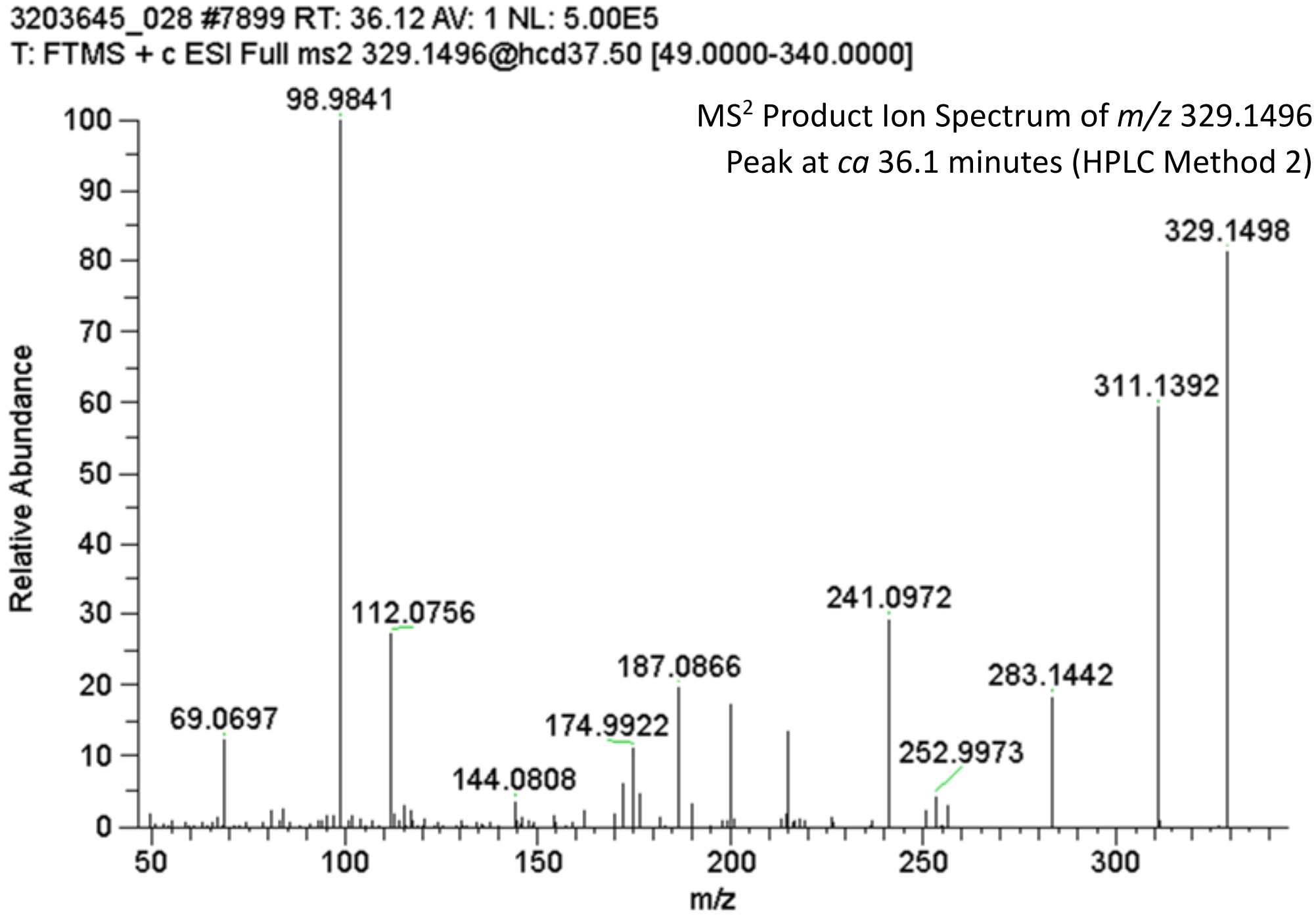

3203645_028 #7899 RT: 36.12 AV: 1 NL: 5.00E5
T: FTMS + c ESI Full ms2 329.1496@hcd37.50 [49.0000-340.0000]
MS² Product Ion Spectrum of m/z 329.1496
Peak at ca 36.1 minutes (HPLC Method 2)
98.9841
329.1498
311.1392
241.0972
112.0756
187.0866
283.1442
69.0697
174.9922
252.9973
144.0808
Relative Abundance
m/z

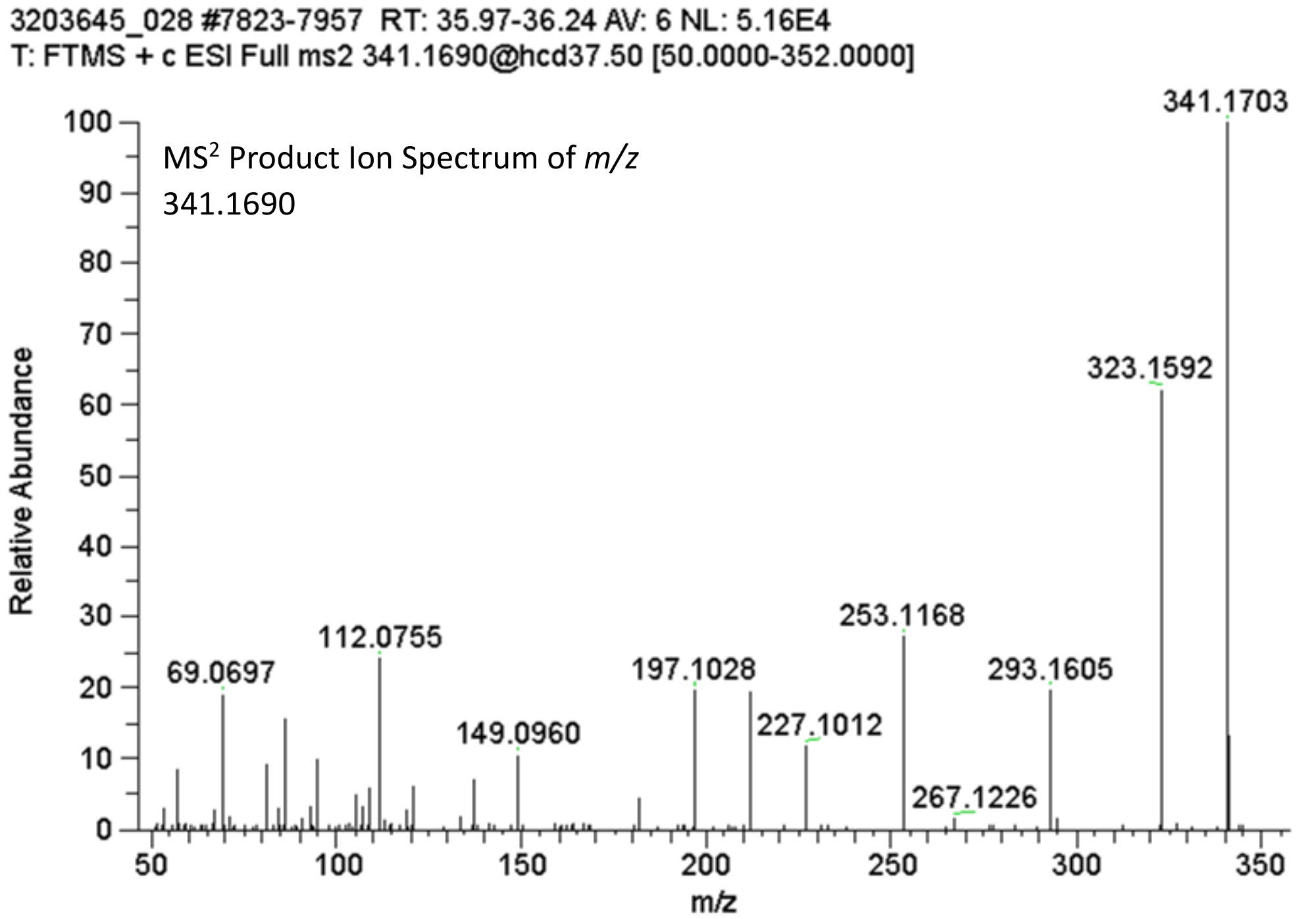

3203645_028 #7823-7957 RT: 35.97-36.24 AV: 6 NL: 5.16E4
T: FTMS + c ESI Full ms2 341.1690@hcd37.50 [50.0000-352.0000]
MS² Product Ion Spectrum of m/z
341.1690
341.1703
323.1592
253.1168
112.0755
69.0697
197.1028
293.1605
227.1012
149.0960
267.1226
Relative Abundance
m/z

Figure S 12: LC-MS chromatograms and spectra of TP 211

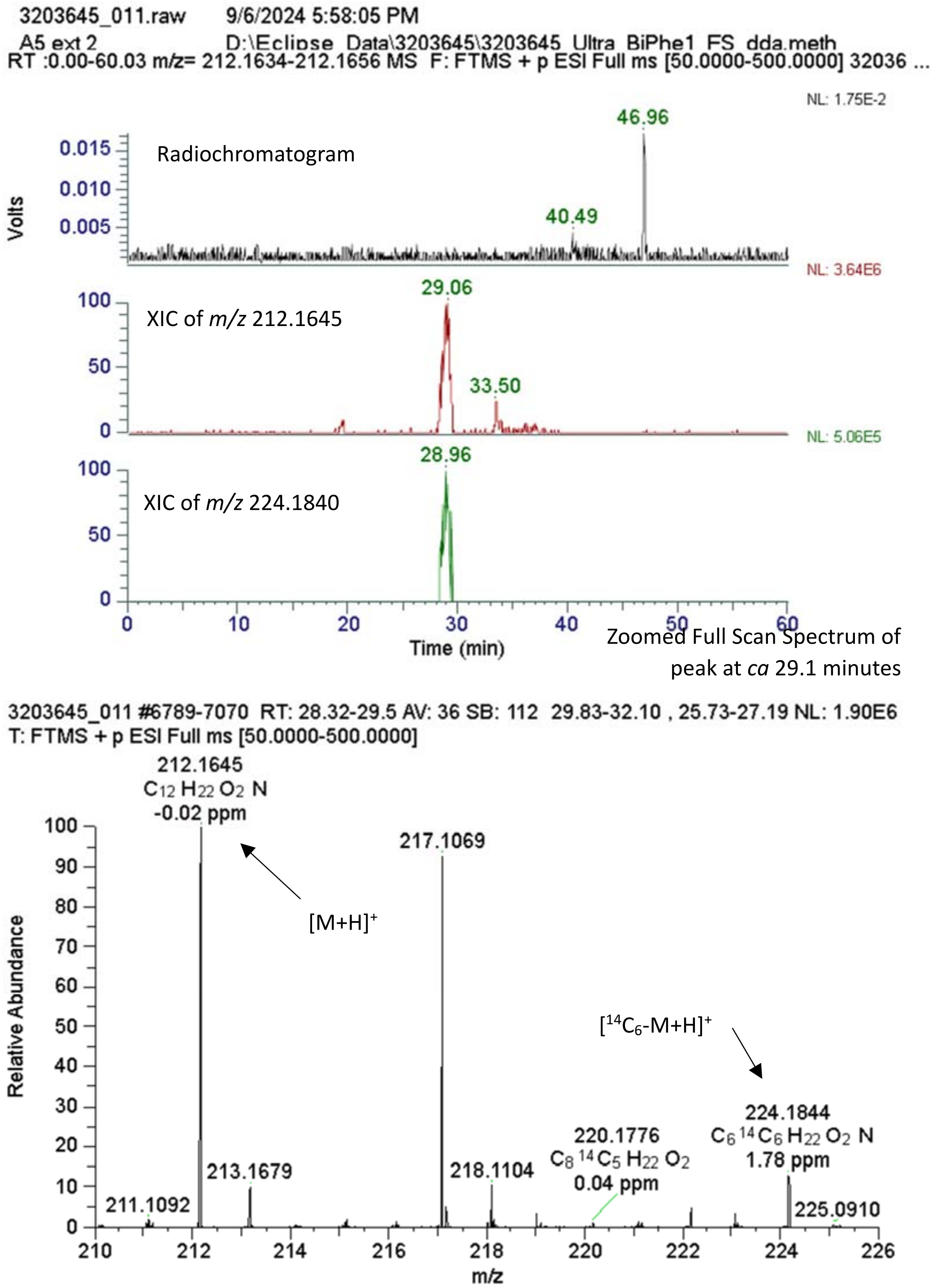

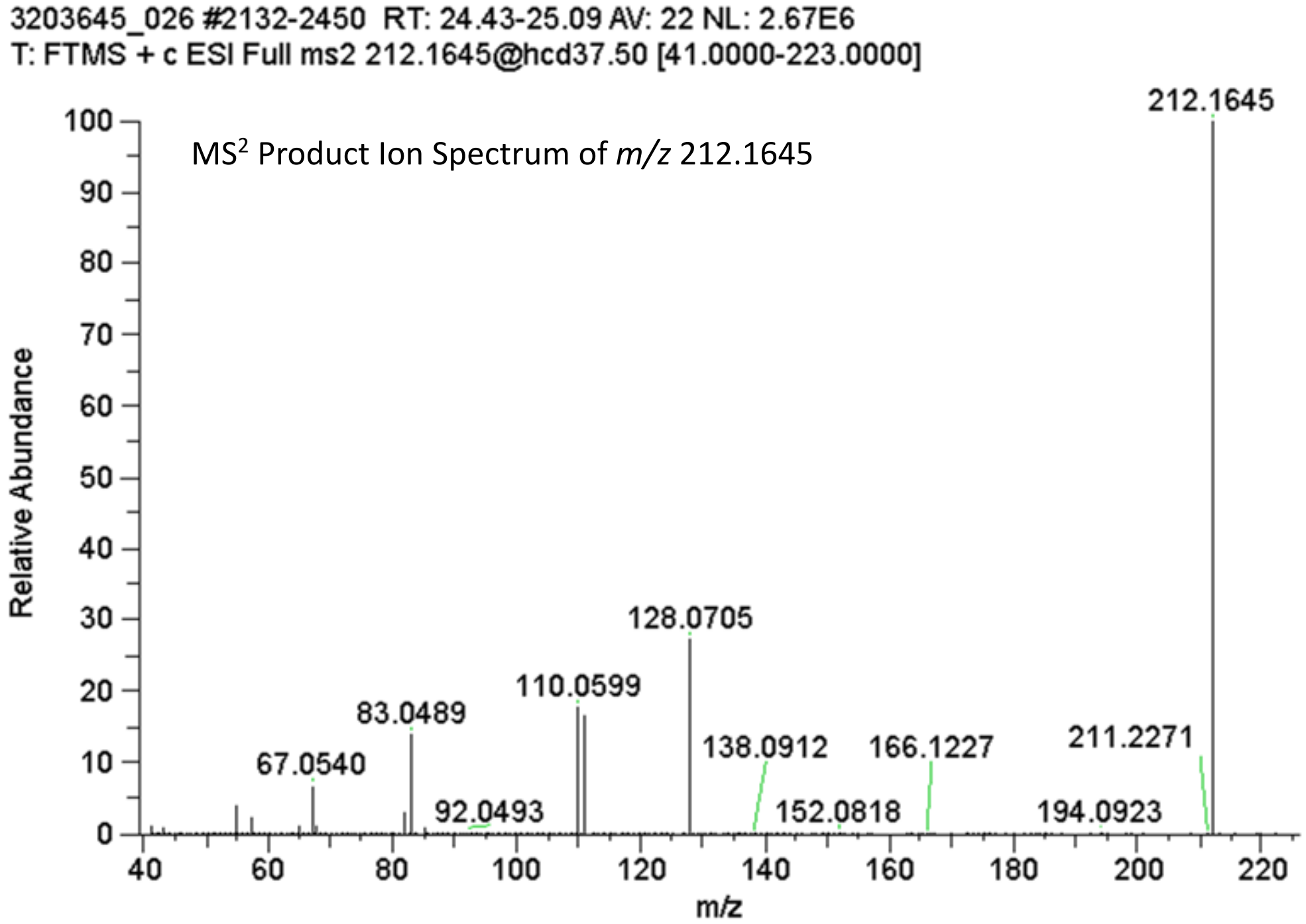
3203645_026 #2132-2450 RT: 24.43-25.09 AV: 22 NL: 2.67E6
T: FTMS + c ESI Full ms2 212.1645@hcd37.50 [41.0000-223.0000]
MS2 Product Ion Spectrum of m/z 212.1645
212.1645
128.0705
110.0599
83.0489
67.0540
92.0493
138.0912
152.0818
166.1227
194.0923
211.2271
Relative Abundance
m/z

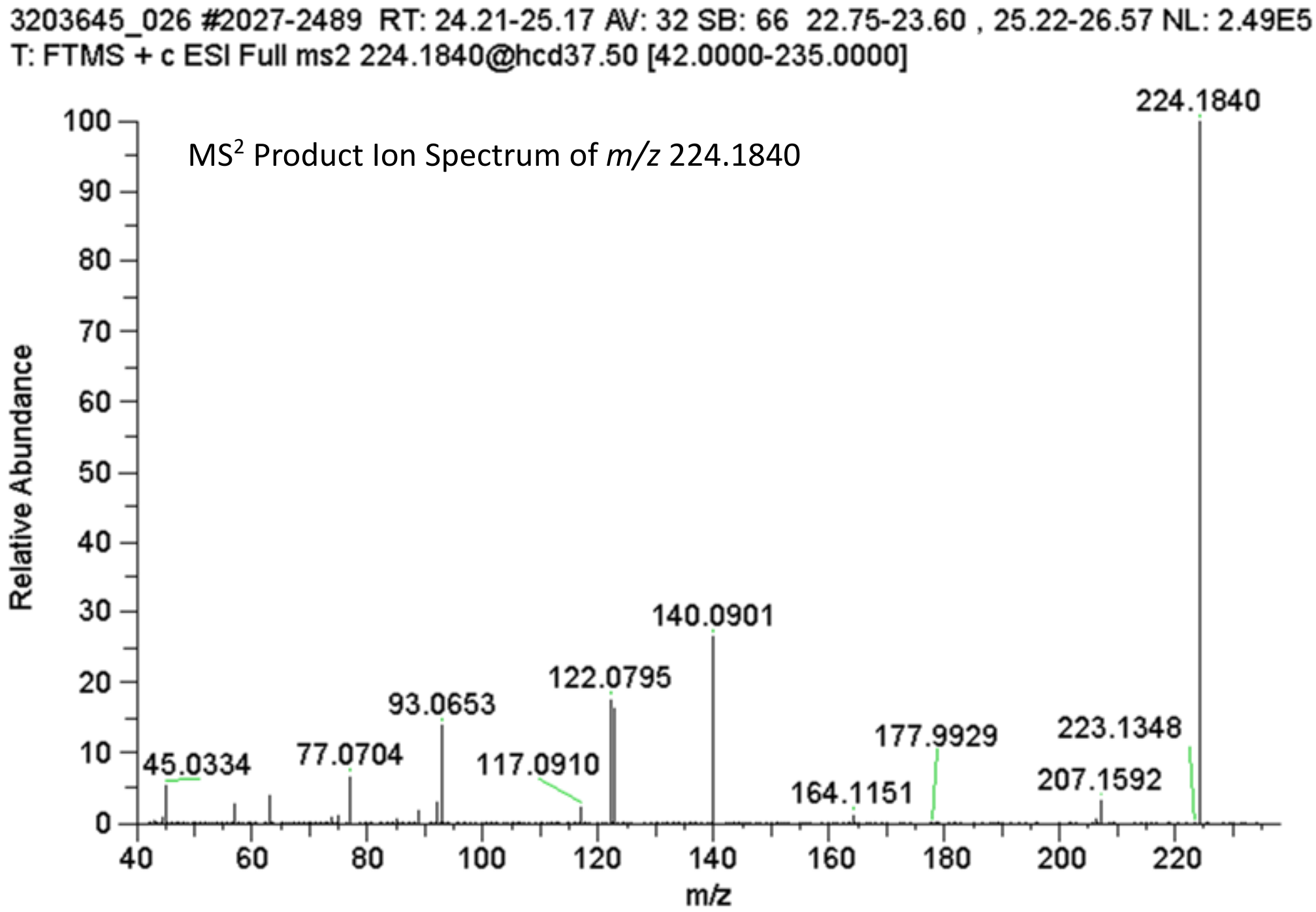
3203645_026 #2027-2489 RT: 24.21-25.17 AV: 32 SB: 66 22.75-23.60 , 25.22-26.57 NL: 2.49E5
T: FTMS + c ESI Full ms2 224.1840@hcd37.50 [42.0000-235.0000]
MS2 Product Ion Spectrum of m/z 224.1840
224.1840
140.0901
122.0795
93.0653
45.0334
77.0704
117.0910
164.1151
177.9929
207.1592
223.1348
Relative Abundance
m/z

2.2 Water-sediment systems

2.2.1 Recovery of the applied radioactivity (AR)

Table S 16 Recovery of applied radioactivity (% AR) in the biodegradation of [14C]-6-PPDQ in water-sediment systems

System	Sampling Time (DAT)	Overlying Water	Sediment Extracts	Unextracted from Sediment	Traps	Mass Balance
Calwich Abbey Aerobic	0	89.9	10.0	0.3	NA	100.1
	7	20.0	66.7	8.2	2.3	97.1
	29	7.6	55.0	24.7	9.2	96.4
	100	2.4	30.1	41.9	21.6	95.9
Middle Pond Aerobic	0	86.0	12.8	0.4	NA	99.1
	7	35.4	51.3	9.1	1.8	97.6
	29	9.6	46.3	28.9	10.8	95.6
	100	2.6	17.5	42.9	31.7	94.7
Calwich Abbey Anaerobic	0	91.0	7.6	0.2	NA	98.7
	7	57.9	38.7	1.7	0.6	98.8
	29	32.8	53.8	9.0	2.2	97.7
	100	15.3	49.0	24.4	5.6	94.2
Middle Pond Anaerobic	0	94.0	6.0	0.2	NA	100.2
	7	76.3	21.2	1.6	0.5	99.4
	29	43.1	44.9	9.7	1.8	99.4
	100	13.0	51.4	28.5	2.8	95.6

NA = Not applicable

2.2.2 Kinetic data

Table S 17 Kinetic data of [^{14}C]-6-PPDQ biodegradation in the two water sediment systems under aerobic and anaerobic conditions

Test Conditions	Overlying water (FOMC)					
	$DegT_{50}$ [days]	$DegT_{90}$ [days]	χ^2%	Parent_0	alpha	beta
Calwich Abbey aerobic	1.37	14.7	7.75	97.19	0.8737	1.132
Middle Pond aerobic	4.43	27.2	0.91	85.94	1.523	7.691
Calwich Abbey anaerobic	13.1	112	4.56	87.69	1.046	13.89
Middle Pond anaerobic	25.1	99.7	2.62	88.6	4.776	160.9

Test Conditions	Total system extracts (FOMC)					
	$DegT_{50}$ [days]	$DegT_{90}$ [days]	χ^2%	Parent_0	alpha	beta
Calwich Abbey aerobic	39.5	507	2.44	97	0.7736	27.23
Middle Pond aerobic	30.2	126	1.98	96.32	3.715	147.2
Calwich Abbey anaerobic	116	389	1.39	95.9	80.69	13400
Middle Pond anaerobic	131	438	0.793	98.05	104.8	19700

Test Conditions	Total system extracts + MAE + NER I (FOMC)					
	$DegT_{50}$ [days]	$DegT_{90}$ [days]	χ^2%	Parent_0	alpha	beta
Calwich Abbey aerobic	54.6	1600	2.13	97.07	0.5226	19.74
Middle Pond aerobic	46.3	336	1.6	96.39	1.232	61.25
Calwich Abbey anaerobic	140	523	1.45	95.81	6.863	1310
Middle Pond anaerobic	202	686	0.423	97.55	35.57	10300

MAE – Microwave Assisted Extraction
NER I – Non Extractable Residue type I

Figure S 13 Distribution of applied radioactivity of [14C]-6-PPDQ during biodegradation in two water-sediment systems under aerobic conditions

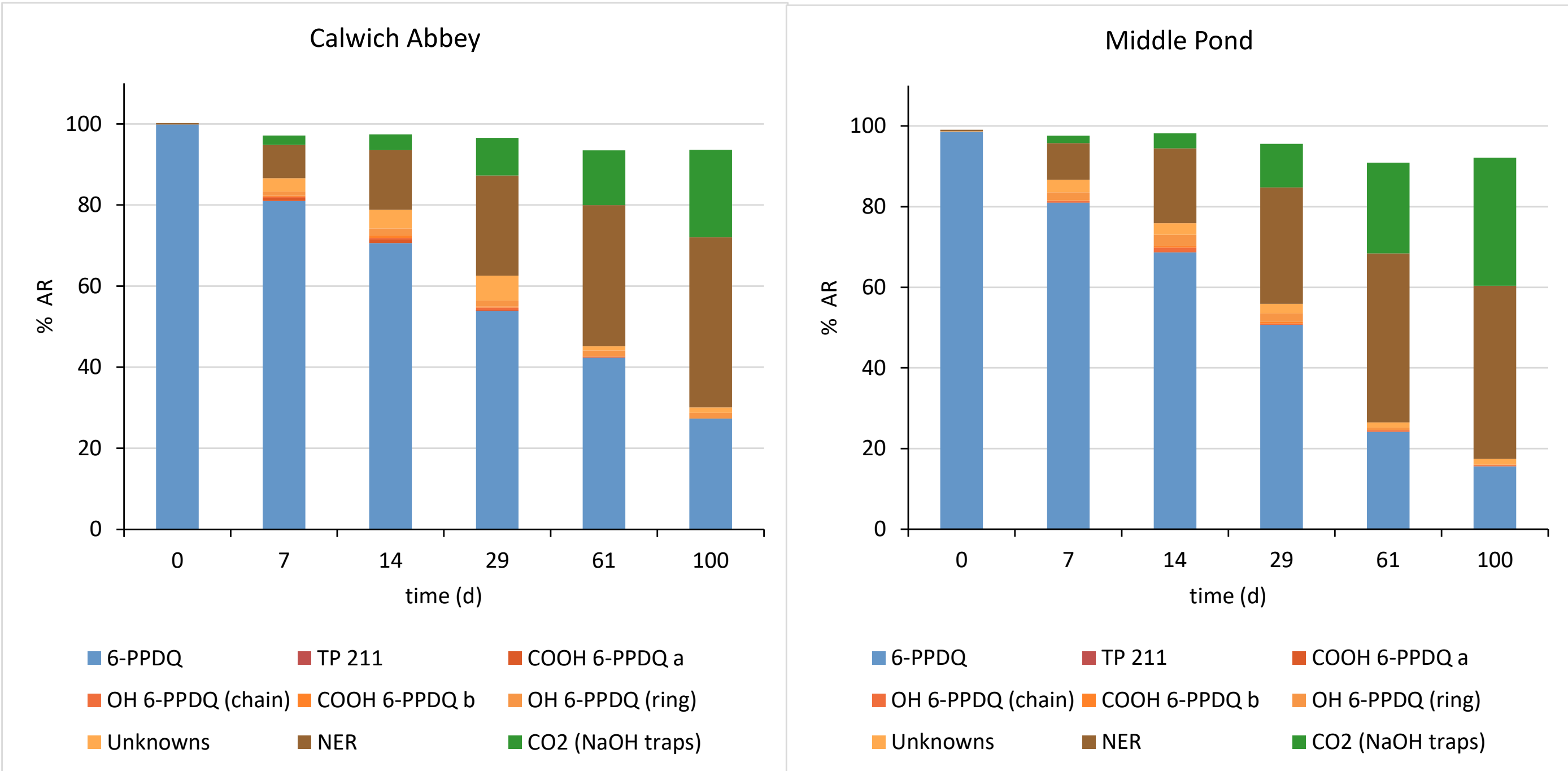

Figure S 14 Distribution of applied radioactivity of [14C]-6-PPDQ during biodegradation in two water-sediment systems under anaerobic conditions

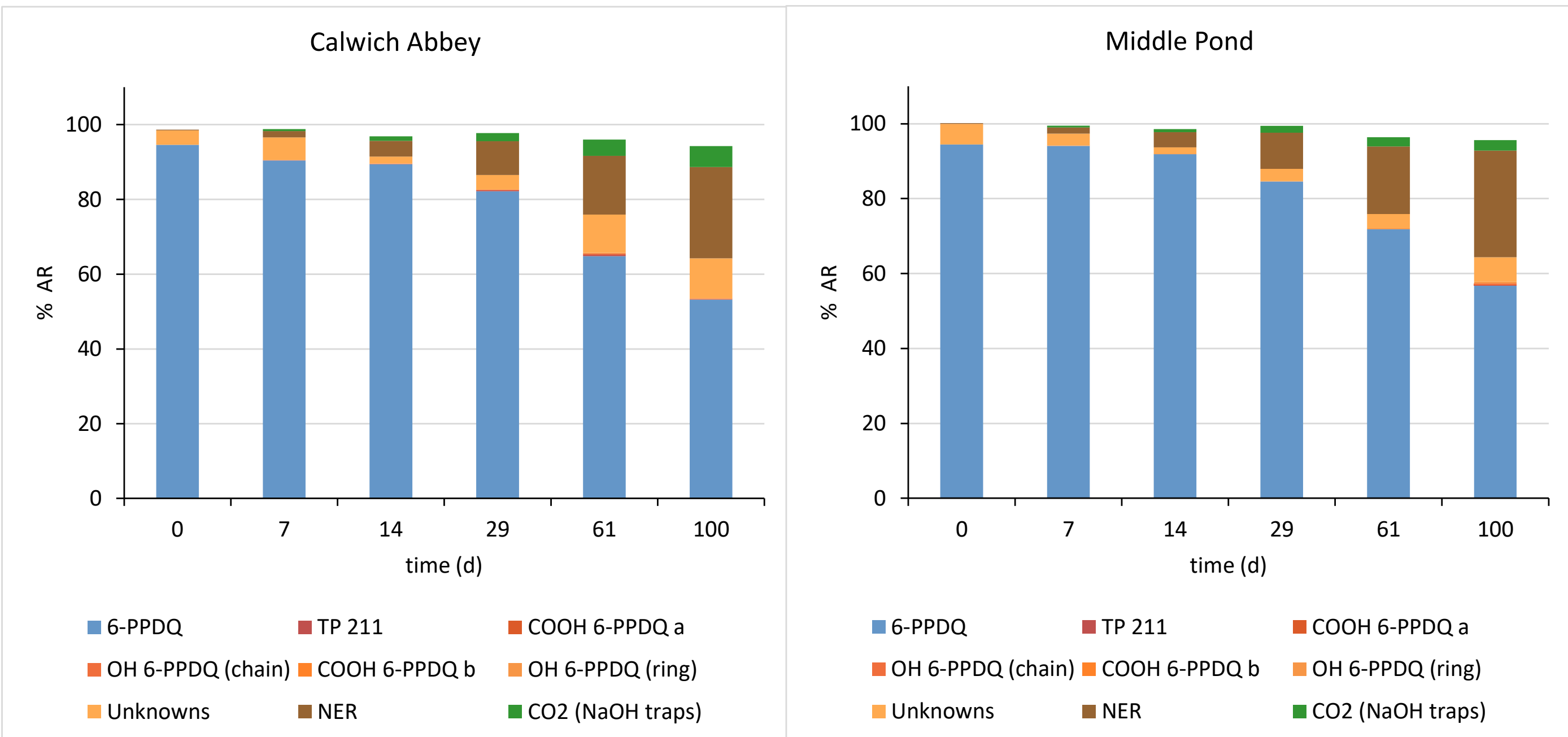

2.2.3 Chromatograms

Figure S 15 Radio-chromatograms obtained from samples of 6-PPDQ biodegradation in water-sediment system

Overlying water, 0 DAT, typical of all treatment groups

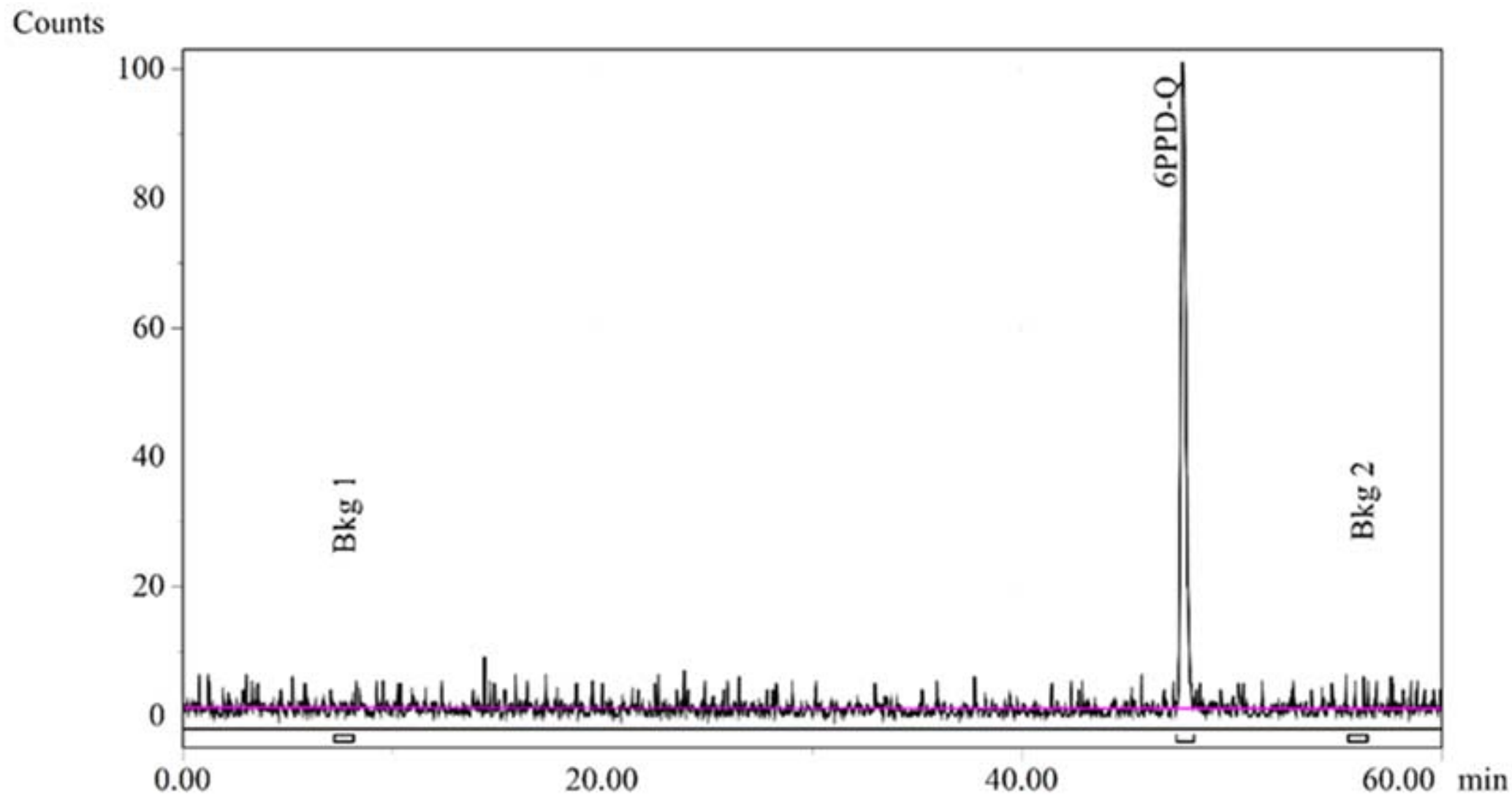

Sediment extract, 0 DAT, typical of all treatment groups

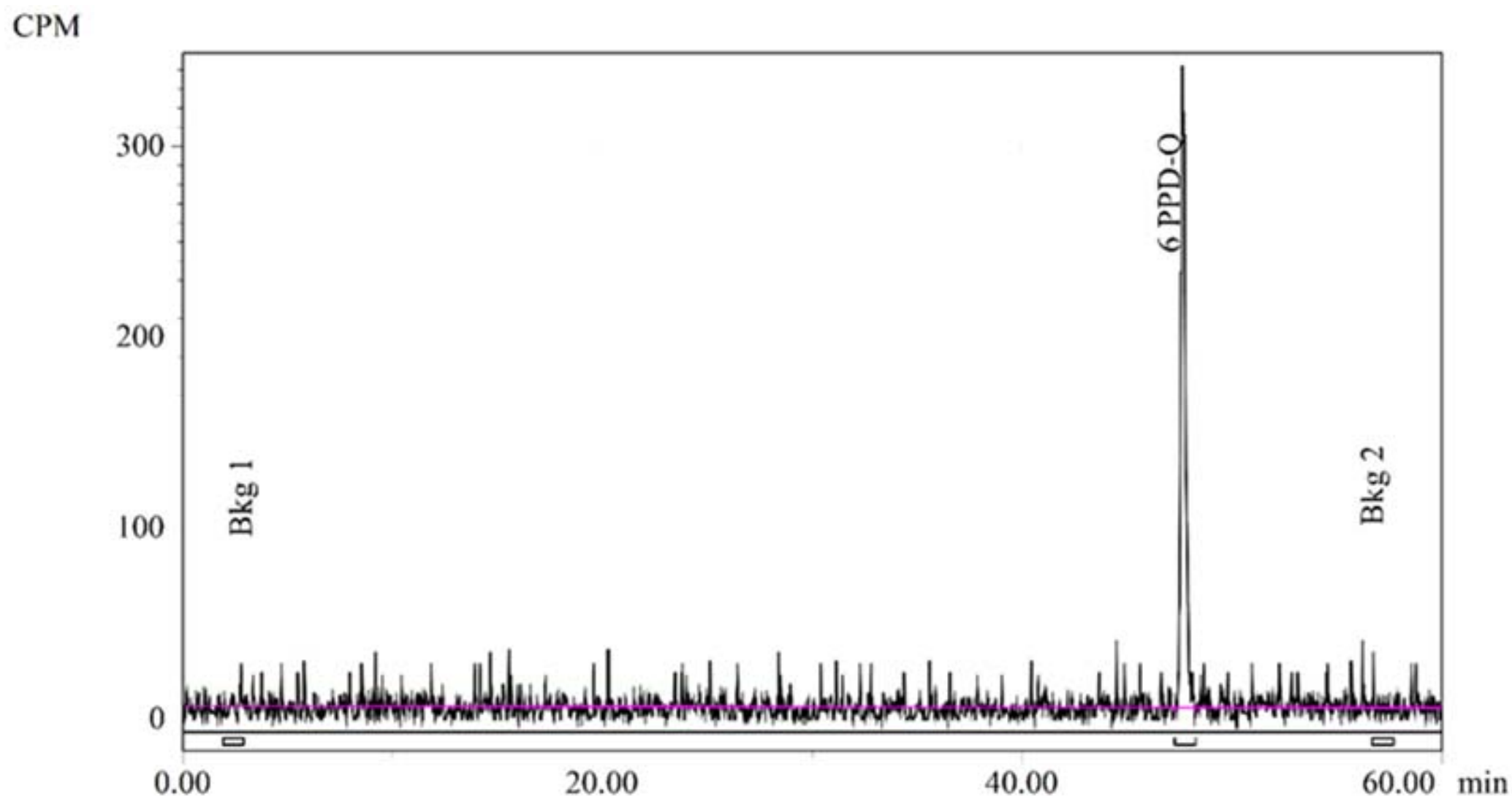

Overlying water, 14 DAT, typical of aerobic treatment groups

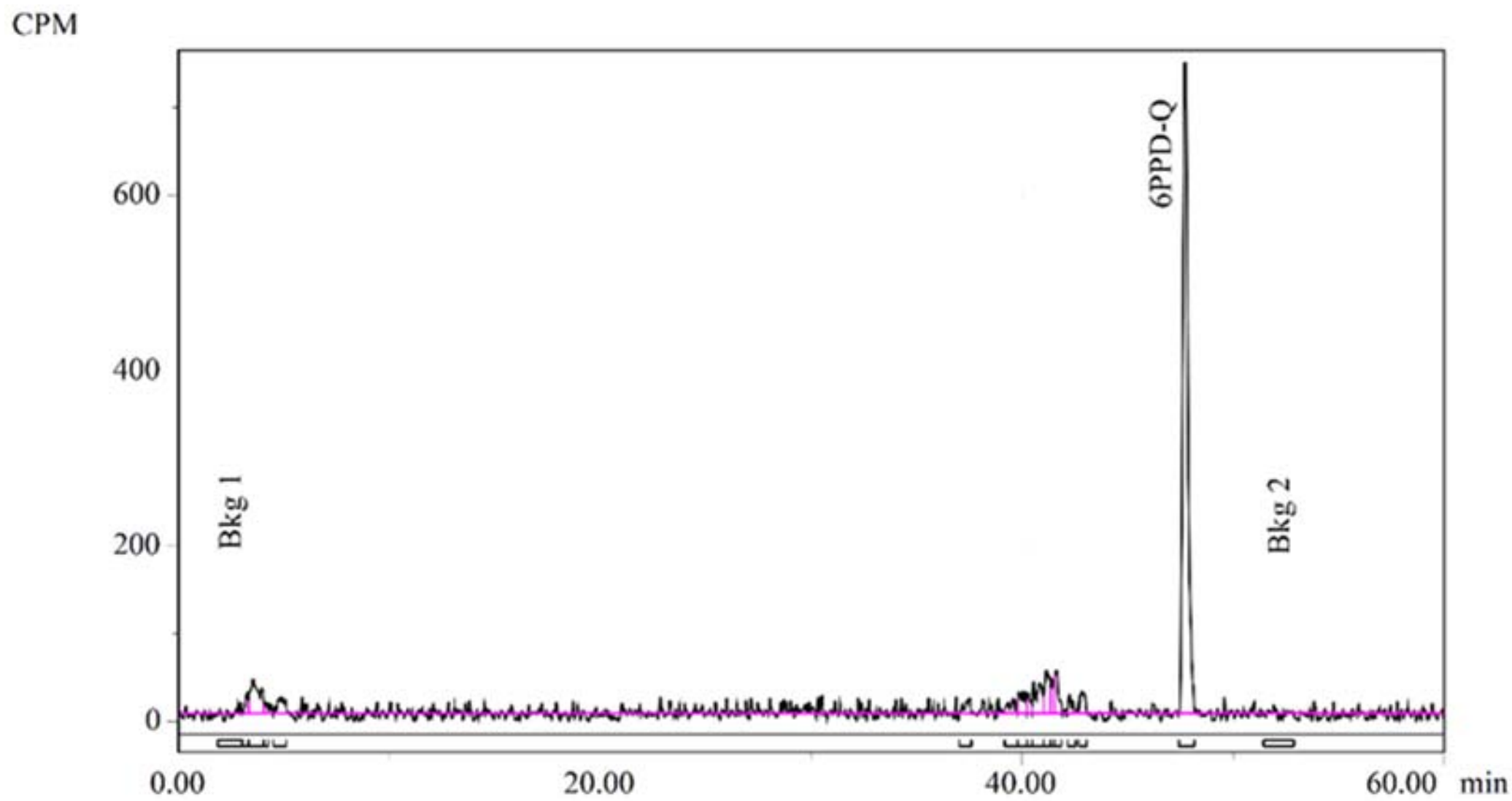

Overlying water, 100 DAT, typical of anaerobic treatment groups (15.8% AR)

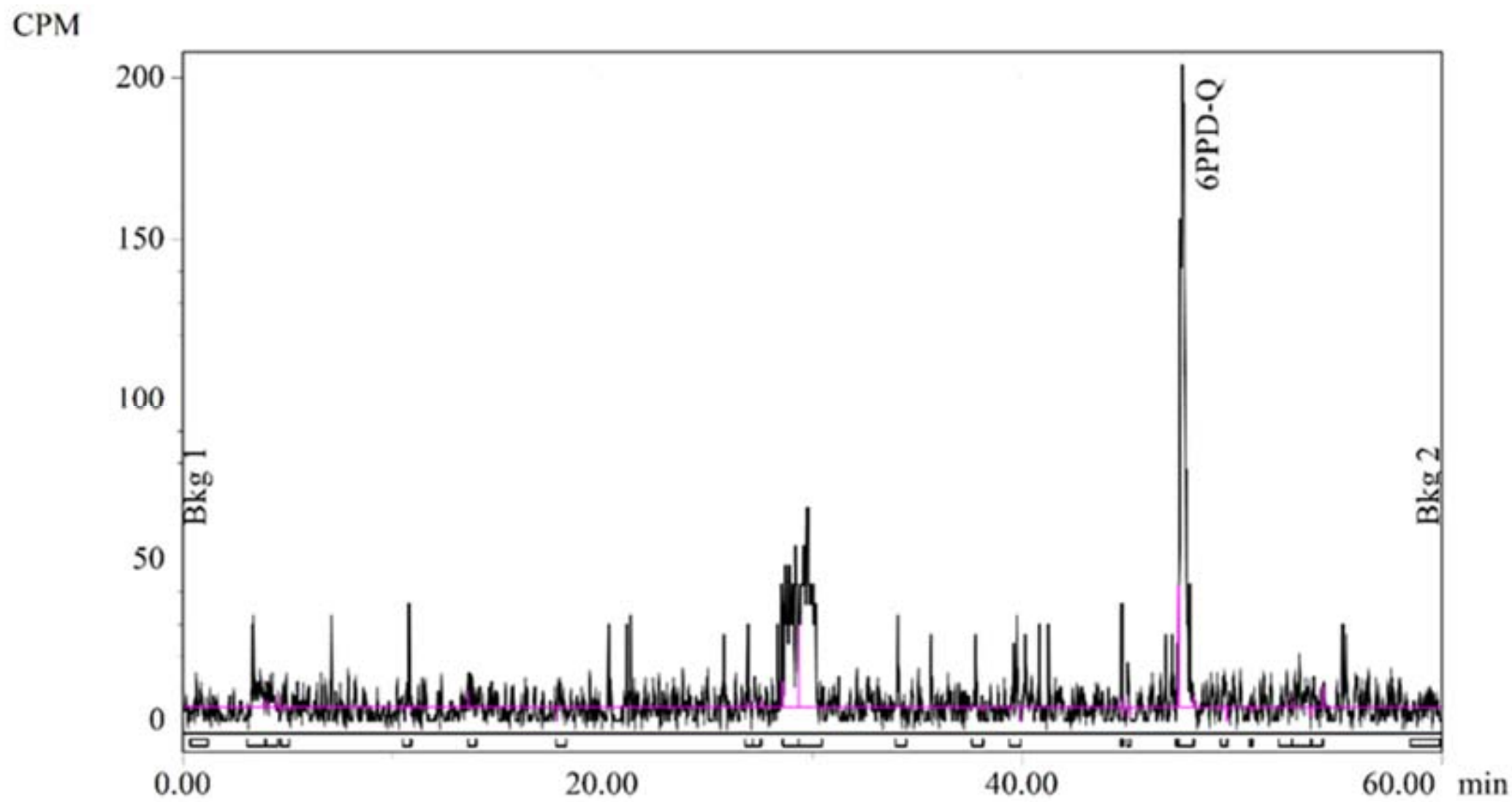

Sediment extract, 100 DAT, typical of aerobic treatment groups (16.9% AR)

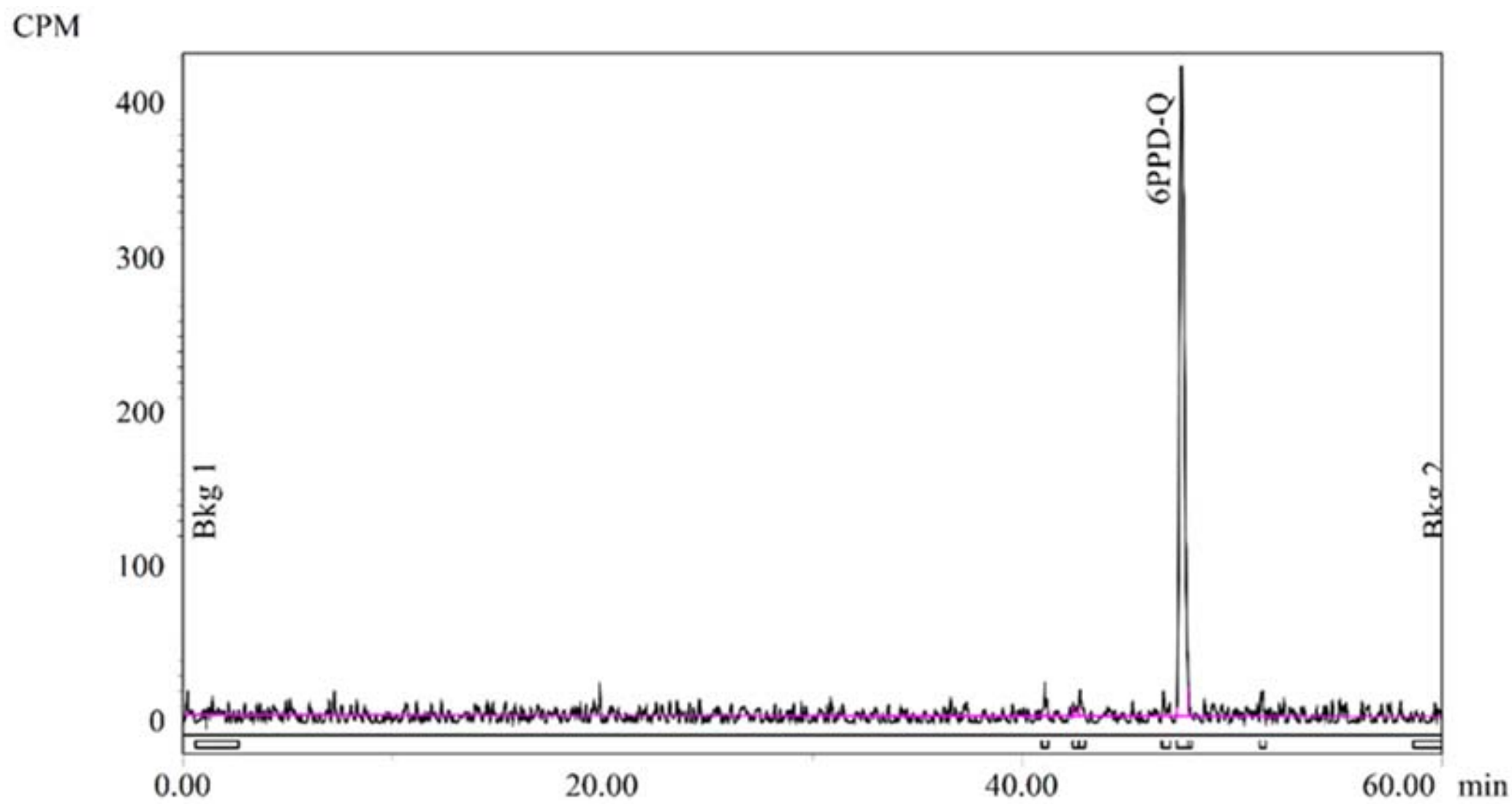

Sediment extract, 100 DAT, typical of anaerobic treatment groups (50.7% AR)

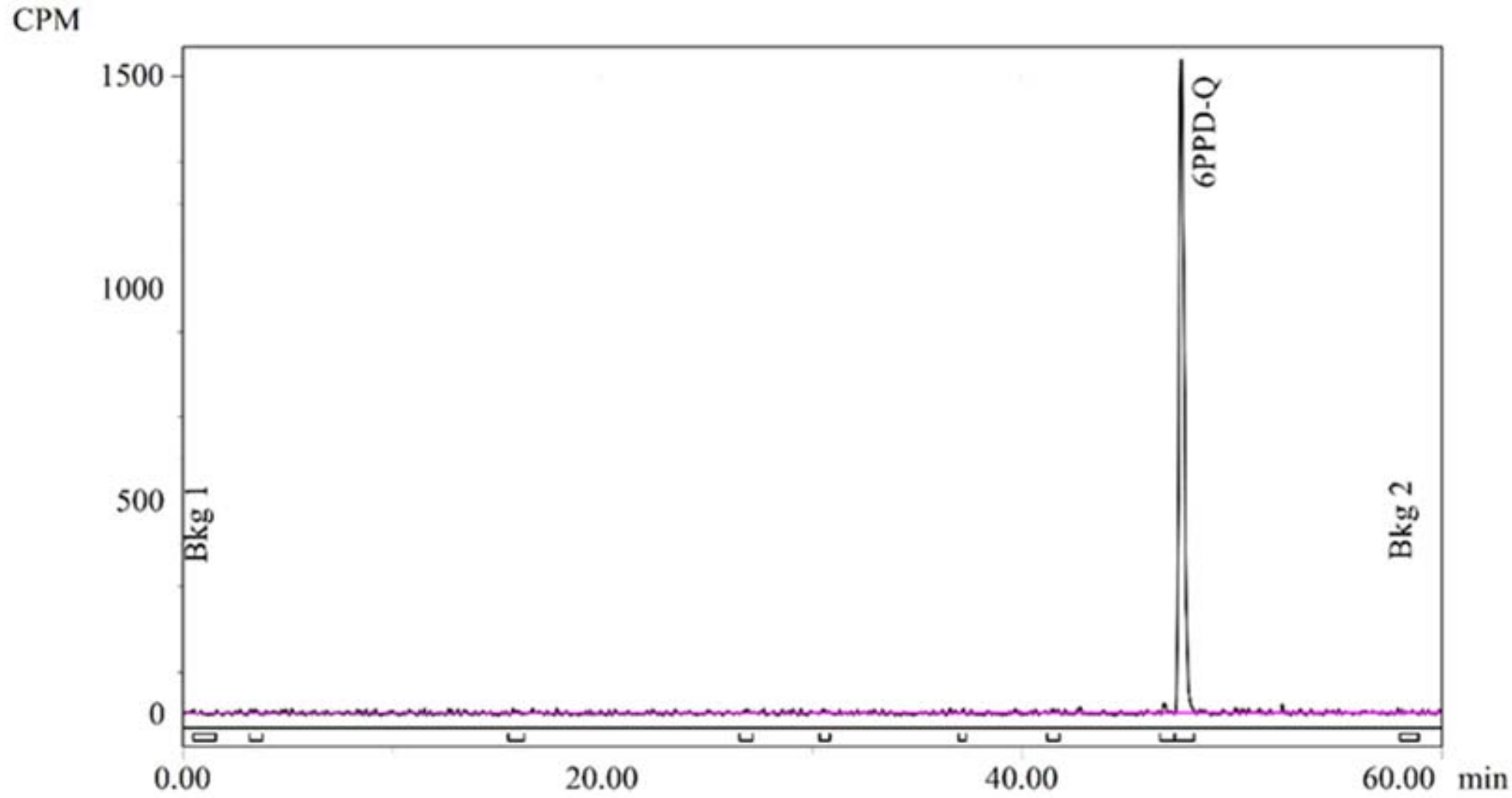

2.3 Degradation in soil

2.3.1 Recovery of applied radioactivity (AR)

Table S 18 The recovery and distribution of applied radioactivity (AR) in the different soils

System	Sampling Time (DAT/DAF)	% Applied Radioactivity in			
		Soil Extract	NER	Traps	Mass Balance
Brierlow Aerobic	0	94.6	4.6	NA	99.1
	30	60.3	24.3	13.3	97.8
	120	38.9	31.0	24.7	94.6
South Witham Aerobic	0	89.0	5.5	NA	95.9
	30	43.4	31.6	22.6	97.5
	120	14.8	38.1	42.4	95.3
Labsoil B2 Aerobic	0	98.1	1.8	NA	99.9
	30	62.7	18.1	17.6	98.3
	120	51.3	21.7	26.6	99.4
Speyer 5M Aerobic	0	95.6	2.8	NA	98.3
	30	59.9	19.8	18.3	97.9
	120	19.4	35.9	40.8	96.0
Brierlow Anaerobic	0	58.1	23.9	15.6	97.5
	29	44.7	30.3	20.9	95.8
	120	41.7	31.6	22.2	95.5
South Witham Anaerobic	0	55.8	23.8	15.2	94.8
	29	58.4	23.1	12.6	94.0
	120	39.5	39.7	17.8	96.9
Labsoil B2 Anaerobic	0	57.3	20.0	19.3	95.6
	29	54.0	21.4	21.4	96.8
	120	31.0	41.2	21.9	94.0
Speyer 5M Anaerobic	0	63.4	19.3	15.6	98.2
	29	55.8	23.3	17.3	96.2
	120	52.9	27.5	17.7	98.0

Table S 19: Percent recovery of applied radioactivity including NER I-III from additional extractions of soil treated with [14C]-6PPD-quinone and maintained under aerobic conditions at the end of the experiment.

	Percent of Applied Radioactivity			
	Brierlow	South Witham	Labsoil B2	Speyer 5M
Residue from initial extractions	33.9	39.4	22.4	36.2
Microwave Assisted Extraction	5.5	3.9	4.4	3.7
Residue from MAE [a]	27.9	34.6	16.2	30.4
NER type I	11.2	2.2	3.3	3.4
NER type II	19.9	29.8	11.9	23.9
NER type III [b]	8.6	10.9	7.1	11.5

NER Non-extractable residue
[a] Residue remaining after MAE characterised as NER Type I-III; [b] Please note that NER type III may include NER type I components as it was determined parallel to NER type I-II from the residue of MAE.

Table S 20 Percent recovery of applied radioactivity including NER I-III from additional extractions of soil treated with [14C]-6PPD-quinone and maintained under anaerobic conditions at the end of the experiment.

	Percent of Applied Radioactivity			
	Brierlow	South Witham	Labsoil B2	Speyer 5M
Residue from initial extractions	32.9	40.1	41.4	27.6
Microwave Assisted Extraction	6.8	7.0	10.2	8.8
Residue from MAE [a]	26.4	31.9	27.9	17.9
NER type I	4.9	5.4	7.0	3.8
NER type II	20.0	26.8	20.3	12.4
NER type III [b]	6.7	8.0	9.2	5.2

NER Non-extractable residue
[a] Residue remaining after MAE characterised as NER Type I-III; [b] Please note that NER type III may include NER type I components as it was determined parallel to NER type I-II from the residue of MAE.

2.3.2 Kinetic results

Table S 21 Kinetic data for [14C]-6-PPDQ in soils under aerobic and anaerobic conditions applying different kinetic models

Test Conditions	Aerobic Soil Extracts (SFO)					
	$DegT_{50}$ [days]	$DegT_{90}$ [days]	χ^2%	Parent_0	k	
Brierlow	78	259	10.1	82.45	0.00889	
South Witham	27.3	90.7	7.25	88.72	0.02539	
Labsoil B2	141	467	13	78.8	0.00494	
Speyer 5M	44.7	148	3.85	91.45	0.01552	

Test Conditions	Anaerobic Soil Extracts (SFO)					
	$DegT_{50}$ [days]	$DegT_{90}$ [days]	χ^2%	Parent_0	k	
Brierlow	394	1310	7.95	47.06	0.00176	
South Witham	267	886	5.21	55.72	0.002598	
Labsoil B2	117	389	8.2	57.37	0.005924	
Speyer 5M	448	1049	2.3	58.65	0.001546	

Test Conditions	Aerobic Soil Extracts + MAE + NER I (SFO)					
	$DegT_{50}$ [days]	$DegT_{90}$ [days]	χ^2%	Parent_0	k	
Brierlow	131	436	6.24	86.07	0.005285	
South Witham	34.7	115	8.18	87.44	0.01998	
Labsoil B2	181	602	10	81.94	0.003828	
Speyer 5M	54.1	180	4.07	90.99	0.01281	

Test Conditions	Anaerobic Soil Extracts + MAE + NER I (SFO)					
	$DegT_{50}$ [days]	$DegT_{90}$ [days]	χ^2%	Parent_0	k	
Brierlow	615	2040	5.86	56.05	0.001127	
South Witham	447	1480	3.56	62.02	0.001552	
Labsoil B2	214	710	5.33	63.8	0.003244	
Speyer 5M	887	2950	1.56	67.19	7.82×10^{-4}	

FOMC Kinetics

Test Conditions	Aerobic Soil Extracts					
	$DegT_{50}$ [days]	$DegT_{90}$ [days]	χ^2%	Parent_0	alpha	beta
Brierlow	51	>10,000	4.41	95.76	0.2844	4.886
South Witham	22.4	142	2.87	93.14	1.451	36.63
Labsoil B2	169	>10,000	5.88	97.42	0.1191	0.5017
Speyer 5M	40.4	193	3.04	93.78	2.438	122.8

Test Conditions	Anaerobic Soil Extracts					
	$DegT_{50}$ [days]	$DegT_{90}$ [days]	χ^2%	Parent_0	alpha	beta
Brierlow	>10,000	>10,000	0.848	55.85	0.02127	321,000
South Witham	267	891	5.74	55.72	184.7	71,000
Labsoil B2	117	394	9.05	57.38	64.8	10,900
Speyer 5M	>10,000	>10,000	1.56	60.31	0.08975	15.44

Test Conditions	Aerobic Soil Extracts + MAE + NER I					
	$DegT_{50}$ [days]	$DegT_{90}$ [days]	χ^2%	Parent_0	alpha	beta
Brierlow	188	>10,000	2.66	95.81	0.1966	5.698
South Witham	26.7	229	2.63	93.41	1.043	28.24
Labsoil B2	654	>10,000	4.69	97.42	0.09894	0.5935
Speyer 5M	47.6	290	2.56	94.16	1.538	83.76

Test Conditions	Anaerobic Soil Extracts + MAE + NER I					
	$DegT_{50}$ [days]	$DegT_{90}$ [days]	χ^2%	Parent_0	alpha	beta
Brierlow	>10,000	>10,000	0.798	63.95	0.01063	5.55×10^{-8}
South Witham	452	1560	3.92	62.02	22.85	14700
Labsoil B2	217	772	5.87	63.84	11.79	3.580
Speyer 5M	>10,000	>10,000	1.02	68.67	0.03715	7.389

DFOP Kinetics

Test Conditions	Aerobic Soil Extracts (DFOP)					
	$DegT_{50}$ [days]	$DegT_{90}$ [days]	χ^2%	k1	k2	g
Brierlow	62.4	310	3.26	2.435	0.0065	0.2501
South Witham	22.8	192	3.25	0.04115	0.003808	0.793
Labsoil B2	141	1110	5.39	0.1747	0.001659	0.3683
Speyer 5M	42	156	2.19	2.711	0.01419	0.09243

Test Conditions	Anaerobic Soil Extracts (DFOP)					
	$DegT_{50}$ [days]	$DegT_{90}$ [days]	χ^2%	k1	k2	g
Brierlow	970	4720	0.605	0.3859	0.000429	0.2416
South Witham	267	886	6.55	0.05954	0.002598	3.77×10^{-11}
Labsoil B2	117	389	10.3	0.005927	0.005924	0.01397
Speyer 5M	>10,000	>10,000	1.51	0.02623	1.72×10^{-11}	0.1787

Test Conditions	Aerobic Soil Extracts + MAE + NER I (DFOP)					
	$DegT_{50}$ [days]	$DegT_{90}$ [days]	χ^2%	k1	k2	g
Brierlow	123	524	1.98	0.353	0.004011	0.1814
South Witham	26.7	464	2.65	0.03831	0.00186	0.7631
Labsoil B2	235	1390	4.3	0.1668	0.001397	0.3056
Speyer 5M	50.7	190	2.64	0.3846	0.01158	0.1003

Test Conditions	Anaerobic Soil Extracts + MAE + NER I (DFOP)					
	$DegT_{50}$ [days]	$DegT_{90}$ [days]	χ^2%	k1	k2	g
Brierlow	2730	>10,000	0.739	0.376	1.77×10^{-4}	0.1885
South Witham	447	1480	4.47	0.05907	0.001552	1.10×10^{-9}
Labsoil B2	225	>10,000	6.7	0.004049	7.21×10^{-10}	0.8357
Speyer 5M	>10,000	>10,000	0.96	0.03399	2.65×10^{-13}	0.09716

Figure S 16 Distribution of applied radioactivity of [14C]-6-PPDQ during biodegradation in four different soils under aerobic conditions

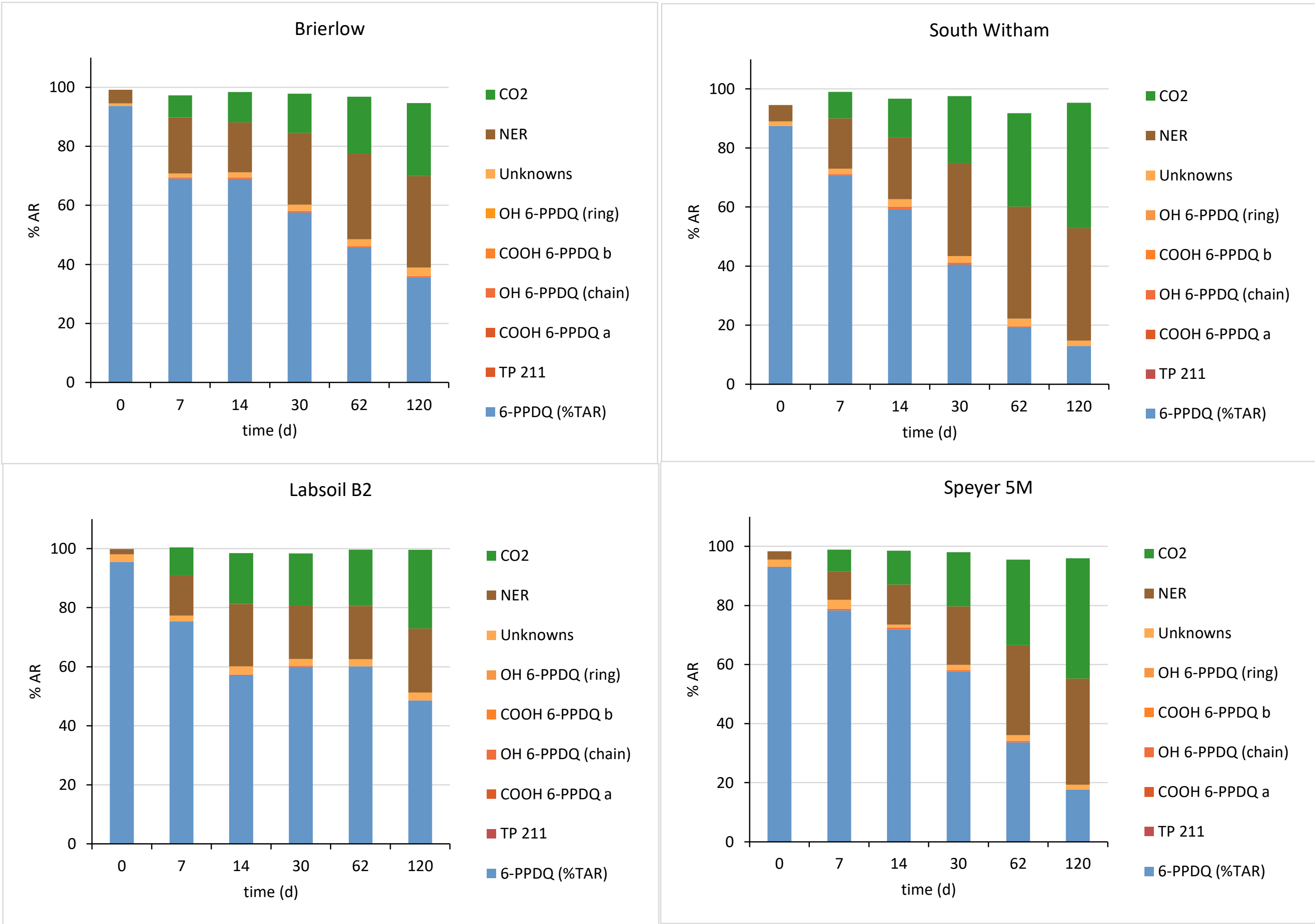

Figure S 17 Distribution of applied radioactivity of [14C]-6-PPDQ during biodegradation in four different soils under anaerobic conditions. AR at day of flooding set to 100%; time = time after flooding.

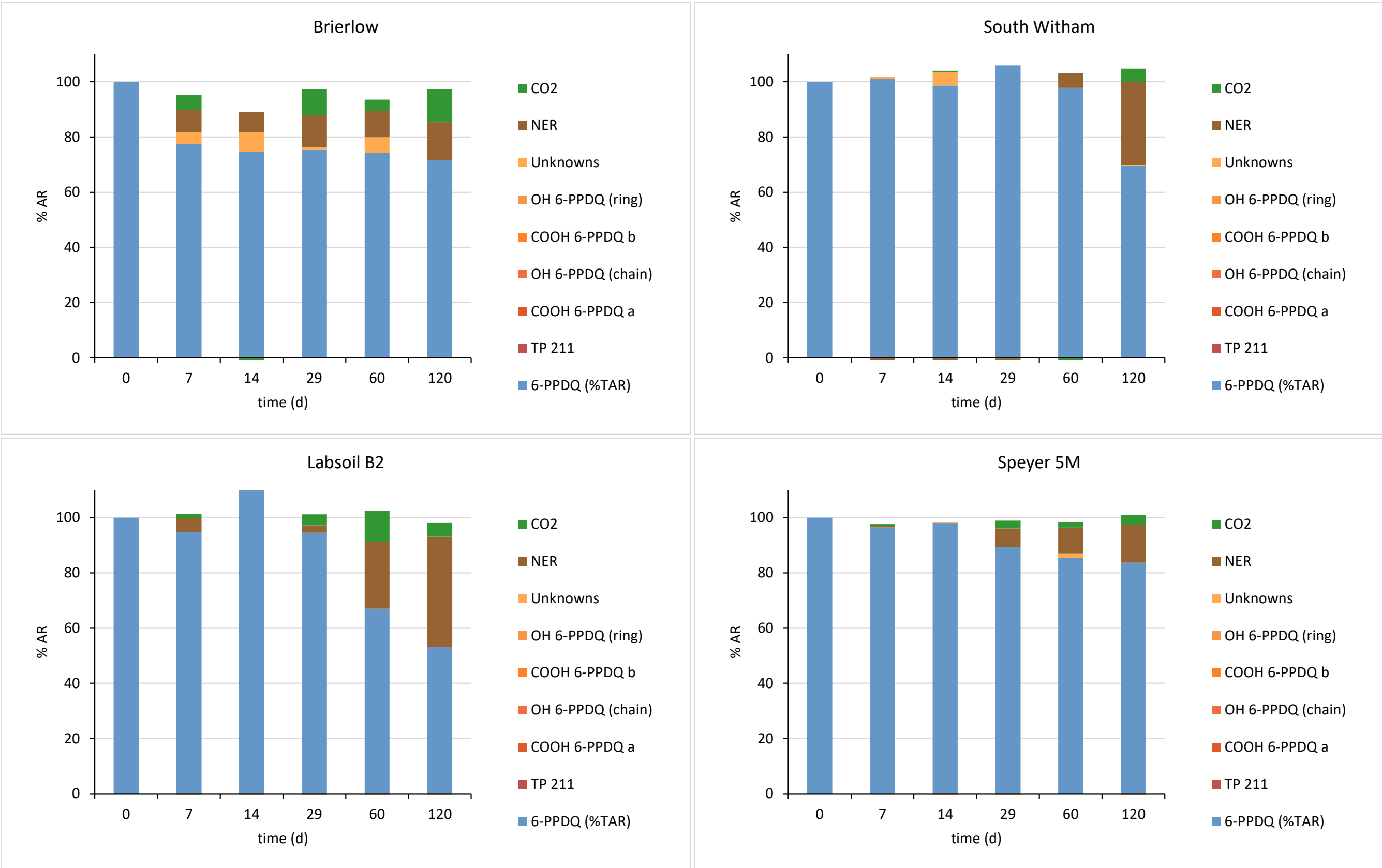

2.3.3 Chromatograms

Figure S 18 Radio-chromatograms obtained from samples of 6-PPDQ biodegradation in soil

Soil extract, 0 DAT, typical of all treatment groups (95.4% AR)

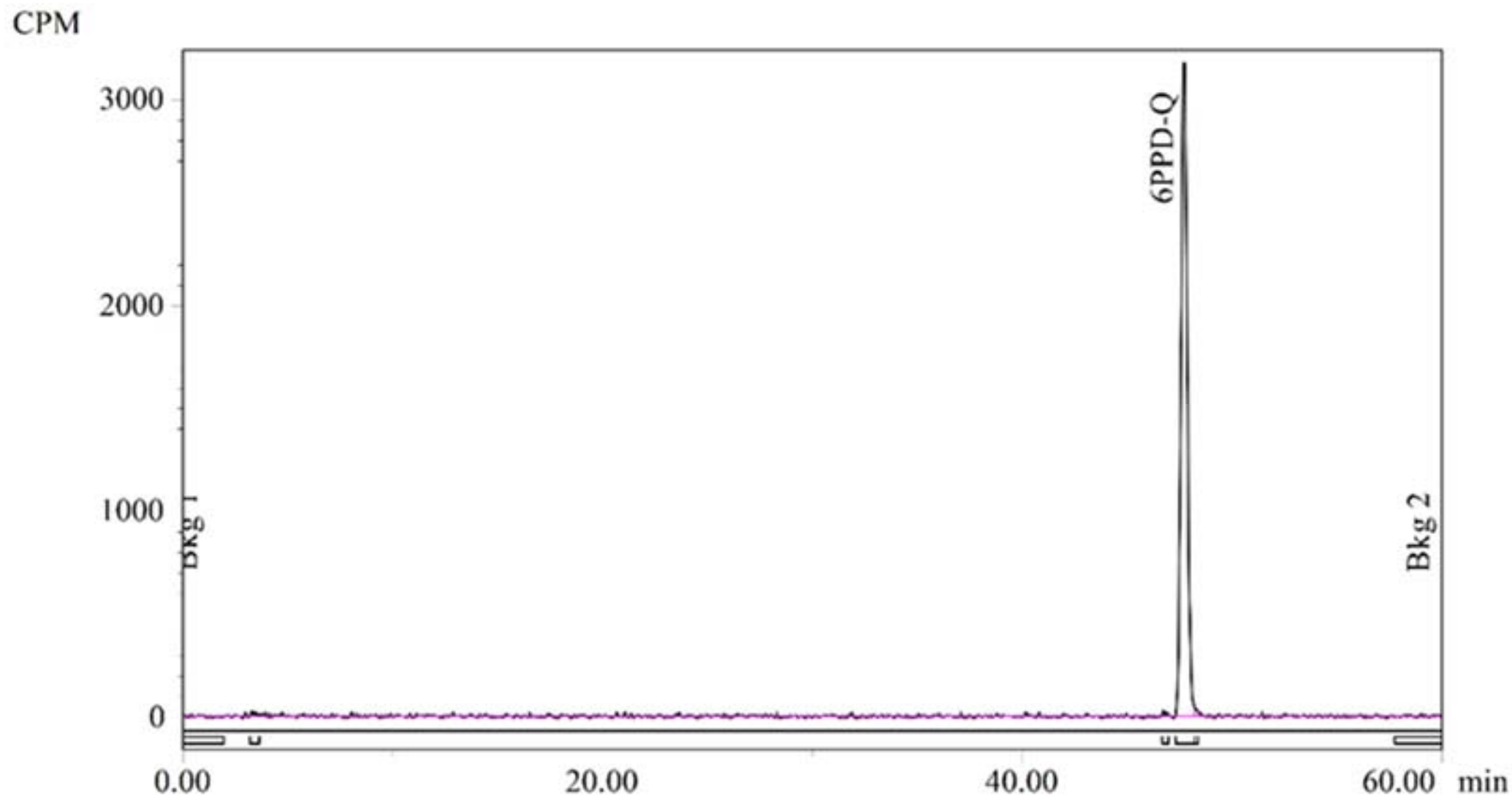

Soil extract, 30 DAT (flooding), typical of aanaerobic treatment groups (57.0% AR)

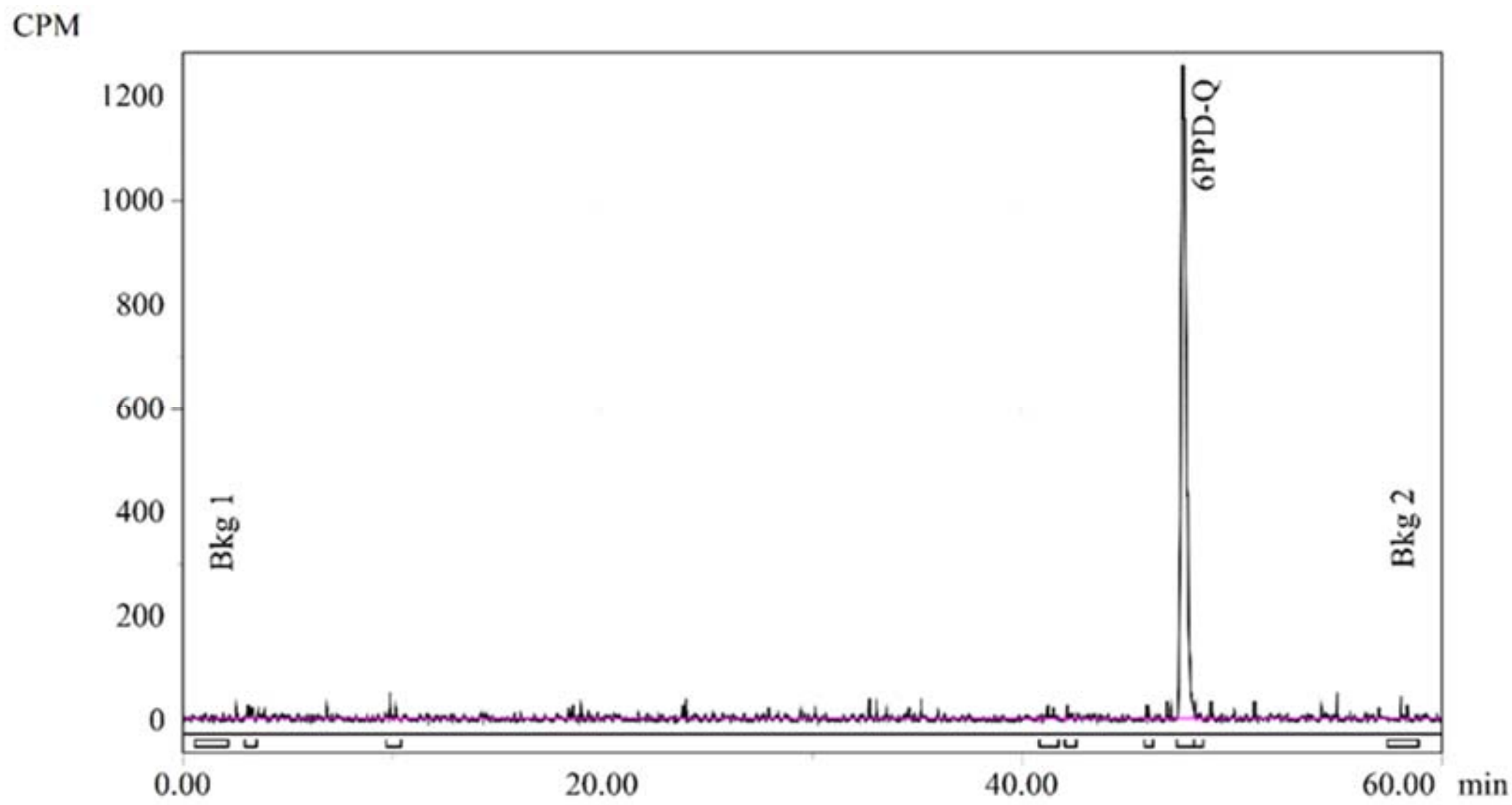

Soil extract, 120 DAT, typical of aerobic treatment groups (50.2% AR)

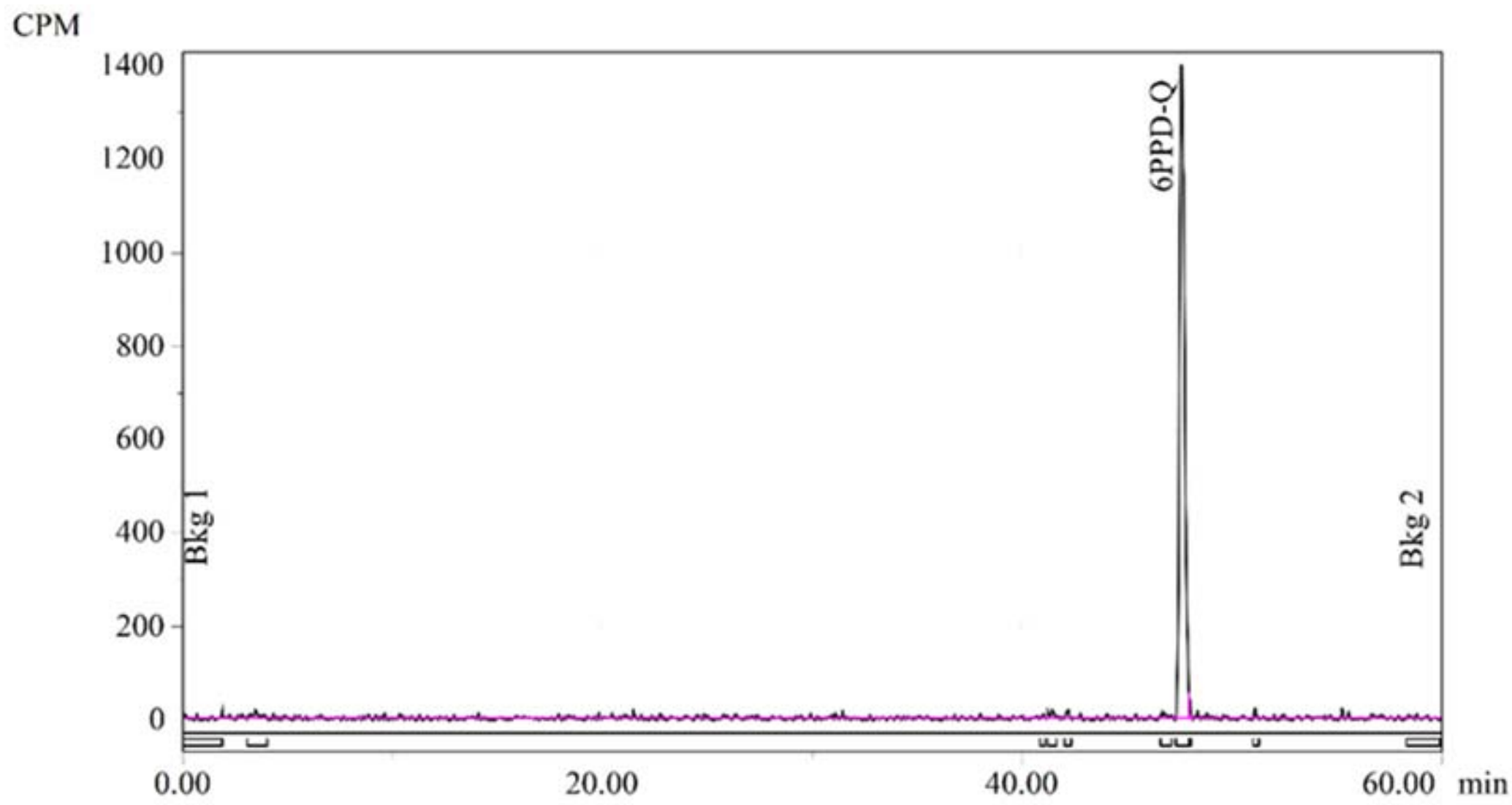

Soil extract, 120 DAF, typical of anaerobic treatment groups (41.5% AR)

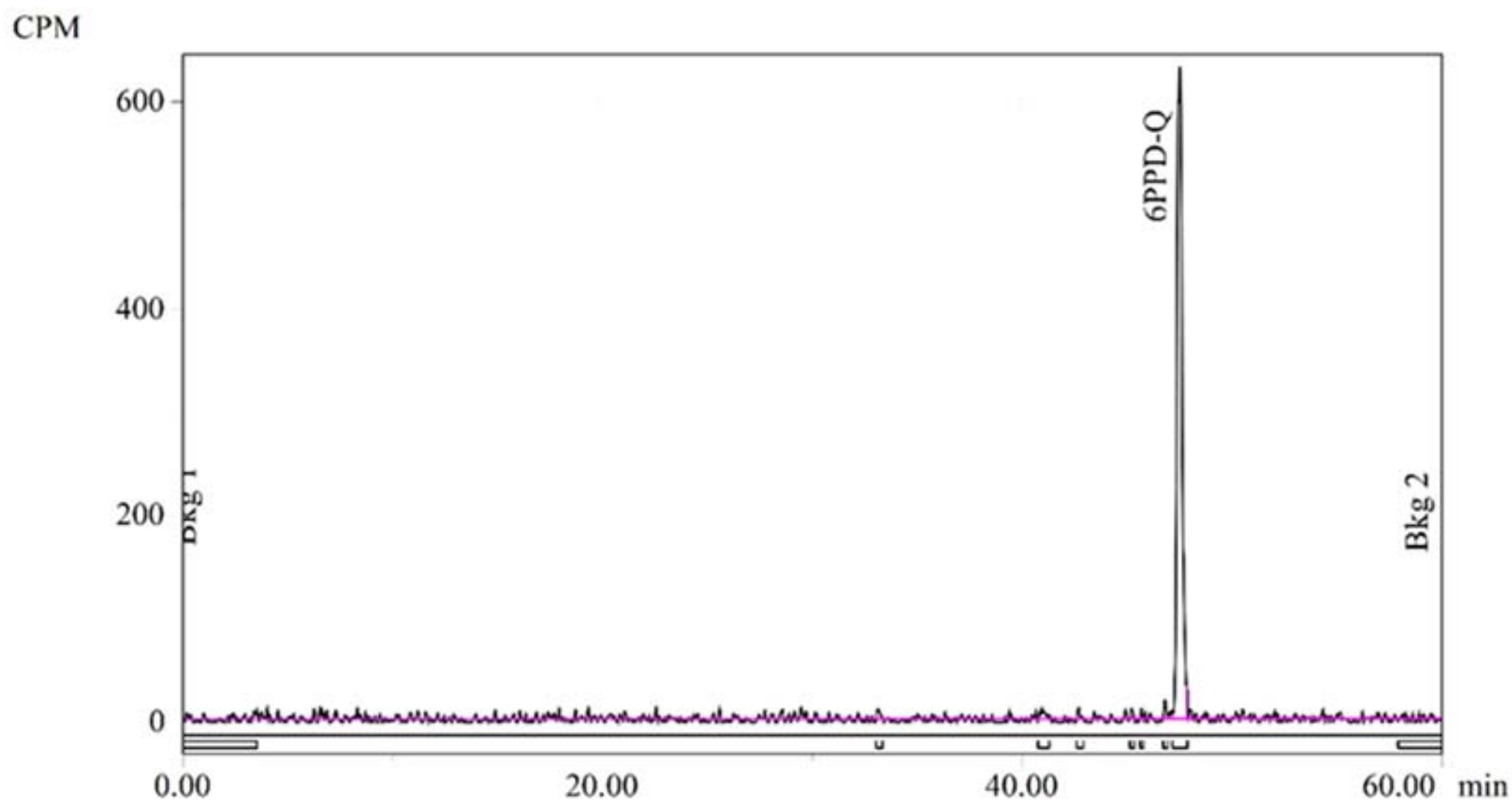

2.4 Comparison of the products of 6-PPDQ transformation

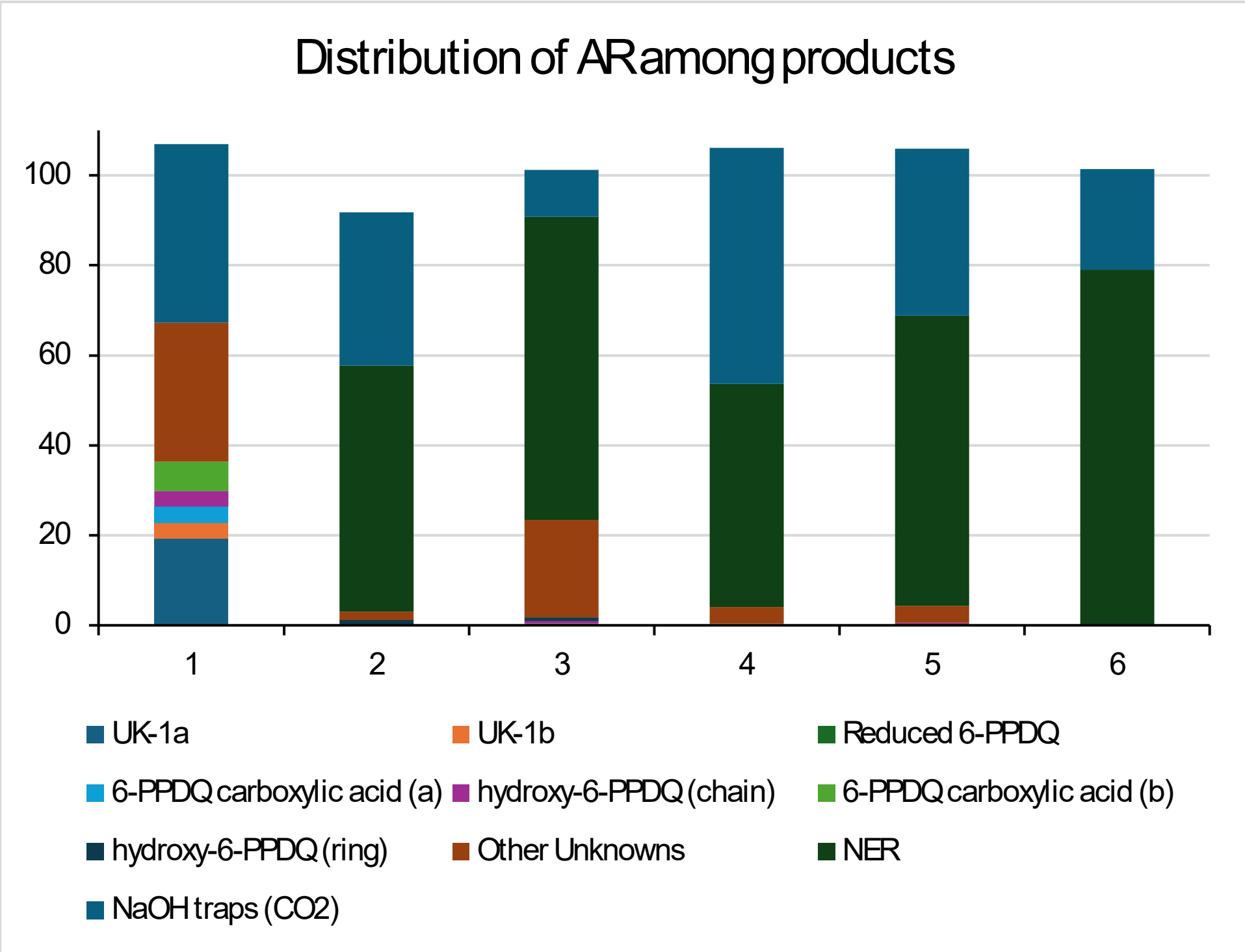

Figure S 19 Percentage distribution of the products of 6-PPDQ (as % AR of 6-PPDQ degraded).

1. surface water aerobic; 2. water/sediment aerobic; 3. water/sediment/anaerobic; 4. soil aerobic; 5. soil aerobic/anaerobic; 6: soil anaerobic.

3. References:

ECHA 2023. Guidance on Information Requirements and Chemical Safety Assessment Chapter R.11: PBT/vPvB assessment. Version 4.0, December 2023. Ref. No. ECHA-23-H-10-EN. https://doi.org/10.2823/312974

Foscari, A., Seiwert, B., Zahn, D., Schmidt, M. and Reemtsma, T. 2024. Leaching of tire particles and simultaneous biodegradation of leachables. Water Res. 253, 121322.

Han, L., Seiwert, B., Lichtenwald, E., Weyrauch, S., Zahn, D. and Reemtsma, T. 2025. Biodegradation pathways and products of tire-related phenylenediamines and phenylenediamine quinones in solution - a laboratory study. Water Res. 286, 124235.

Shen D., Shi, Q., Zhang, J., Sy, N.D., Yates, R., Wang, W., Gan J. 2025. Transformations of 6PPD and 6PPD-quinone in soil under redox-driven conditions: Kinetics, product identification, and environmental implications. Environ. Int. 200, 109532.
